# Association of Renalase SNPs rs2296545 and rs2576178 with the Risk of Hypertension: A Meta-Analysis

**DOI:** 10.1371/journal.pone.0158880

**Published:** 2016-07-19

**Authors:** Yong-Bo Lv, Yang Wang, Wang-Ge Ma, Ding-Yi Yan, Wen-Ling Zheng, Chao Chu, Tong-Shuai Guo, Zu-Yi Yuan, Jian-Jun Mu

**Affiliations:** 1 Department of Cardiology, First Affiliated Hospital of Medical School, Xi’an Jiaotong University, Xi’an, China; 2 Key Laboratory of Molecular Cardiology of Shaanxi Province, Xi’an, China; Yale School of Public Health, UNITED STATES

## Abstract

**Background/Aims:**

Two renalase single nucleotide polymorphisms (SNPs) rs2296545 and rs2576178 have been reported to be associated with the susceptibility to hypertension (HT). Given the inconsistent results, we conducted a meta-analysis to assess the association between these two SNPs and the risk of HT.

**Methods:**

Electronic databases were systematically searched to find relevant studies. Subgroup analysis was conducted according to the different concomitant diseases and ethnicities in the study population. Pooled odds ratios (OR) and 95% confidence intervals (CI) were calculated using fixed-effect or random-effect models.

**Results:**

A total of six case–control studies on rs2296545 and six studies on rs2576178 were included. In the combined analysis, results showed a significant association between SNP rs2296545 and risk of HT in all genetic models (dominant model CG+CC/GG: OR = 1.43, 95% CI = 1.24–1.65; recessive model CC/CG+GG: OR = 1.36, 95% CI = 1.09–1.69; codominant model CC/GG: OR = 1.63, 95% CI = 1.20–2.20, CG/GG: OR = 1.30, 95% CI = 1.12–1.52; allelic model C/G: OR = 1.29, 95% CI = 1.10–1.51). In subgroup analysis, we observed a significant association between rs2296545 and risk of essential HT. Although we did not observe an association between rs2576178 polymorphism and HT in the combined analysis, an increased risk was observed in the essential HT patients versus healthy controls (subgroup 1) analysis under the dominant, recessive, and codominant genetic models.

**Conclusions:**

Renalase gene rs2296545 polymorphism is significantly associated with increased risk of HT, whereas rs2576178 polymorphism may not be associated with the susceptibility to HT.

## Introduction

Hypertension (HT) is not only a common chronic disease but also a major risk factor for cardiac–cerebral vascular disease and chronic kidney disease (CKD) [1]. The number of hypertensive patients is estimated to increase to 1.56 billion in 2025; thus, HT is a serious public health problem worldwide [2]. HT is considered to be a multifactorial disease caused by environmental, metabolic, and genetic determinants [3]. Several studies have shown that genetic factors contribute up to 30–50% of the pathogenesis of this disorder; thus, identifying susceptible genes and taking early intervention to prevent target organ damage and reduce mortality are necessary [4].

Renalase (gene name: *RNLS*), a 342-amino-acid flavoprotein with monoamine oxidase activity discovered in 2005, is highly expressed in the kidney and heart, circulates in the blood and urine, metabolizes circulating catecholamines, and regulates blood pressure (BP) and cardiovascular function [5–7]. Experimental data provided evidence that recombinant renalase exerts powerful and rapid hypotensive effects on rats and is mediated by the degradation of circulating catecholamines, which would be expected to decrease cardiac contractility and heart rate [6]. Recently, researchers observed that renalase can promote cell and organ survival through a receptor-mediated process that is independent of its intrinsic enzymatic activities; further study revealed that the plasma membrane calcium-ATPase isoform (PMCA4b) functions as a renalase receptor and mediates renalase-dependent mitogen activated protein kinase signaling and cytoprotection [8, 9]. Renalase deficiency has also been reported to be associated with CKD, heart disease, diabetes, stroke, and HT [10–13].

Currently, studies revealed that renalase gene variation, particularly single nucleotide polymorphisms (SNPs) rs2296545 and rs2576178, may contribute to HT in humans [14–22]. Rs2576178 is located at the 5‘flanking regions of *RNLS*, rs2296545 is located in the exon 2 of *RNLS*, and they are in low degree of linkage disequilibrium. rs2576178 may influence the initiation of transcription or differential splicing, and rs2296545 variants, especially CC genotype, results in a conserved amino acid change at amino acid 37 (glutamic to aspartic acid), which lead to functional enzymatic changes [16, 18]. Zhao et al. [16] first revealed that SNPs rs2296545 and rs2576178 are associated with susceptibility to HT in the Chinese population. Zhang et al. [20] reported the association between SNP rs2296545 and risk of HT in type 2 diabetes mellitus (T2DM) patients. However, few studies revealed that *RNLS* polymorphism is not associated with the risk of HT [22, 23]. Therefore, we conducted this meta-analysis to verify the association of these two SNPs with the risk of HT.

## Methods and Materials

This review conformed to the Preferred Reporting Items for Systematic Review and Meta-Analyses guidelines [24] (**S1 File**). An unpublished protocol was prepared for internal comment.

### Literature and search strategy

A systematic literature search was conducted independently by two investigators (Yong-Bo Lv and Yang Wang) in PubMed, Embase, Chinese National Knowledge Infrastructure (CNKI), Chinese Biomedical Literature Database, VIP (Chinese), and WanFang (Chinese) Database, with the last search update on January 1, 2016. Only studies in English and Chinese were selected. The terms “(renalase gene) and (polymorphism OR variation) and (hypertension OR high blood pressure)” or equivalent Chinese terms were used to search the databases. The reference lists of the included studies and recent reviews were also manually searched for further relevant studies.

### Inclusion and exclusion criteria

Eligible studies in this meta-analysis must meet the following inclusion criteria: (1) evaluation of the association between *RNLS* SNP rs2296545/rs2576178 and risk of HT; (2) case–control studies or nested case–control studies; (3) HT was defined as systolic blood pressure (SBP) ≥140 mmHg and/or diastolic blood pressure (DBP) ≥90 mmHg or treatment with antihypertensive medication; and (4) detailed genotype data could be acquired to calculate the odds ratios (OR) and 95% confidence intervals (CI). The exclusion criteria are as follows: (1) case report, review, comments, and editorial and (2) studies with no detailed genotype data. Study selection was achieved by two investigators independently (Yong-Bo Lv and Yang Wang) according to the inclusion and exclusion criteria by screening the title, abstract, and full text.

### Data extraction

The following information were collected from the included studies: name of first author, year of publication, country of origin, ethnicity, characteristics of the cases and controls, genotype frequency in the cases and controls for rs2296545 and rs2576178, and evidence of Hardy–Weinberg equilibrium (HWE) in the controls. Any dispute was solved by discussion.

### Quality assessment

The quality of the included studies was assessed by a modified version of the Newcastle–Ottawa scale (NOS) for genetic association studies [25, 26] (**S2 File**). A “star system” is utilized for the assessment of study quality, such that a study can be awarded a maximum of one star for each numbered item within the selection and exposure categories, whereas a maximum of two stars can be assigned for the comparability category. Only studies in which most of the nine items on the modified NOS were deemed satisfactory (score of 5 or higher) were considered of high quality.

### Statistics

The HWE was evaluated for each study by chi-square test in the control groups, and p < 0.05 was considered a significant departure from HWE. The OR and 95% CI were calculated to evaluate the strength of the association between rs2296545/rs2576178 polymorphisms and susceptibility to HT. A Z-test p value of less than 0.05 was considered statistically significant for the corresponding summary OR. Pooled ORs were calculated for the dominant model (rs2296545: CG+CC/GG; rs2576178: AG+GG/AA), recessive model (rs2296545: CC/CG+GG; rs2576178: GG/AG+AA), codominant model (rs2296545: CC/GG, CG/GG; rs2576178: GG/AA, AG/AA), and allelic comparison (rs2296545: C/G; rs2576178: G/A). Heterogeneity was evaluated by Q statistic (significance level of p < 0.1) and I^2^ statistic (greater than 50% as evidence of significant inconsistency). In the present study, I^2^ statistic is been used as major indicator for heterogeneity, due to the fewer number of included studies. When heterogeneity exists (I^2^ > 50%), we used the random-effects (RE) model; Otherwise, we used the fixed-effects (FE) model. Subgroup analysis was conducted according to the different concomitant diseases and ethnicities in the study population. The present meta-analysis contains the following subgroups: by concomitant diseases: ① essential hypertension (EH) patients versus healthy controls, ② T2DM patients with HT versus T2DM patients without HT, ③ CKD patients with HT versus CKD patients without HT, and ④ stroke patients with HT versus stroke patients without HT; by ethnicities: Asians and Caucasians. Potential publication bias was detected by funnel plot. Asymmetry of the funnel plot indicated the possible publication bias. All statistical analyses were conducted using the RevMan 5.3 software.

## Results

### Characteristics of eligible studies

**Fig 1:**
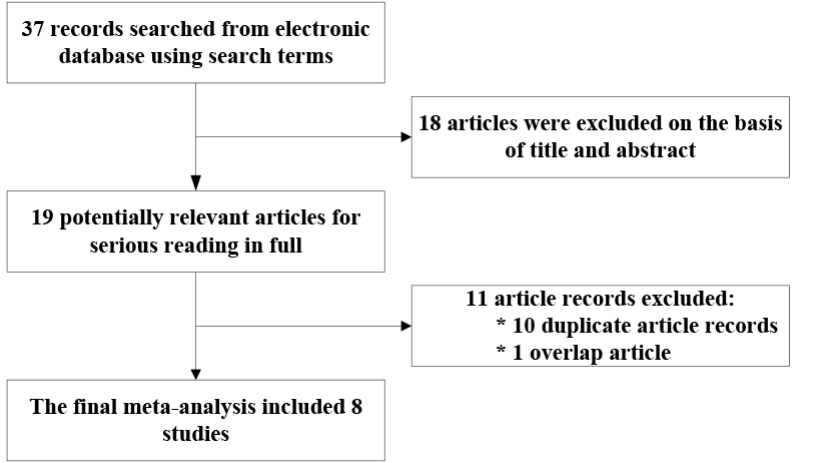
Flowchart depicting the selection of articles for the meta-analysis.

A total of 37 article records were searched from the electronic databases (PubMed = 11, Embase = 20, CNKI = 4, and WanFang = 2). The details of the selection process are presented in the flowchart shown in **Fig 1 and S3 File**. Among eight included studies, six studies were written in English [14–19] and two studies were written in Chinese [20, 21]. The baseline characteristics of all included studies are summarized in **Table 1**. In four studies [14, 16, 18, 19], genotype frequencies of rs2296545 and rs2576178 were presented separately; thus, each of them were treated as separate studies. Therefore, 6 included studies involving 2,890 cases and 2,279 controls for rs2296545 and 6 included studies involving 2,871 cases and 2,121 controls for rs2576178 were analyzed in this meta-analysis (**S1 and S2 Tables**).

**Table 1:** Characteristics of studies included in the meta-analysis.

Study ID	Country	Ethnicity	Genotypes for cases			Genotypes for controls			HWE test	mNOS
**rs2296545(C/G)**			CC	CG	GG	CC	CG	GG		
Qi Zhao et al. 2007	China	Asians	481	641	193	391	619	257	0.67	7
M. Buraczynska et al. 2011	Polish	Caucasians	265	280	136	59	106	46	0.89	6
RuYou Zhang et al. 2013	China	Asians	98	149	60	72	88	34	0.43	7
XiaoGang Li et al. 2014	China	Asians	78	93	31	67	111	54	0.54	7
Noha A. Rezk et al. 2015	Egypt	Caucasians	11	34	50	5	25	53	0.34	5
Kun Yu et al. 2015	China	Asians	119	132	39	87	133	72	0.33	6
**rs2576178(G/A)**			GG	AG	AA	GG	AG	AA		
Qi Zhao et al. 2007	China	Asians	367	702	242	298	641	321	0.53	7
M. Buraczynska et al. 2011	Polish	Caucasians	95	314	272	34	101	76	0.99	6
Anna Stec et al. 2012	Polish	Caucasians	15	81	104	12	51	106	0.1	5
RuYou Zhang et al. 2013	China	Asians	85	151	72	63	93	37	0.8	7
XiaoGang Li et al. 2014	China	Asians	43	112	47	54	119	59	0.7	7
Rong Zhang et al. 2015	China	Asians	58	87	24	17	23	16	0.18	5

HWE: Hardy–Weinberg equilibrium for controls. mNOS: Modified Newcastle–Ottawa quality scale.

### Association between rs2296545 polymorphism and HT susceptibility

**Fig 2:**
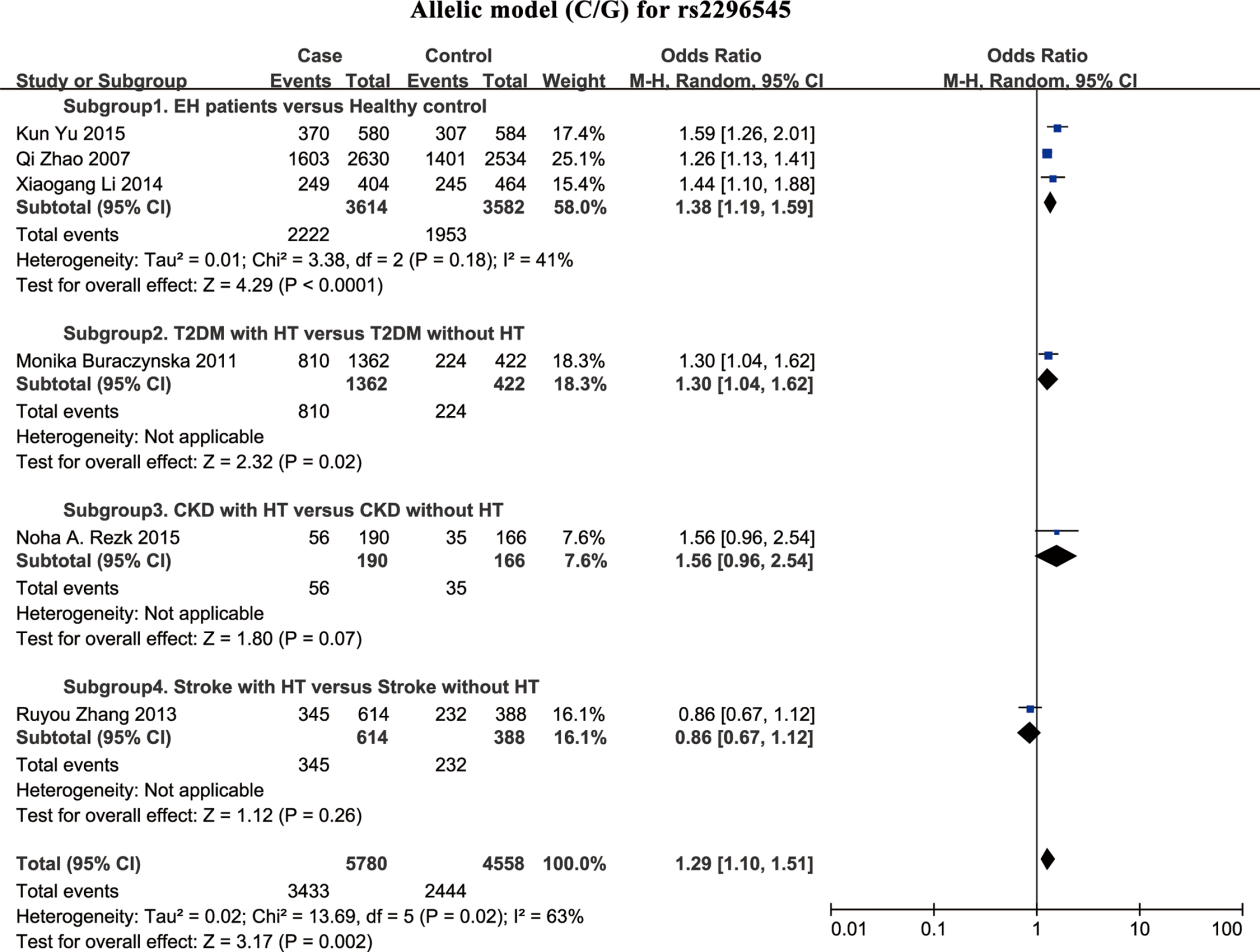
Forest plot of the association between rs2296545 and risk of HT in the allelic model (C/G). Case events indicate hypertensive subjects; Control events indicate normotensive subjects. Note: In Table 2, summary OR for subgroup 1 analysis (allelic model for rs2296545) was been calculated by fixed-effects model (I^2^ = 41%); while, Fig 2 showed this association for combined analysis, other than subgroup 1 analysis, by using random-effects model (I^2^ = 63%). This is the reason for different OR values showed in Table 2 (OR = 1.33) and Fig 2 (OR = 1.38) under subgroup 1 analysis.

**Fig 3:**
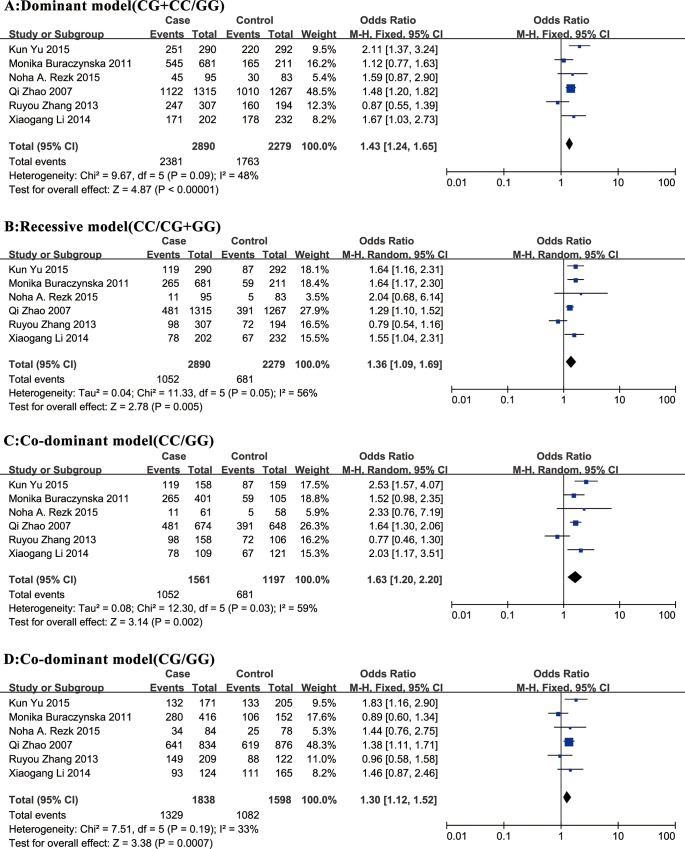
Forest plots of the association between rs2296545 and risk of HT in the dominant, recessive, and codominant models. Case events indicate hypertensive subjects; Control events indicate normotensive subjects.

In this study, for the combined analysis, we observed a statistically significant relationship between SNP rs2296545 and risk of HT in all genetic models (**Table 2**; **Figs 2**and **3**). The C allele of rs2296545 was significantly associated with HT, conferring a pooled OR_RE_ of 1.29 (95% CI = 1.10–1.51), and CC genotype have an approximately 1.63-fold increased risk of HT compared with the rs2296545 GG genotype.

**Table 2:** Summary of pooled ORs and 95%CIs in this meta-analysis.

Variables	N	Dominant model				Recessive model				Co-dominant model								Allelic model			
		OR(95%CI)	P_H_	I^2^	P	OR(95%CI)	P_H_	I^2^	P	OR(95%CI)	P_H_	I^2^	P	OR(95%CI)	P_H_	I^2^	P	OR(95%CI)	P_H_	I^2^	P
**rs2296545**		CG+CC/GG				CC/CG+GG				CC/GG				CG/GG				C/G			
Total	6	**1.43(1.24,1.65)**	**0.09**	**###**	**<0.001**	**1.36(1.09, 1.69)**	**0.05**	**###**	**0.005**	**1.63(1.20,2.20)**	**0.03**	**###**	**0.002**	**1.30(1.12,1.52)**	**0.19**	**###**	**<0.001**	**1.29(1.10,1.51)**	**0.02**	**###**	**0.002**
Subgroup																					
①	3	**1.59(1.34,1.89)**	**0.34**	**8%**	**<0.001**	**1.37(1.20,1.58)**	**0.39**	**0%**	**<0.001**	**1.81(1.49,2.19)**	**0.25**	**###**	**<0.001**	**1.45(1.21,1.75)**	**0.55**	**0%**	**<0.001**	**1.33(1.21,1.46)**	**0.18**	**###**	**<0.001**
②	1	1.12(0.77,1.63)	-	-	-	1.64(1.17,2.30)	-	-	-	1.52(0.98,2.35)	-	-		0.89(0.60,1.34)	-	-		1.30(1.04,1.62)	-	-	
③	1	1.59(0.87,2.90)	-	-	-	2.04(0.68,6.14)	-	-	-	2.33(0.76,7.19)	-	-		1.44(0.76,2.75)	-	-		1.56(0.96,2.54)	-	-	
④	1	0.87(0.55,1.39)	-	-	-	0.79(0.54,1.16)	-	-	-	0.77(0.46,1.30)	-	-		0.96(0.58,1.58)	-	-		0.86(0.67,1.12)	-	-	
Asians	4	**1.47(1.08,2.00)**	**0.05**	**###**	**0.01**	1.28(0.98,1.67)	0.03	67%	0.07	**1.60(1.05,2.45)**	**0.01**	**###**	**0.03**	**1.38(1.17,1.64)**	**0.31**	**###**	**<0.001**	**1.26(1.01,1.56)**	**0.01**	**###**	**0.04**
Caucasians	2	1.24(0.90,1.70)	0.33	0%	0.19	**1.67(1.21,2.31)**	**0.71**	**0%**	**0.002**	**1.61(1.08,2.42)**	**0.49**	**0%**	**0.02**	1.02(0.73,1.43)	0.22	34%	0.90	**1.34(1.10,1.64)**	**0.49**	**0%**	**0.004**
**rs2576178**		AG+GG/AA				GG/AG+AA				GG/AA				AG/AA				G/A			
Total	6	1.22(0.91,1.64)	0	73%	0.18	1.09(0.95,1.26)	0.2	32%	0.20	1.14(0.78,1.66)	0.01	70%	0.50	1.25(0.95,1.63)	0.02	64%	0.11	1.08(0.90,1.30)	0	71%	0.40
Subgroup																					
①	2	**1.44(1.21,1.72)**	**0.23**	**###**	**<0.001**	**1.20(1.02,1.41)**	**0.17**	**###**	**0.03**	1.37(0.86,2.17)	0.11	61%	0.18	**1.41(1.17,1.69)**	**0.42**	**0%**	**<0.001**	1.16(0.94,1.43)	0.13	57%	0.16
②	2	1.36(0.49,3.78)	0.01	85%	0.56	0.94(0.66,1.34)	0.38	0%	0.74	1.26(0.44,3.57)	0.03	79%	0.67	1.39(0.49,3.93)	0.01	83%	0.53	1.09(0.67,1.77)	0.04	76%	0.73
③	1	1.55(1.02,2.36)	-	-		1.06(0.48,2.33)	-	-		1.27(0.57,2.85)	-	-		1.62(1.04,2.52)	-	-		1.35(0.96,1.89)	-	-	
④	1	0.78(0.50,1.21)	-	-		0.79(0.53,1.16)	-	-		0.69(0.42,1.16)	-	-		0.83(0.52,1.34)	-	-		0.83(0.64,1.07)	-	-	
Asians	4	1.28(0.88,1.88)	0.02	71%	0.20	1.13(0.97,1.31)	0.13	48%	0.11	1.24(0.76,2.00)	0.01	74%	0.39	1.30(0.93,1.82)	0.06	59%	0.12	1.10(0.87,1.38)	0.01	72%	0.43
Caucasians	2	1.13(0.62,2.05)	0.02	80%	0.69	0.89(0.61,1.30)	0.62	0%	0.54	0.89(0.58,1.36)	0.3	6%	0.56	1.17(0.63,2.14)	0.03	79%	0.62	1.07(0.70,1.62)	0.04	77%	0.76

N, number of included studies. P_H_, p value of Q-test for the detection of heterogeneity. I^2^, I^2^greater than 50% as evidence of significant heterogeneity. P, p value corresponding to the Z-test for the summary effect estimate (P**<**0.05 considered statistically significant).

Note: I^2^ statistic is been used as major indicator for heterogeneity. If I^2^ > 50% (evidence of heterogeneity), the random-effects model would been used to calculate odds ratios and 95% confidence intervals; otherwise, the fixed-effects model would been used.

① EH patients versus healthy controls

② T2DM patients with HT versus T2DM without HT

③ CKD patients with HT versus CKD without HT

④ stroke patients with HT versus stroke without HT.

In concomitant-disease-specific subgroup analysis, we observed a statistical association between rs2296545 and risk of EH in subgroup 1 with negligible heterogeneity (**Table 2**). This indicates that the C allele of rs2296545 was significantly associated with EH, conferring a pooled OR_RE_ of 1.33 (95% CI = 1.21–1.46). In ethnicity-specific subgroup analysis, we also observed a statistical correlation between rs2296545 and risk of HT in several genetic models (**Table 2**). The C allele in rs2296545 may separately confer 1.26 and 1.34-fold increased risk to HT in Asians and Caucasians.

### Association between rs2576178 polymorphism and HT susceptibility

**Fig 4:**
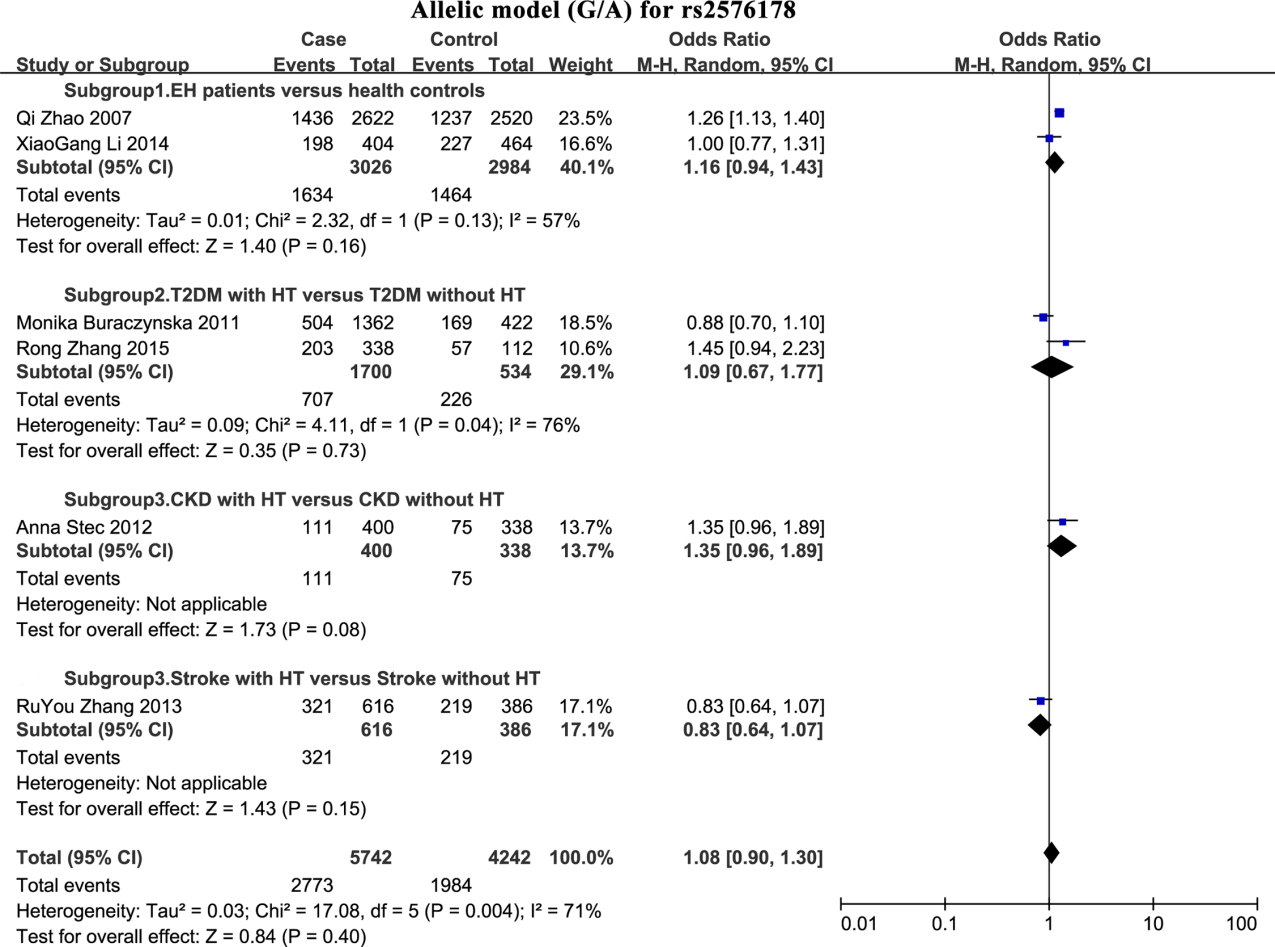
Forest plot of the association between rs2576178 and risk of HT in the allelic model (G/A). Case events indicate hypertensive subjects; Control events indicate normotensive subjects.

We did not observe an association between rs2576178 and risk of HT in the combined analysis (**Fig 4**; **Table 2**): dominant model: OR_RE_ = 1.22, 95% CI = 0.91–1.64; recessive model: OR_FE_ = 1.09, 95% CI = 0.95–1.26; codominant model (GG/AA): OR_RE_ = 1.14, 95% CI = 0.78–1.66; codominant model (AG/AA): OR_RE_ = 1.25, 95% CI = 0.95–1.63; allelic model: OR_RE_ = 1.08, 95% CI = 0.90–1.30.

However, in subgroup 1 analysis (EH patients versus healthy controls), we noted an association between rs2576178 and risk of HT in the dominant, recessive, and codominant genetic models with negligible heterogeneity (**Table 2**): dominant model: OR_FE_ = 1.44, 95% CI = 1.21–1.72, I^2^ = 31%; recessive model: OR_FE_ = 1.20, 95% CI = 1.02–1.41, I^2^ = 47%; codominant model (AG/AA): OR_FE_ = 1.41, 95% CI = 1.17–1.69, I^2^ = 0%. In ethnicity-specific subgroup analysis, no correlation between SNP rs2576178 and HT can be observed.

### Sensitivity analysis and publication bias

Sensitivity analysis was conducted to examine the stability of pooled results by omitting one study at a time and calculating the pooled ORs for the remaining studies. As shown in **Table 3**, a series of pooled ORs with 95% CIs produced repeatedly after the removal of each particular study continuously exceed 1.0. The pooled ORs were similar before and after deletion of each study, indicating the robust stability of the current results. In sensitivity analysis, no association between SNP rs2576178 and risk of HT can be observed.

**Table 3:** Sensitivity analysis for association between rs2296545 and risk of hypertension.

Study omitted (rs2296545)	Dominant model (CG+CC/GG)	Recessive model (CC/CG+GG)	Co-dominant model (CC/GG)	Allelic model (C/G)
	OR (95%CI) / Model	OR (95%CI) / Model	OR (95%CI) / Model	OR (95%CI) / Model
Qi Zhao et al. 2007	1.39(1.01,1.91) / RE	1.39(1.02,1.9) / RE	1.63(1.05,2.54) / RE	1.30(1.04,1.63) / RE
M. Buraczynska et al. 2011	1.49(1.27,1.74) / FE	1.30(1.01,1.68) / RE	1.66(1.13,2.44) / RE	1.29(1.06,1.57) / RE
RuYou Zhang et al. 2013	1.51(1.29,1.75) / FE	1.42(1.25,1.61) / FE	1.77(1.49,2.11) / FE	1.33(1.22,1.45) / FE
XiaoGang Li et al. 2014	1.37(1.06,1.79) / RE	1.33(1.03,1.72) / RE	1.56(1.10,2.23) / RE	1.26(1.05,1.52) / RE
Noha A. Rezk et al. 2015	1.39(1.07,1.80) / RE	1.34(1.07,1.68) / RE	1.59(1.15,2.20) / RE	1.27(1.07,1.50) / RE
Kun Yu et al. 2015	1.36(1.16,1.58) / FE	1.31(1.01,1.68) / RE	1.48(1.08,2.03) / RE	1.23(1.04,1.45) / RE

random-effects model. FE, fixed-effects model.

**Fig 5:**
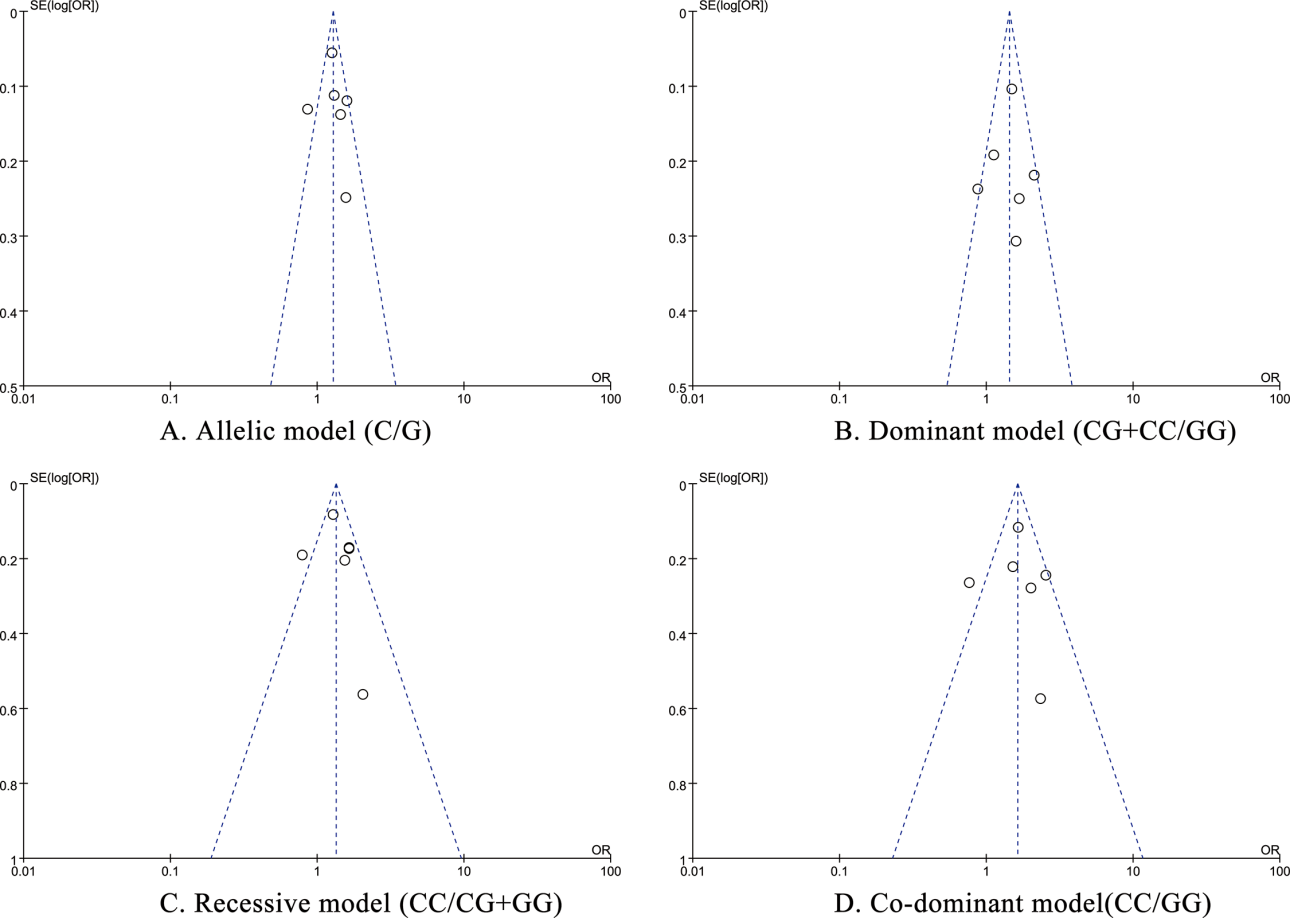
Funnel plot of the association between rs2296545 and risk of HT in the genetic models.

Funnel plots were employed to assess the publication bias of the included studies. The shape of the funnel plots showed no evident publication bias (**Fig 5**).

## Discussion

*RNLS* is located on human chromosome 10 at q23.33 and contains three key structural features, namely, a putative signal peptide at the N terminus, a flavin adenine dinucleotide (FAD)-binding domain, and an amine oxidase domain [27]. Many studies suggest that renalase plays an important role in the pathogenesis of HT. For instance, in animal models, 5/6 nephrectomy rats developed HT and CKD; a dose of recombinant renalase (1.3 mg/kg) administered subcutaneously decreased the SBP and DBP [6]. Similarly, a single dose of renalase or enalapril (1 mg/kg) decreased the SBP and DBP (~7 mmHg) in the stroke-prone spontaneously hypertensive rat [28]. The relationship between *RNLS* polymorphisms and cardiovascular diseases has also been investigated in humans. Zhao et al. [16] first showed that the renalase-coding gene was a novel susceptibility gene for EH in the Chinese population (rs2576178 GG/AA: OR = 1.58, 95% CI = 1.25–2.00; rs2296545 CC/GG: OR = 1.61, 95% CI = 1.26–2.04). Buraczynska et al. [19] determined that the rs2296545 C allele exhibits an increased risk of HT in T2DM patients (OR = 1.29, 95% CI = 1.04–1.61). We also observed an association between *RNLS* gene variation and BP responses to dietary salt intervention in a previous study [29]. However, the results of a few studies are opposite to that obtained in the aforementioned articles. In an urban-based cohort, after adjusting for major cardiovascular risk factors, Fava et al. [22] revealed that the *RNLS* polymorphism is not associated with HT and cardiovascular disease in Caucasians. Rezk et al. [17] showed that the frequencies of SNP rs2296545 CG and CC genotypes and C allele were not significantly different between hypertensive and normotensive CKD patients. The conflicting results indicated that a meta-analysis should be conducted to determine whether SNPs rs2296545 and rs2576178 in the renalase gene were associated with the susceptibility to HT.

In the present meta-analysis, 6 eligible case–control studies involving 2,890 cases and 2,279 controls for SNP rs2296545 and 6 studies involving 2,871 cases and 2,121 controls for SNP rs2576178 were analyzed. By contrast to a previous meta-analysis focusing on the association between rs2296545 and HT [23], we observed a significant association between rs2296545 and risk of HT in all genetic models. In the codominant model (CC/GG), the results indicated that individuals with the rs2296545 CC genotype have an approximately 1.63-fold increased risk of HT compared with the rs2296545 GG genotype. People carrying the C allele in rs2296545 have an approximately 1.29-fold increased risk of HT over those carrying the G allele, and a similar significant relationship was maintained in the dominant and recessive models. Furthermore, in concomitant-disease-specific subgroup analysis, we observed a significant association between rs2296545 and EH in subgroup 1 (all study subjects are Chinese) without heterogeneity. Thus, Chinese people possessing the C allele in rs2296545 have an increased risk of EH over those possessing the G allele. In ethnicity-specific subgroup analysis, an increased susceptibility to HT has been observed in Asians categorized under the dominant, codominant and allelic models with evident heterogeneity and in Caucasians categorized under the recessive, codominant and allelic models without heterogeneity. This finding indicates that the C allele in rs2296545 may confer a higher risk of HT in Asians and Caucasians. However, heterogeneity is a potential problem when interpreting the results of the meta-analysis. In our study, evident heterogeneity exists in the recessive and allelic models for the combined analysis and the Asian-specific subgroup analysis. Through different subgroup analyses, heterogeneity was decreased and eliminated; thus, differences in concomitant disease and ethnicity may be the main reason for between-study heterogeneity. The differences in age and gender distributions among the included studies might also contribute to heterogeneity. Considering the existing heterogeneity, sensitivity analysis was conducted to assess the stability of the pooled effect sizes. No single study markedly altered the pooled effect sizes, and statistical differences remained significant before and after the removal of each study, indicating the high robustness of our results. We observed no evident publication bias in our meta-analysis through the funnel plot. For SNP rs2576178, the pooled effect sizes did not show any correlation between rs2576178 and risk of HT. However, in subgroup 1 analysis, including two studies, we observed a statistically significant correlation between rs2576178 and HT in several genetic models with negligible heterogeneity (dominant model: OR = 1.44, 95% CI = 1.21–1.72, I^2^ = 31%; recessive model: OR = 1.20, 95% CI = 1.02–1.41, I^2^ = 47%; codominant model: OR = 1.41, 95% CI = 1.17–1.69, I^2^ = 0%). This result further supports the conclusion that an association between rs2576178 and risk of HT exists. However, as the studies were limited and heterogeneity does exist, the association should be interpreted with caution.

The underlying mechanism on how the genetic variants in *RNLS* contribute to increased susceptibility to HT is unclear. However, the SNP rs2296545 CC genotype results in a conserved amino acid change at amino acid 37 (glutamic to aspartic acid) within the FAD-binding domain. Compared with Glu37, Asp37 has a significantly lower affinity for NADH, whereas NADH is essential for renalase to perform its enzymatic activity fully [27]. Therefore, this variation plausibly results in functional enzymatic changes that lead to decreased catecholamine degradation, with consequent HT and other cardiovascular diseases. This hypothesis has been partly verified by Rezk et al. [17], who reported that CKD patients with the rs2296545 CC genotype showed significantly higher epinephrine level as compared with the CG and GG genotypes. However, whether renalase is an amine oxidase or not is currently the subject of debate among researchers. Aliverti [30] and Moran et al. [31] failed to demonstrate the catalytic activity of renalase, which contradicts the results reported by Xu et al. [5]. Therefore, further in-depth basic research should be conducted to verify that hypothesis.

Recently, Shi et al. [23] reported that the renalase rs2296545 polymorphism was not associated with risk of hypertension in a meta-analysis. However, this study only included genotyped cases and controls (503 cases and 490 controls) from stage 1 of the Zhao et al. [16] study, and did not account for 814 genotype cases and 779 genotyped controls from stage 2 of that study, which was included in the present meta-analysis. This may be one of reason for their negative meta-analysis finding for rs2296545. Moreover, it is unclear whether Shi et al. included Chinese studies. In the present meta-analysis, more studies were included and supplementary analyses, including subgroup and sensitivity analyses, were conducted. Meanwhile, another *RNLS* SNP rs2576178 was also analyzed. The quality of the included studies was assessed by using the modified NOS, and all control groups in this meta-analysis achieved the HWE.

Despite the aforementioned strengths of the present study, several limitations must be acknowledged. Firstly, in concomitant-disease-specific subgroup analysis, particularly the last two subgroups, the number of included studies is relatively few to analyze the association between SNPs and risk of HT in CKD and stroke patients fully. Secondly, we failed to obtain complete original data from the studies included, so it was hard to make adjustments for gender, or some other factors. Finally, although we found an association between both SNPs and the risk of EH in subgroup 1 analysis, we cannot be sure that the remaining hypertensive patients (subgroup 2–4) are EH patients as the comorbid conditions of diabetes and CKD, which can cause secondary HT, were present in them or not. Thus, we can conclude that *RNLS* polymorphisms are correlated with HT, not EH.

In conclusion, the present study revealed that the SNP rs2296545 in *RNLS* is associated with the increased risk of HT, whereas the SNP rs2576178 may not be associated with HT. These findings should be interpreted with caution because of the existing heterogeneity and limited studies. Well-designed studies with a larger sample size and more ethnic groups are required to validate the risk identified in the current meta-analysis.

## Supporting Information

S1 File Checklists for meta-analysis on genetic association studies. (ZIP)

S2 File Modified Newcastle-Ottawa Scale for case-control studies of genetic association. (DOCX)

S3 File Excluded studies and reasons for exclusion. (ZIP)

S1 Table Supplementary information about included studies in the meta-analysis. (XLSX)

S2 Table Quality assessment of studies included in the meta-analysis using a modified Newcastle-Ottawa Scale. (DOCX)
