# Supplementary material for: Association of Renalase SNPs rs2296545 and rs2576178 with the Risk of Hypertension: A Meta-Analysis
Source: PLoS One. 2016 Jul 19;11(7):e0158880. doi: 10.1371/journal.pone.0158880 (PMC4951046; doi:10.1371/journal.pone.0158880)
Supplement: S3 File — (ZIP) [file pone.0158880.s003.zip › 11 excluded records/2x Cristiano Fava et al. 2012.pdf]

Fava et al. BMC Medical Genetics 2012, 13:57
http://www.biomedcentral.com/1471-2350/13/57

BMC Medical Genetics

RESEARCH ARTICLE Open Access

The *Renalase* Asp37Glu polymorphism is not associated with hypertension and cardiovascular events in an urban-based prospective cohort: the Malmö Diet and cancer study

Cristiano Fava[1,2,4*], Martina Montagnana[1,3†], Elisa Danese[1,3†], Marketa Sjögren[1], Peter Almgren[1], Gunnar Engström[1], Bo Hedblad[1], Gian Cesare Guidi[3], Pietro Minuz[2] and Olle Melander[1]

Abstract

Background: Renalase (gene name *RNLS*), a recently discovered enzyme with monoamine oxidase activity, is implicated in the degradation of catecholamines. Recent studies delineate a possible role of this enzyme in blood pressure (BP) maintenance and cardiac protection and two single nucleotide polymorphisms, *RNLS* rs2576178 A > G and rs2296545 C > G have been associated with hypertension. The latter SNP leads to a non synonymous Asp to Glu substitution deleting a flavin adenine dinucleotide (FAD) binding site with possible impaired functionality. We tested the hypothesis that these polymorphisms could affect BP levels, hypertension prevalence, and risk of incident cardiovascular events in middle-aged Swedes.

Methods: The polymorphisms were genotyped in 5696 participants of the population-based Cardiovascular Cohort of the "Malmö Diet and Cancer" (MDC-CC). The incidence of cardiovascular events (coronary events [n = 408], strokes [n = 330], heart failure [n = 190] and atrial fibrillation/flutter [n = 406]) was monitored for an average of approximately 15 years of follow-up.

Results: Both before and after adjustment for sex, age and BMI the polymorphisms did not show any effect on BP level and hypertension prevalence. Before and after adjustment for major cardiovascular risk factors, the hazard ratio for cardiac and cerebrovascular events was not significantly different in carriers of different genotypes. A significant interaction was found between the rs2296545 C > G and age with respect to BP/hypertension.

Conclusions: Our data do not support a major role for these *RNLS* polymorphisms in determining BP level and incident events at population level. The positive interaction with age suggest that the effect of the rs2296545 C > G polymorphism, if any, could vary between different ages.

Keywords: Renalase, Blood pressure, Cardiovascular events, Hypertension, Polymorphisms

Background

Renalase (gene name *RNLS*) is a recently discovered enzyme with monoamine oxidase activity implicated in the degradation of catecholamines and probably other currently unknown substrates [1,2].

* Correspondence: cristiano.fava@med.lu.se
†Equal contributors
[1]Department of Clinical Sciences, Lund University, University Hospital of Malmö, Malmo, Sweden
[2]Department of Medicine and Department of Life and Reproduction Sciences, University Hospital of Verona, Verona, Italy
Full list of author information is available at the end of the article

The human *RNLS* maps on chromosome 10 at q23.33 and has 10 exons which leads to the production of at least four alternatively spliced isoforms. The most highly expressed isoform (renalase1) is 342 amino acids long, and is the predominant human renalase protein detected in plasma, kidney, heart, skeletal muscle and liver. It contains a signal peptide, a flavin adenine dinucleotide (FAD)-binding region, at the extreme amino terminus, and an amine oxidase domain.

Renalase activity is markedly augmented by an increase in plasma catecholamines, suggesting that

BioMed Central
© 2012 Fava et al.; licensee BioMed Central Ltd. This is an Open Access article distributed under the terms of the Creative Commons Attribution License (http://creativecommons.org/licenses/by/2.0), which permits unrestricted use, distribution, and reproduction in any medium, provided the original work is properly cited.

renalase plays a role in the minute to-minute regulation of blood pressure [2,3].

Data obtained by different experimental approaches indicate that renalase deficiency, even in the absence of significant kidney disease, is associated with hypertension. Renalase gene expression, downregulated in the rat using small interfering RNAs, did not affect renal function but increased BP both at rest and during an exogenous catecholamines burst [1]. In renalase knockout (KO) mouse, despite normal renal function, kidney histology, and plasma aldosterone levels, heart rate and BP increased both during activity and at rest [1].

Plasma levels are markedly reduced in patients with end stage renal disease (ESRD), suggesting that the kidney is the predominant organ which secretes renalase and regulates circulating levels. It is noteworthy that subtotal nephrectomy is associated with left ventricular hypertrophy, and interestingly this is paralleled by a significant decrease in heart hRenalase1 levels in neonatal and adult rats [3,4].

Indeed, it was demonstrated that recombinant renalase had a protective effect on the myocardium during ischemia and decreased the myocardial infarct size by nearly one half [5]. Finally in two rat models of renal hypoperfusion, unilateral renal artery stenosis and infarction-induced heart failure, it was shown a diminished renalase production and a concomitant increase of circulating norepinephrine suggesting that impaired synthesis of renalase by the kidney may represent a potential mechanism underlying circulating norepinephrine accumulation in heart failure [5,6].

In humans, Zhao et al.[7] tested for association of the *RNLS* gene with essential hypertension by examining several single nucleotide polymorphisms (SNPs) of *RNLS* in more than 2,000 individuals from the International Collaborative Study of Cardiovascular Disease in Asia (InterASIA in China).

Two SNPs (rs2576178 A > G and rs2296545 C > G) were associated with essential hypertension.

Interestingly the latter SNP leads to a conservative amino acid change (glutamic to aspartic acid at amino acid 37) which, may weaken FAD binding and affect the function of renalase. More recently, the same SNP has been associated with cardiovascular phenotypes such as cardiac hypertrophy, dysfunction and ischemia in a cohort of 590 Caucasian individuals with stable coronary artery disease (CAD) [8].

The aim of our study is to test if the *RNLS* rs2576178 A > G and rs2296545 C > G (Arg37Glu) polymorphisms is implicated in hypertension development and incidence of coronary and cerebrovascular events, acute heart failure and atrial fibrillation in a large population-based cohort study: the Malmö Diet and Cancer (MDC) study – cardiovascular arm (CVA) including more than 5,000 middle-aged subjects.

Methods

All study participants had given written informed consent. The procedures were in accordance with the institutional guidelines. The Ethics Committee of the Medical Faculty of Lund University approved the study.

Subjects

MDC-CVA

Between 1991 and 1996, women aged 45 to 73 years and men aged 46 to 73 years, with residency in Malmö (approximately 250,000 habitants), Sweden, were invited by mail and by newspaper advertisement to participate in the MDC [9,10]. In all, 28,449 participated out of an eligible population of 74,000. The participants were asked to complete a self-administered questionnaire at home, which included items on lifestyle factors, medication, previous and current diseases. Blood pressure (BP) along with other cardiovascular risk factors were measured in a random subsample referred to as the MDC-CVA (n = 6,103) [11]. Successfully extracted genomic DNA was available from 5,763 MDC-CVA participants.

Blood pressure

We performed the study of BP as a continuous variable before and after adjustment of measured BP values (see below) and as a dichotomized tract (hypertension vs. normotension). BP was measured once, after 10 minutes of rest in the supine position, by specially trained nurses on the right brachial artery using a mercury sphygmomanometer. The systolic BP was defined by 'phase I' and the diastolic BP defined by 'phase V' Korotkoff sounds.

Definition of Hypertension and Blood Pressure adjustment

Hypertension was defined as being on antihypertensive treatment or having systolic BP/diastolic BP equal or greater than 140/90 mmHg according to current diagnostic criteria [12] and normotension as having systolic BP/diastolic BP less than 140/90 mmHg.

Blood pressure adjustment

To overcome the possibility that a biased selection might result from selecting only individuals who were free of antihypertensive treatments, we conducted an analysis adjusting the systolic BP and diastolic BP of hypertensive individuals that were taking antihypertensive drugs at the time of investigation by two methods recently reviewed by Cui and colleagues [13].

Fixed addition

Based on the known average treatment effects, fixed increments of 10 mmHg systolic BP and 5 mmHg diastolic BP was added to treated pressures.

Stepped addition
To account for the number of drugs, stepped increments of 8/4, 14/10, 20/16, 26/22 mmHg were added to the measured systolic BP/diastolic BP of treated individuals taking one, two, and three drug classes, respectively.

Anthropometric, behavioral and laboratory parameters
Waist circumference (in cm) was measured with the patient standing, at the umbilicus level. The BMI was calculated as the ratio of the weight in kilograms to the square of the height in meters (kg/m2). Smoking habits of individuals were elicited by a self-administered questionnaire and categorized into 'non smokers' (including former smokers) and 'current smokers'.

After an overnight fast, blood samples were drawn for the determination of serum lipids and whole blood glucose. Samples were analyzed by standard methods at the Department of Clinical Chemistry, Malmö University Hospital, which is attached to a recurrent standardized system [11].

Diabetes was defined in the MDC-CVA as either a fasting whole blood glucose ≥ 6.1 mmol/L or current therapy with antidiabetic drugs or a self-reported history of physicians diagnosis of diabetes.

Cystatin C was measured using a particle-enhanced immuno-nephelometric assay (N Latex Cystatin, Siemens Diagnostics), as previously described in the same cohort [14], and estimated glomerular filtration rate (eGFRcystatin C) was derived by the following formula. eGFRcystatin C = −4.32 + 80.35 x 1/cystatin C [15].

Follow-up, definition of end points
All subjects were followed from the baseline examination until the first cardiovascular event, death or 31 December 2006. Mean follow-up time spanned from 14.5 ± 3.9 years for atrial fibrillation to 15.4 ± 3.7 for acute heart failure episodes. Cardiac disease end points were ascertained by linkage of Swedish personal identification numbers to the national Swedish registers (Swedish Hospital Discharge Register, Swedish Cause of Death Register) maintained by the Swedish National Board of Health and Welfare. High case validity in these registers has been previously found for heart failure [16], myocardial infarction [17], and atrial fibrillation [17,18]. Heart failure was ascertained from the Swedish Hospital Discharge Register using diagnosis codes 427.00, 427.10, and 428.99 for International Classification of Diseases-8th Revision (ICD-8), 428 for the 9th Revision (ICD-9), and I50 and I11.0 for the 10th Revision (ICD-10) as primary diagnosis as in previous studies [16].

Atrial fibrillation was defined using diagnosis codes 427.92 (ICD-8), 427D (ICD-9), and I48 (ICD- 10) as in previous studies [18]. Coronary events was defined using diagnosis codes 410 (ICD-8 and −9) and I21 (ICD-10) or as death from ischemic heart disease defined using diagnosis codes 412 and 414 (ICD-8 and −9) or I22-I23 and I25 (ICD-10) as in previous studies [17]. Stroke was defined using diagnosis codes 434,436 (ICD-9) [10]. Transient ischemic attacks (TIA) were, by definition excluded. All incident coronary events, stroke, heart failure and atrial fibrillation events used for the analysis refers to first hospitalized events.

Genotyping
DNA was extracted from frozen granulocyte or buffy-coat samples using QIAamp-96 spin blood kits (QIAGEN, Stockholm, Sweden) at the DNA extraction facility supported by SWEGENE. The *RNLS* rs2576178 A > G and rs2296545 C > G, were determined by end-point fluorescent measurements [19] using TaqMan MGB probes custom synthesized by Applied Biosystems: [wild type/mutant], respectively

VIC/FAM-AGAGGAAAGGTTGCCCGTGGTATCGC [C/T]GGTAAATTCTTCTTCCCACTTTCAA

VIC/FAM-TGGCGTTTAGACAACCCACCTGAGTC [C/G]TCAGCCTTGTCCCACACAGCAAGGT

Statistics
Continuous variables are presented as the mean ± SD. All data, except for the power analysis, were analyzed with SPSS statistical software (version 18.0; SPSS Inc. Chicago, Illinois, USA). Power calculation was performed using the Power and Sample Size calculator version 2.1.31 (Vanderbilt University Medical Center, Nashville, USA). Frequency differences and deviation from Hardy- Weinberg equilibrium were analyzed by chi-square test. Significance of differences in continuous variables was tested by analysis of variance followed by Tukey's test and *t*-test. Multiple linear and logistic regression analyses were used in the multivariate models with either BP traits or hypertension status as dependent variables and genotype, age, sex, BMI, and the interaction variables (computed by multiplying the genotype with age, sex and BMI respectively) as independent variables.

Kaplan-Meier curves and log-rank tests compared cumulative incidence of strokes, coronary events, acute heart failure and atrial fibrillation/flutter episodes in carriers of different genotypes. Age-, sex- and traditional risk factor (diabetes, hypertension, current smoking, hypercholesterolemia defined as LDL-cholesterol ≥ 4.15 mmol/L mmol/l or current therapy with anti-lipidemic drugs, previous cardiovascular event) adjusted Cox proportional-hazard models were used to study the relationships between the polymorphisms and time (in years) to first cardiovascular events. The fit of the proportional hazards model was confirmed by plotting the cardiovascular incidence rates over time. Hazard ratios

(HR) and 95% confidence interval (CI) were calculated. For variables with skewed distributions, log-normalized values were used in the analysis. All tests were two-sided and P values less than 0.05 were considered statistically significant.

Results

The clinical characteristics of individuals included in the study in the MDC-CVA are summarized in Table 1. The genotyping success rate was 98.5% (5,666/5,752) for *RNLS* rs2576178 A > G and 97.5% (5,608/5,752) for the rs2296545 C > G. We found respectively for the rs2576178, 53.6% AA homozygotes, 38.5% AG-heterozygotes and 7.9% GG-homozygotes and for the rs2296545, 29.7% CC-homozygotes, 49.4% CG-heterozygotes and 21.0% GG-homozygotes.

Genotype distributions did not deviate from Hardy–Weinberg equilibrium for both SNPs (predicted heterozygosity 0.385, observed heterozygosity 0.396; P = 0.05 for *RNLS* rs2576178 A > G and predicted heterozygosity 0.494, observed heterozygosity 0.496; P = 0.70 for rs2296545 C > G).

Power analysis in MDC-CVA

Regarding the *RNLS* rs2576178 A > G and rs2296545 C > G respectively this study has 80% power to detect an odds ratio (OR) for hypertension greater than 1.31/1.20 according to an autosomal recessive genetic model (AA&AG vs. GG and CC&CG vs. GG), greater than 1.17/1.19 according to a dominant (AA vs. AG&GG and CC vs. CG&GG) genetic model and greater than 1.13/1.12 according to an additive genetic model.

Regarding continuous BP variables, analyzing the *RNLS* rs2576178 A > G SNP, if an autosomal dominant/recessive/additive genetic model is assumed, the study has 80% power to detect a difference of respectively >1.41/>2.61/>1.12 mmHg in systolic BP and >0.70/>1.29/>0.55 mmHg in diastolic BP between subjects carrying different genotypes. Analyzing the *RNLS* rs2296545 C > G SNP, if an autosomal dominant/recessive/additive genetic model is assumed, the study has 80% power to detect a difference of respectively >1.55/>1.74/>1.01 mmHg in systolic BP and >0.77/>0.87/>0.50 mmHg in diastolic BP between subjects carrying different genotypes.

Blood pressure

Crude BP data and hypertension prevalence according to genotype at MDC-CVA are presented in Table 2. After adjustment for covariates and antihypertensive therapy, no significant differences were found between carriers of different genotypes under all the tested genetic models (Table 3).

Analyzing hypertension prevalence as a dichotomous trait we did not find any difference in carriers of different *RNLS* rs2576178 A > G genotype [OR: 0.987 (95% CI: 0.902-1.080, P = 0.77) for the additive model; 0.986 (0.798-1.218, P = 0.90) for the autosomal recessive model and 0.983 (0.877- 1.102, P = 0.98) for the autosomal dominant model] and rs2296545 C > G genotype [OR: 0.988 (95% CI: 0.911-1.072, P = 0.77) for the

Table 1 Anthropometric and metabolic features of the whole sample and divided by people with either previous cardiovascular event, diabetes mellitus and chronic kidney disease (CKD)

Characteristics	All (n = 5696)	With previous CV event or diabetes or CKD (n = 570)	No previous CV event or diabetes or CKD (n = 5126)
**Gender, male (%)**	41.7	57.9	40.0
**Age, years**	57.47±5.94	57.21±5.93	59.75±5.51
**Body mass index, kg/m^2** †	25.84±3.98	25.56±3.81	28.37±4.56
**Systolic blood pressure, mmHg**	141.18±18.95	140.40±18.75	148.21±19.34
**Diastolic blood pressure, mmHg**	86.94±9.45	86.63±9.38	89.78±9.60
**Hypertension, %**	63.3	81.9	61.2
**Antihypertensive therapy, %**	16.2	40.4	13.6
**Obesity, %** †	13.6	31.3	11.6
**Diabetes, %** ‡	8.6	80.5	0
**Current smoking, %** §	27.6	25.6	27.8
**Hypercholesterolemia, %** ††	49.7	51.5	49.5
**eGFR <90 ml/min/1.73 m^2, %** ‡‡	24.0	31.9	18.9
**eGFR <60 ml/min/1.73 m^2, %** ‡‡	0.7	7.7	0
**Alcohol consumption, gr/day**§§	10.19±12.11	11.69±15.71	10.03±11.62
**History of CV events**	2.1	20.5	0

CKD, chronic kidney disease evaluated as a eGFR <60 ml/min/1.73 m^2.
Information missing in † 7 subjects, ‡ 549 subjects, § 322 subjects, ††668 subjects, ‡‡ 873 subjects, §§726 subjects.

Table 2 Crude blood pressure values and hypertension prevalence according to genotypes in MDC-CVA

	RNLS rs2576178 AA	*RNLS* rs2576178 AG	*RNLS* rs2576178 GG	*RNLS* rs2576178 A-carriers	*RNLS* rs2576178 G-carriers
	n = 3,035	n = 2,183	n = 448	n = 5,218	n = 2,631
**SBP, mmHg**	141.63±18.96	140.42±18.91	141.77±18.89	141.12±18.95	140.65±18.91
**DBP, mmHg**	87.01±9.49	86.75±9.39	87.38±9.28	86.89±9.45	87.01±9.49
**Hypertension, %**	63.8	62.8	63.4	63.4	62.9
**Antihypertensive treatment, %**	16.0	16.8	15.8	16.3	16.6
	RNLS	***RNLS***	***RNLS***	***RNLS***	***RNLS***
	**rs2296545 CC**	**rs2296545 CG**	**rs2296545 GG**	**rs2296545 C-carriers**	**rs2296545 G-carriers**
	n = 1,663	n = 2,768	n = 1,177	n = 4431	n = 3,945
**SBP, mmHg**	141.33±18.79	141.08±18.82	141.58±19.53	141.17±18.81	141.23±19.04
**DBP, mmHg**	87.08±9.32	86.94±9.41	86.95±9.62	86.99±9.38	86.95±9.47
**Hypertension, %**	63.5	63.5	63.2	63.5	63.4
**Antihypertensive treatment, %**	17.4	15.9	15.9	16.5	15.9

SBP, systolic blood pressure; DBP, Diastolic blood pressure.

additive genetic model; 0.971 (0.843-1.118, P = 0.68) for the autosomal recessive model and 0.994 (0.877-1.127, P = 0.93) for the autosomal dominant model].

Also the comparison between patients with a previous medical diagnosis of hypertension (n = 1829) and individuals with BP below 130/85 mmHg (n = 1,136) showed

Table 3 Association between *RNLS rs2576178* A > G and *rs2296545 C* > *G* polymorphisms and BP according to different genetic models

	RNLS rs2576178					
	Genetic model					
**Type of blood pressure adjustment for AHT**	**Additive**	**p-value**	**Autosomal recessive**	**p-value**	**Autosomal dominant**	**p-value**
**Crude (non adjusted)**						
SBP (mmHg)	−0.311 (0.365)	0.39	0.552 (0.863)	0.52	−0.669 (0.467)	0.15
DBP (mmHg)	0.074 (0.187)	0.69	0.491 (0.442)	0.27	−0.023 (0.239)	0.92
**Fixed addition**						
SBP (mmHg)	−0.254 (0.386)	0.51	0.492 (0.913)	0.59	−0.558 (0.494)	0.26
DBP (mmHg)	0.102 (0.198)	0.60	0.461 (0.467)	0.32	0.032 (0.253)	0.90
**Stepped addition**						
SBP (mmHg)	−0.297 (0.387)	0.44	0.477 (0.915)	0.60	−0.625 (0.495)	0.21
DBP (mmHg)	0.065 (0.202)	0.75	0.429 (0.478)	0.37	−0.019 (0.259)	0.94
	RNLS rs2296545					
**Type of blood pressure adjustment for AHT**	**Additive**	**p-value**	**Autosomal recessive**	**p-value**	**Autosomal dominant**	**p-value**
**Crude (non adjusted)**						
SBP (mmHg)	0.075 (0.332)	0.82	0.336 (0.575)	0.56	−0.087 (0.513)	0.87
DBP (mmHg)	−0.118 (0.170)	0.49	−0.143 (0.294)	0.63	−0.168 (0.262)	0.52
Fixed addition						
SBP (mmHg)	−0.022 (0.351)	0.95	0.249 (0.609)	0.68	−0.251 (0.543)	0.64
DBP (mmHg)	−0.167 (0.179)	0.35	−0.187 (0.311)	0.55	−0.250 (0.277)	0.37
**Stepped addition**						
SBP (mmHg)	0.033 (0.352)	0.93	0.336 (0.610	0.58	−0.188 (0.544)	0.73
DBP (mmHg)	−0.124 (0.183)	0.50	−0.099 (0.318)	0.76	−0.217 (0.284)	0.44

Adjustment for age, sex, BMI.

no significant difference in genotype frequencies ($P >$ 0.05 for all). The same analysis were repeated after exclusion of subjects with previous cardiovascular events (n = 117), diabetes mellitus (n = 443) and CKD (n = 38) and after adjusting for other metabolic covariates but the results did not change significantly (see online Additional file 1: Table S1 and Additional file 1: S2).

Interaction with demographic variables and stratified analysis

In linear and logistic regression no interaction of the *RNLS* rs2576178 A > G polymorphisms with sex, age or BMI were evident (see Additional file 1: Table S3 in the supplementary material). Anyhow, for the *RNLS* rs2296545 C > G a significant interaction with age was evident both in the additive and autosomal dominant genetic model. Stratifying the population in quartiles of age, the *RNLS* rs2296545 C > G conferred protection vs. systolic BP (β ± SE: -1.351 ± 0.617, P = 0.029), diastolic BP (β ± SE: - 1.014 ± 0.347, P = 0.004) and hypertension (OR: 0.835 (95% CI: 0.717-0.972, P = 0.02) in the lowest quartile of age (i.e. <52.3 years, additive model). Whereas a trend toward higher systolic BP (β ± SE: 2.237 ± 1.156, P = 0.053), diastolic BP (β ± SE: 1.470 ± 0.551, P = 0.008) and hypertension (OR: 1.248 (95% CI: 0.956-1.628, P = 0.10) was observed in the highest quartile of age (i.e. >62.6 years, autosomal dominant model). Hypothesizing that this interaction could be due to a decrease in renal function with aging we performed an exploratory analysis, within a subsample where eGFR cystatin was available (n = 4,823). No association between the rs2296545 C > G polymorphism and hypertension-related traits was detectable (see Additional file 1: Table S4).

Cardiac and cerebrovascular events

During an average follow-up of more than 14 years we retrieved 408 coronary events, 330 strokes, 190 heart failure and 406 atrial fibrillation/flutter episodes.

Kaplan-Meier curves, by log-rank test, did not show any difference in cardiac and cerebrovascular episodes in carriers of different *RNLS* rs2576178 A > G and *RNLS* rs2296545 C > G genotypes (Figure 1 and 2). In Cox regression analysis, when adjusting for age and sex, both the *RNLS* rs2576178 A > G and *RNLS* rs2296545 C > G were not associated with coronary events, stroke, acute heart failure or atrial fibrillation/flutter (see Table 4).

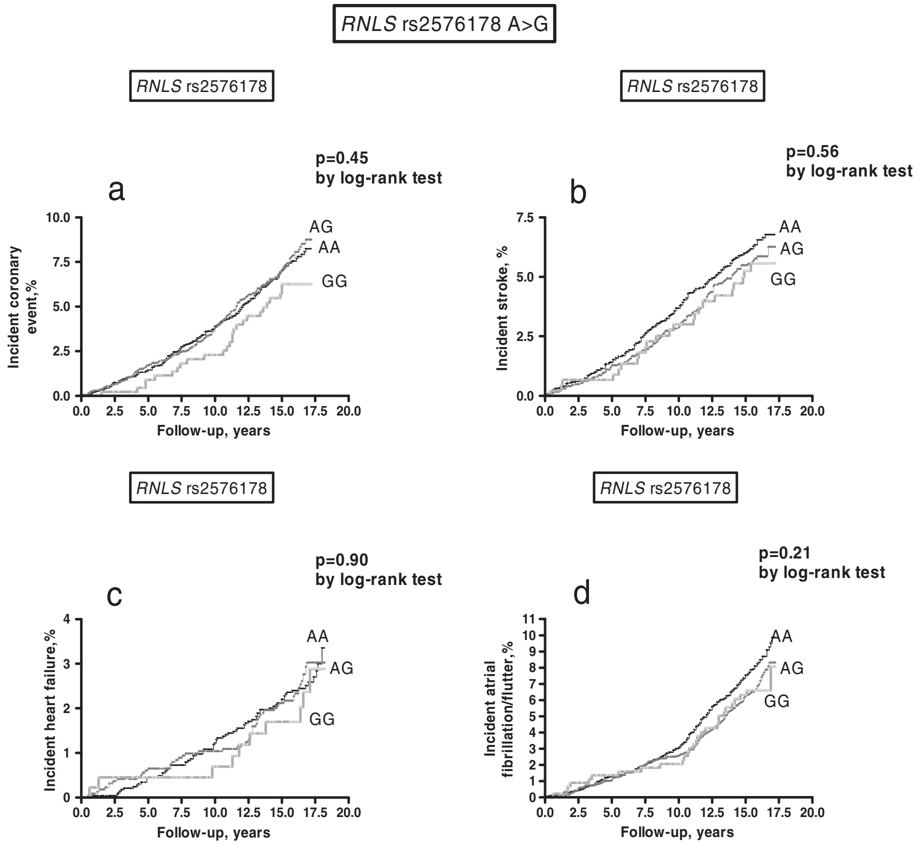

Figure 1 Incident coronary events (a), stroke (b), heart failure events (c), atrial fibrillation/flutter events (d), in carriers of different *RNLS* rs2576178 *A* > *G* genotypes.

When further adjustment for traditional cardiovascular risk factors (including smoking habit, previous cardiovascular events, hypertension, diabetes mellitus, total cholesterol) was included in the models the results did not change substantially but a borderline significant difference was noticed for incident coronary events in *RNLS* rs2296545 GG-homozygotes as compared to C-carriers and for incident atrial fibrillation/flutter in *RNLS* rs2576178 G-carriers with respect to AA-homozygotes (Table 4).

Discussion

Evidences are accumulating indicating a pivotal role of the renalase enzyme in different cardiovascular diseases through its modulating effect on plasma catecholamines [20]. Thus, in different animal models decreased levels of renalase were associated with elevated BP, ischemic heart disease, heart failure. Also first genetic studies in humans focusing especially on the putatively functional polymorphism *RNLS* rs2296545 showed an association with hypertension, cardiac hypertrophy and inducible ischemia [7,8].

Our study, at variance with previous reports, found no association with hypertension related traits in a well-powered sample of middle-aged men and women participating in the MDC-CVA. There are different possible explanations for this discrepancy. Previous reports refer to population collected with different design, selection criteria, clinical characteristics and genetic background. Association of the *RNLS* rs2576178 and rs2296545 polymorphisms with hypertension were first assessed in 1,317 hypertensive cases and 1,269 normotensive controls from China participating in the InterASIA study. The association was evaluated in two stages and both the SNPs resulted associated with hypertension with marginally significant p-value. It is worth noting that the minor allele frequency in the Chinese study is very different for the rs2576178 (0.48) with respect to our study (0.27) [7]. In another study, Farzaneh-Far and colleagues recruited 590 subjects with a previous diagnosis of stable CAD and all the

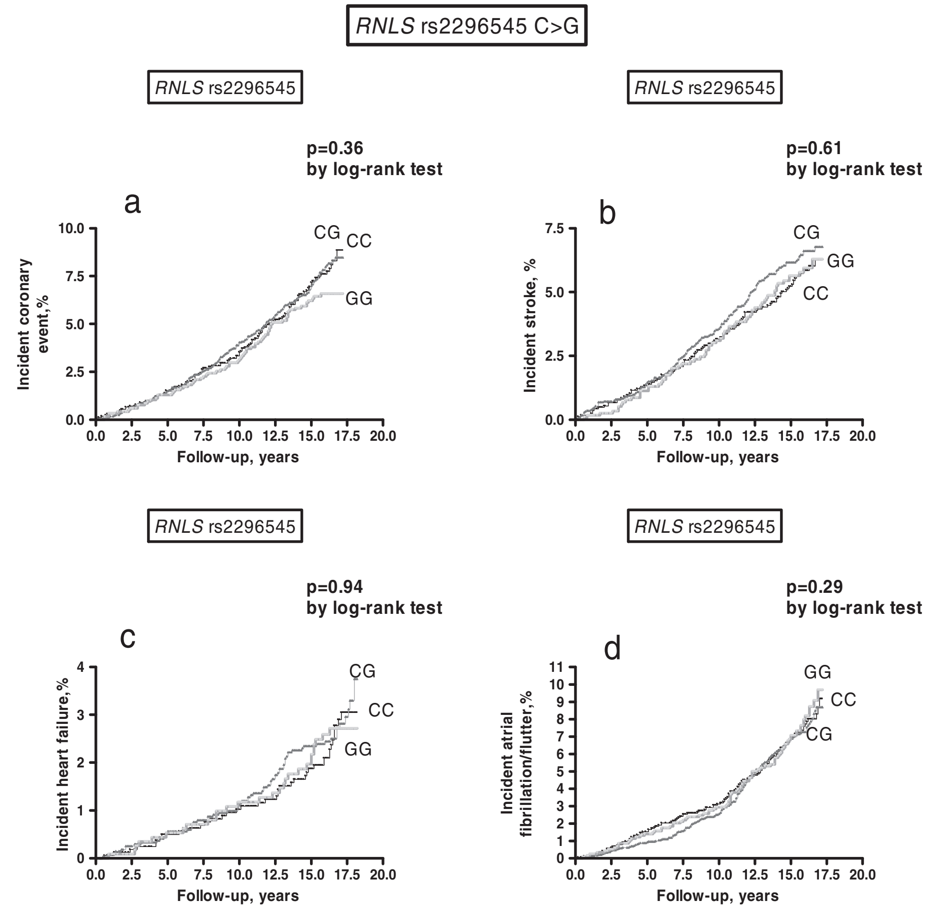

Figure 2 Incident coronary events (a), stroke (b), heart failure events (c), atrial fibrillation/flutter events (d), in carriers of different *RNLS* rs2296545 C > *G* genotypes.

Table 4 Hazard ratio and 95% CI for different cardiovascular events conferred by the *RNLS rs2576178* A > G and *rs2296545 C > G* polymorphisms tested by COX regression according to different genetic model in MDC-CVA

	Genetic model					
	**Additive**	**p-value**	**Autosomal recessive**	**p-value**	**Autosomal dominant**	**p-value**
			***RNLS* rs2576178**			
**Coronary events***	0.963 (0.826-1.123)	0.63	0.791 (0.532-1.177)	0.25	1.001 (0.824-1.217)	0.99
**Coronary events†**	0.977 (0.820-1.163)	0.79	0.798 (0.507-1.256)	0.33	1.021 (0.819-1.275)	0.85
**Stroke***	0.909 (0.764-1.082)	0.28	0.867 (0.567-1.324)	0.51	0.893 (0.717-1.111)	0.31
**Stroke†**	0.866 (0.710-1.056)	0.16	0.663 (0.387-1.137)	0.13	0.881 (0.689-1.127)	0.31
**Heart failure events***	1.014 (0.808-1.273)	0.90	0.887 (0.504-1.660)	0.68	1.059 (0.791-1.417)	0.70
**Heart failure events†**	1.033 (0.800-1.335)	0.80	0.801 (0.408-1.575)	0.52	1.117 (0.805-1.550)	0.51
**Atrial fibrillation/flutter events***	0.874 (0.746-1.024)	0.09	0.852 (0.581-1.225)	0.41	0.843 (0.692-1.027)	0.09
**Atrial fibrillation/flutter events†**	0.843 (0.706-1.006)	0.06	0.859 (0.562-1.313)	0.48	0.795 (0.638-0.990)	0.04
			***RNLS* rs2296545**			
**Coronary events***	0.933 (0.812-1.073)	0.32	0.843 (0.653-1.089)	0.19	0.964 (0.780-1.193)	0.74
**Coronary events†**	0.888 (0.757-1.042)	0.15	0.727 (0.536-0.987)	0.04	0.949 (0.745-1.209)	0.67
**Stroke***	1.040 (0.892-1.213)	0.62	0.982 (0.749-1.287)	0.89	1.115 (0.876-1.421)	0.38
**Stroke†**	1.128 (0.948-1.342)	0.18	1.115 (0.831-1.497)	0.47	1.229 (0.928-1.626)	0.15
**Heart failure events***	0.971 (0.789-1.195)	0.78	0.936 (0.647-1.354)	0.72	0.981 (0.714-1.348)	0.98
**Heart failure events†**	0.976 (0.771-1.236)	0.84	0.896 (0.643-1.472)	0.90	0.965 (0.674-1.381)	0.85
**Atrial fibrillation/flutter events***	1.030 (0.897-1.184)	0.67	1.079 (0.851-1.369)	0.53	1.011 (0.815-1.253)	0.92
**Atrial fibrillation/flutter events†**	1.051 (0.901-1.226)	0.53	1.113 (0.856-1.446)	0.42	1.033 (0.814-1.312	0.79

*Adjustment for age, sex; † adjustment for hypertension, diabetes mellitus, smoking habit, previous CV event, hypercholesterolemia.

outcomes associated with the *RNLS* Asp37Glu were referred to echocardiographic measurements of hypertrophy and inducible ischemia that were not evaluated in our study [8]. BP measure in our sample is based on a single occasion in supine position; on the other hand, the large sample size should have minimized the potential problem linked to low accuracy. Moreover, previous epidemiological studies have shown an association of supine BP with hard cardiovascular end-points even when BP was measured only in a single occasion [21,22]. Also hypertension definition based on a cut-off could be prone to misclassification and we used different criteria with respect to the InterASIA study where hypertension was defined as BP > 150/100 mmHg or use of antihypertensive drugs and normotension as BP <140/90 mmHg. On the other hand when we used the same criteria as those used in the InterASIA study or compared only people with diagnosed hypertension to subjects with BP < 130/85 mmHg we obtained no different results.

The positive interaction we found between the rs2296545 (Asp37Glu) and age with respect to hypertension, suggests that the Glu37 variant should be protective at lower ages and deleterious at advancing ages. This finding is difficult to explain and despite having found several times interaction with sex in the same population sample, this is first time we detected such kind of interaction [23-25]. We can only hypothesize that it could be linked either to aging itself or to another unmeasured factor strictly correlated with aging. I.e. both a decrease production of the enzyme with aging could render more evident the effect of the Asp37Glu or the interaction with other metabolic enzymes or the natural decrease in renal function could be hypothesized as plausible explanations. Our exploratory analysis argues against the last hypothesis about kidney function but the statistical power we have in the subgroup with impaired renal function is quite low.

Whereas further ad hoc study try to clarify this issue, we invite to consider carefully the age of investigated people when this particular polymorphism is tested for association with hypertension.

During a follow-up of approximately 15 years also "hard" end points, such as myocardial infarction, strokes, atrial arrhythmic events and heart failure were not associated with these polymorphisms in our population. Even if we found a borderline significant results suggesting as a protective role of these SNPs about coronary events and atrial fibrillation episodes, our findings would go exactly in the opposite direction respect to what could be expected on the basis of previous results about hypertension in Japanese people and cardiac hypertrophy in CAD patients with the putatively "deleterious" minor

alleles conferring a protection respect to these cardiovascular events. Indeed, the accordance we have about results on BP and cardiovascular end-points suggest a negligible effect of this polymorphism, at least in our population.

We focused our attention especially on the *RNLS* rs2296545 which results in an Asp to Glu substitution at codon 37, which is located near a deduced FAD-binding site of the renalase protein.

Since renalase is critically dependent on FAD for oxidase activity, the polymorphism could be functional. For the *RNLS* rs2576178 there are no functional data in vitro but the polymorphism is in the 5' flanking region of the gene and could be involved in transcriptional regulation. However, we cannot exclude that other SNPs in the same gene in variable grade of linkage disequilibrium with our SNPs could be implicated in BP/hypertension or other cardiovascular related outcomes.

Strengths of our study are the large sample size and the population-based-design with prospective assessment of cardiovascular end-points; whereas limitations refers especially to the measurement of BP at a single-time point. Indeed, our results are not generalizable to populations with different genetic background.

Conclusion

In a Swedish urban-based cohort, including more than 5,000 subjects, we found no evidence of association between two common *RNLS* SNPs, hypertension and cardiovascular events suggesting that at population level these polymorphisms are of negligible importance, at least in Caucasian.

Perspectives

Further studies are needed to elucidate if conflicting results respect to previous studies about the effect of *RNLS* on hypertension and BP related phenotypes are linked to selection criteria or different genetic background of the analyzed populations. About other cardiologic outcomes, it will be important to test if at risk population, such as people affected by CAD or with impaired renal function could be more prone to the putative deleterious effect of *RNLS* polymorphisms. Moreover, a possible effect of age interacting with the Asp37Glu polymorphism should be considered when selecting newer populations where to study this gene.

Additional file

Additional file 1: Table S1. Association between the *RNLS* polymorphisms and BP according to different genetic model after excluding subjects with previous CV events, diabetes mellitus and CKD (n=4209). **Table S2.** Association between the *RNLS* polymorphisms and BP according to different genetic model after further adjustment for glucose, triglycerides, total cholesterol, high density lipoprotein cholesterol, serum cystatin C, smoking and drinking status (n=4209). **Table S3.** Beta coefficient and SE for interaction terms (either sex or age) with RNLS *rs2576178* and *rs2296545* polymorphisms and BP-related traits according to different mode of inheritance. **Table S4.** Association between the *RNLS rs2296545* polymorphism and BP according to different genetic model in subjects with impaired kidney function.

Competing interest

The authors declare that they have no competing interests.

Authors' contribution

CF participated in the study design, carried out the molecular genetic studies, participated in the sequence alignment, performed statistical analyses and drafted the manuscript. MM carried out the molecular genetic studies, participated in the sequence alignment and drafted the manuscript. ED carried out the molecular genetic studies, participated in the sequence alignment and drafted the manuscript. MS carried out the molecular genetic studies and participated in the sequence alignment. PA performed statistical analyses and helped to draft the manuscript. GE, BH, GCG, PM and OM participated in study coordination and helped to draft the manuscript. All authors read and approved the final manuscript.

Author details

[1]Department of Clinical Sciences, Lund University, University Hospital of Malmö, Malmo, Sweden. [2]Department of Medicine and Department of Life and Reproduction Sciences, University Hospital of Verona, Verona, Italy. [3]Department of Life and Reproduction Sciences, University Hospital of Verona, Verona, Italy. [4]Department of Medicine, Division of Internal Medicine C, Piazzale LA Scuro 10, 37134, Verona, Italy.

Received: 7 March 2012 Accepted: 10 July 2012
Published: 19 July 2012

References

1. Desir GV: Regulation of blood pressure and cardiovascular function by renalase. *Kidney Int* 2009, **76**:366–370.
2. Xu J, Li G, Wang P, Velazquez H, Yao X, Li Y, Wu Y, Peixoto A, Crowley S, Desir GV: Renalase is a novel, soluble monoamine oxidase that regulates cardiac function and blood pressure. *J Clin Invest* 2005, **115**:1275–1280.
3. Li G, Xu J, Wang P, Velazquez H, Li Y, Wu Y, Desir GV: Catecholamines regulate the activity, secretion, and synthesis of renalase. *Circulation* 2008, **117**:1277–1282.
4. Ghosh SS, Krieg RJ, Sica DA, Wang R, Fakhry I, Gehr T: Cardiac hypertrophy in neonatal nephrectomized rats: the role of the sympathetic nervous system. *Pediatr Nephrol* 2009, **24**:367–377.
5. Wu Y, Xu J, Velazquez H, Wang P, Li G, Liu D, Sampaio-Maia B, Quelhas-Santos J, Russell K, Russell R, Flavell RA, Pestana M, Giordano F, Desir GV: Renalase deficiency aggravates ischemic myocardial damage. *Kidney Int* 2011, **79**:853–860.
6. Gu R, Lu W, Xie J, Bai J, Xu B: Renalase deficiency in heart failure model of rats-a potential mechanism underlying circulating norepinephrine accumulation. *PLoS One* 2011, **6**:e14633.
7. Zhao Q, Fan Z, He J, Chen S, Li H, Zhang P, Wang L, Hu D, Huang J, Qiang B, Gu D: Renalase gene is a novel susceptibility gene for essential hypertension: a two-stage association study in northern Han Chinese population. *J Mol Med* 2007, **85**:877–885.
8. Farzaneh-Far R, Desir GV, Na B, Schiller NB, Whooley MA: A functional polymorphism in renalase (Glu37Asp) is associated with cardiac hypertrophy, dysfunction, and ischemia: data from the heart and soul study. *PLoS One* 2010, **5**:e13496.
9. Berglund G, Elmstahl S, Janzon L, Larsson SA: The Malmo Diet and Cancer Study. Design and feasibility. *J Intern Med* 1993, **233**:45–51.
10. Zia E, Hedblad B, Pessah-Rasmussen H, Berglund G, Janzon L, Engstrom G: Blood pressure in relation to the incidence of cerebral infarction and intracerebral hemorrhage. Hypertensive hemorrhage: debated nomenclature is still relevant. *Stroke* 2007, **38**:2681–2685.
11. Nilsson PM, Engstrom G, Hedblad B: The metabolic syndrome and incidence of cardiovascular disease in non-diabetic subjects–a population-based study comparing three different definitions. *Diabet Med* 2007, **24**:464–472.

12. Mancia G, De Backer G, Dominiczak A, Cifkova R, Fagard R, Germano G, Grassi G, Heagerty AM, Kjeldsen SE, Laurent S, Narkiewicz K, Ruilope L, Rynkiewicz A, Schmieder RE, Boudier HA, Zanchetti A, Vahanian A, Camm J, De Caterina R, Dean V, Dickstein K, Filippatos G, Funck-Brentano C, Hellemans I, Kristensen SD, McGregor K, Sechtem U, Silber S, Tendera M, Widimsky P, Zamorano JL, Erdine S, Kiowski W, Agabiti-Rosei E, Ambrosioni E, Lindholm LH, Viigimaa M, Adamopoulos S, Agabiti-Rosei E, Ambrosioni E, Bertomeu V, Clement D, Erdine S, Farsang C, Gaita D, Lip G, Mallion JM, Manolis AJ, Nilsson PM, O'Brien E, Ponikowski P, Redon J, Ruschitzka F, Tamargo J, van Zwieten P, Waeber B, Williams B: **Management of Arterial Hypertension of the European Society of Hypertension; European Society of Cardiology. 2007 Guidelines for the Management of Arterial Hypertension: The Task Force for the Management of Arterial Hypertension of the European Society of Hypertension (ESH) and of the European Society of Cardiology (ESC).** *J Hypertens* 2007, **25:**1105–1187.
13. Cui JS, Hopper JL, Harrap SB: **Antihypertensive treatments obscure familial contributions to blood pressure variation.** *Hypertension* 2003, **41:**207–210.
14. Smith JG, Newton-Cheh C, Almgren P, Struck J, Morgenthaler NG, Bergmann A, Platonov PG, Hedblad B, Engström G, Wang TJ, Melander O: **Assessment of conventional cardiovascular risk factors and multiple biomarkers for the prediction of incident heart failure and atrial fibrillation.** *J Am Coll Cardiol* 2010, **56:**1712–1719.
15. Hoek FJ, Kemperman FA, Krediet RT: **A comparison between cystatin C, plasma creatinine and the Cockcroft and Gault formula for the estimation of glomerular filtration rate.** *Nephrol Dial Transplant* 2003, **18:**2024–2031.
16. Ingelsson E, Arnlov J, Sundstrom J, Lind L: **The validity of a diagnosis of heart failure in a hospital discharge register.** *Eur J Heart Fail* 2005, **7:**787–791.
17. Hammar N, Alfredsson L, Rosen M, Spetz CL, Kahan T, Ysberg AS: **A national record linkage to study acute myocardial infarction incidence and case fatality in Sweden.** *Int J Epidemiol* 2001, **30**(Suppl 1):S30–S34.
18. Smith JG, Platonov PG, Hedblad B, Engstrom G, Melander O: **Atrial fibrillation in the Malmo Diet and Cancer study: a study of occurrence, risk factors and diagnostic validity.** *Eur J Epidemiol* 2010, **25:**95–102.
19. Livak KJ: **Allelic discrimination using fluorogenic probes and the 5' nuclease assay.** *Genet Anal* 1999, **14:**143–149.
20. Desir GV: **Renalase is a novel renal hormone that regulates cardiovascular function.** *J Am Soc Hypertens* 2007, **1:**99–103.
21. Puddu PE, Menotti A, Tolonen H, Nedeljkovic S, Kafatos AG: **Determinants of 40-year all-cause mortality in the European cohorts of the Seven Countries Study.** *Eur J Epidemiol* 2011, **26:**595–608.
22. Menotti A, Lanti M, Kafatos A, Nissinen A, Dontas A, Nedeljkovic S, Kromhout D, Seven Countries Study: **The role of a baseline casual blood pressure measurement and of blood pressure changes in middle age in prediction of cardiovascular and all-cause mortality occurring late in life: a cross-cultural comparison among the European cohorts of the Seven Countries Study.** *J Hypertens* 2004, **22:**1683–1690.
23. Fava C, Montagnana M, Almgren P, Rosberg L, Guidi GC, Berglund G, Melander O: **Association between adducin-1 G460W variant and blood pressure in Swedes is dependent on interaction with body mass index and gender.** *Am J Hypertens* 2007, **20:**981–989.
24. Fava C, Montagnana M, Almgren P, Rosberg L, Guidi GC, Berglund G, Melander O: **The functional variant of the CLC-Kb channel T481S is not associated with blood pressure or hypertension in Swedes.** *J Hypertens* 2007, **25:**111–116.
25. Fava C, Montagnana M, Almgren P, Rosberg L, Lippi G, Hedblad B, Engström G, Berglund G, Minuz P, Melander O: **The V433M variant of the CYP4F2 is associated with ischemic stroke in male Swedes beyond its effect on blood pressure.** *Hypertension* 2008, **52:**373–380.

doi:10.1186/1471-2350-13-57
Cite this article as: Fava *et al.*: The *Renalase* Asp37Glu polymorphism is not associated with hypertension and cardiovascular events in an urban-based prospective cohort: the Malmö Diet and cancer study. *BMC Medical Genetics* 2012 **13**:57.

Submit your next manuscript to BioMed Central and take full advantage of:

- **Convenient online submission**
- **Thorough peer review**
- **No space constraints or color figure charges**
- **Immediate publication on acceptance**
- **Inclusion in PubMed, CAS, Scopus and Google Scholar**
- **Research which is freely available for redistribution**

Submit your manuscript at
www.biomedcentral.com/submit

BioMed Central
