# Supplementary material for: Association of Renalase SNPs rs2296545 and rs2576178 with the Risk of Hypertension: A Meta-Analysis
Source: PLoS One. 2016 Jul 19;11(7):e0158880. doi: 10.1371/journal.pone.0158880 (PMC4951046; doi:10.1371/journal.pone.0158880)
Supplement: S3 File — (ZIP) [file pone.0158880.s003.zip › 11 excluded records/2x Master thesis of LuHong Li 2013.pdf]

中图分类号 R541.3　　　　　　　　　　学校代码 10533
UDC　610　　　　　　　　　　　　　　密级　公开

硕士学位论文

肾胺酶基因多态性与高血压及合并冠心病的相关性研究

Renalase Gene Polymorphisms in patients with hypertension and cornary heart disease

作 者 姓 名：　李禄洪
学 科 专 业：　临床医学
研 究 方 向：　心血管内科
学院（系、所）：湘雅三医院
指 导 教 师：　蒋卫红 教授

论文答辩日期 2013. 5. 23　　答辩委员会主席 [illegible]

中南大学
二零一三年五月

Y2426363

原创性声明

本人声明，所呈交的学位论文是本人在导师指导下进行的研究工作及取得的研究成果。尽我所知，除了论文中特别加以标注和致谢的地方外，论文中不包含其他人已经发表或撰写过的研究成果，也不包含为获得中南大学或其他单位的学位或证书而使用过的材料。与我共同工作的同志对本研究所作的贡献均已在论文中作了明确的说明。

作者签名：[illegible]　　日期：2013 年 5 月 24 日

学位论文版权使用授权书

本人了解中南大学有关保留、使用学位论文的规定，即：学校有权保留学位论文并根据国家或湖南省有关部门规定送交学位论文，允许学位论文被查阅和借阅；学校可以公布学位论文的全部或部分内容，可以采用复印、缩印或其它手段保存学位论文。同时授权中国科学技术信息研究所将本学位论文收录到《中国学位论文全文数据库》，并通过网络向社会公众提供信息服务。

作者签名：[illegible]　　导师签名 [illegible]　　日期：2013 年 5 月 24 日

肾胺酶基因多态性与高血压及合并冠心病的相关性研究

摘要：

目的：通过对高血压合并冠心病人群进行肾胺酶基因多态性分析，探讨肾胺酶不同基因型及等位基因频率与高血压合并冠心病的相关性，筛查出其可能相关的易感位点，为高血压患者罹患冠心病风险的评估预警提供遗传学依据。

方法：选取2012年9月至2013年2月期间入住中南大学湘雅三医院心内科的患者，其中高血压合并冠心病组191例，单纯高血压组202例，健康对照组232例。记录各组人员临床一般资料及生化指标；通过NCBI及HapMap基因组数据库筛选标签SNP并进行引物设计及限制性内切酶的选择；对各组病例血样进行DNA的提取、鉴定与稀释，利用限制性片段长度多态性聚合酶链反应的方法对本研究入选的三个标签SNP位点（rs10887800、rs2576178、rs2296545）进行检测，记录各组个体基因型。利用Haploview4.2软件进行各组基因型、等位基因频率、单体型、连锁不平衡模式分析及哈迪温伯格平衡检验；通过非条件性logistici回归分析计算OR值进行风险评估。

结果：

1. SNP单位点分析

1.1 rs2576178的A等位基因在高血压合并冠心病组的等位基因频率高于单纯高血压组，p=0.001，OR=1.625，95% CI 为【1.221~2.160】；AA基因型携带者在高血压合并冠心病组的比例显著高于单纯高血压组，P=0.001。

1.2 rs2296545的C等位基因在单纯高血压组的等位基因频率高于健康对照组，P=0.009，OR=1.436,95% CI 为【1.095~1.883】；CC基因型携带者在单纯高血压组的比例高于健康对照组，p=0.037。

1.3 rs10887800在三组间及两两比较均无具有统计学意义的差异。

2. 单体型分析

总体样本中共有六种单体型符合P＞0.05；两两组间比较共有三种单体型差异具有统计学意义：单体型G-A-C在高血压合并冠心病的比例明显大于单纯高血压组，P=0.013，OR=1.565，95%CI 为[1.097~2.232]；单体型G-G-C在单纯高血压组的比例高于健康对照

组，P=0.012，OR=1.745，95%CI 为 [1.127~2.703]；单体型 A-A-G 在高血压合并冠心病组的比例明显高于健康对照组，P=0.001，OR=2.322，95%CI 为[1.425~3.783]。

结论：

1. rs2576178 的 A 等位基因可能是高血压患者罹患冠心病的易感因子，其对应的 G 等位基因可能是其保护因子。AA 型携带的高血压患者易罹患冠心病。

2. rs2296545 的 C 等位基因可能是高血压的易感因子，其对应的 G 等位基因可能是保护因子。CC 型携带者易患高血压。

本文共计图 11 幅、表 25 个，参考文献 18 篇。

关键词：肾胺酶；基因多态性；高血压；冠心病

分类号：R541.3

Renalase Gene Polymorphisms in patients with hypertension and coronary heart disease

ABSTRACT:

Objective:The aim of our study was to investigate the involvement of renalase gene polymorphisms in coronary heart disease in hypertension patients.To explore the correlation between renalase gene and hypertension with coronary heart disease by gene polymorphisms analysis.As filtering out predisposing gene locus,we will provide genetic basis for risk assessment of coronary heart disease in hypertension.

Methods:191 patients with hypertension and CHD,202 patinents with hypertension and 232 controls were recruited.Clinical and biochemical information was recorded.Primer design and the choice of the Restriction endonuclease was through NCBI and HapMap database.Renalase genotypes were determined by PCR-RFLP method.We analyzed all of the data including genotypes,allele frequen -cies,haolotypes,linkage disequilibrium and Hardy-Weinberg equilibrium.

Results:1.analysis of SNP

1.1 rs2576178 A allele frequencies of hypertension and CHD group was higher than hypertension group,p=0.001, OR=1.625, 95% CI 为【1.221~ 2.160】.AA genotype frequencies was similar with A allele frequencies,P=0.001.

1.2 rs2296545 C allele frequencies in hypertension group was higher than control group,P=0.009, OR=1.436,95% CI 为【1.095~1.883】; CC genotype frequencies was similar with C allele frequencies,P=0.037.

1.3 There was no association with all of groups about rs10887800.

2.analysis of Haplotype

Six Haplotypes match P>0.05.Haplotype G-A-C in coronary heart disease and hypertension group was higher than hypertension group,P=0.013, OR=1.565, 95%CI[1.097~2.232];Haplotype G-G-C in hypertension group was higher than control group,P=0.012, OR=1.745, 95%CI [1.127~2.703];Haplotype A-A-G in coronary heart disease and hypertension group was higher than control group,P=0.001, OR=2.322,

95%CI[1.425~3.783].

Conclusions:1.rs2576178 A allele may be a susceptibility factor for hypertensive patients suffering from coronary heart diease,and its corresponding G allele may be a protective factor.AA genotype susceptibe to coronary heart disease in hypertension.

2.rs2296545 C allele may be a susceptibility factor for hypertension,and its corresponding G allele may be a protective factor.CC genotype susceptible to hypertension.

11picture, 25 tables, 18 references

Keywords: renalase ; gene polymorphism ; hypertension ; coronary heart disease

Classification: R541.3

目 录

原创性声明......I
摘要......II
目 录......VI
符号说明......VII
1 前 言......1
1.1 高血压及冠心病现状......1
1.2 renalase 概述......2
1.3 renalase 基因多态性研究现状......2
1.4 本次研究假说及目的......3
2 研究设计及方法......4
2.1 对象人群的选择......4
2.2 SNP 的筛选......4
2.3 一般资料及生化指标......4
2.4 DNA 提取及基因型鉴定......5
2.4.1 主要试剂......5
2.4.2 试剂配制......5
2.4.3 试验步骤......5
2.5 统计学处理......8
3 结果......9
3.1 研究人群的临床特征......9
3.2 各 SNP 基因型鉴定图......9
3.3 SNP 的 HWE 检验及连锁不平衡分析......12
3.4 Renalase 基因三组样本统计学分析......13
3.5 renalase 基因单体型分析......19
3.6 Renalase 各基因型与临床一般资料及生化指标的比较分析......21
4 讨论......23
5 结 论......25
参考文献......26
综 述......28
攻读硕士学位期间主要研究成果......36
致 谢......37

符号说明

缩写	英文全称	中文名称
SNP	Single Nucleotide Polymorphisms	单核苷酸多态性
EDTA	Ethylene Diamine Tetraacetic Acid	乙二胺四乙酸
EB	ethidium bromide	溴化乙锭
OD	photodensity	光密度
PCR	Polymerase Chain Reaction	聚合酶链式反应
BMI	body mass index	体重指数
GLU	glucose	血糖
TC	total cholesterol	总胆固醇
TG	triglycerides	甘油三酯
HDL-C	high density lipoprotein cholesterol	高密度脂蛋白胆固醇
LDL-C	Low density lipoprotein cholesterol	低密度脂蛋白胆固醇
Cr	Creatinine	肌酐
HWE	Hardy-Weinberg equilibrium	哈迪温伯格平衡
LD	linkage disequilibrium	连锁不平衡

1 前 言

1.1 高血压及冠心病现状

高血压作为最常见的的慢性病及心脑血管疾病的危险因素，能对人体内心、脑、肾等靶器官造成损害，不仅威胁大众群体的生命健康，而且给公共卫生事业带来巨大的压力及挑战。我国历经 50 年，共进行了四次大规模高血压患病率调查，前两次采用了不同的高血压诊断标准，1991 年开始的第三次大规模抽查开始选用目前沿用的的标准，即血压≥140/90mmHg 及两周内服用降压药者，该年调查共筛查了 30 个省、市、自治区的共 95 万人，结果显示，当时我国 15 周岁以上人群高血压粗患病率为 11.88%。2002 年选取了共 27 人进行抽样调查，结果显示我们 18 周岁及 15 周岁以上人群高血压粗患病率分别为 18.8%、17.7%，粗略估算当时全国高血压患者约为 1.6 亿左右。2010 年我国成人高血压患病率增加至 20%。2013 年世界高血压联盟主席刘力生教授报道，我国目前高血压患者约为 3.3 亿。高血压的高患病率结合我国人口众多的实际情况，如此多的高血压患病人群及其可能进展的各种并发症，将对我国卫生事业造成巨大的负担。如何更好的预防及控制高血压是医疗工作者的一项重要使命。血压的调控机制及其随之而来的各种并发症的发生发展一直是相当复杂的进程[1-3]。对于高血压的致病机制及易感基因的相关研究，能从功能学及遗传学的角度更好的对高血压进行防控。

冠心病是动脉粥样硬化导致器官病变的最常见类型，也是危害严重的疾病。随着人民生活水平的提高，其患病率近年呈上升趋势。其分型包括心绞痛型、心肌梗死型、无症状性心肌缺血型、缺血性心肌病型及猝死型。在个体症状方面，特征性胸痛是提示冠心病的一大因素。然而在临床实践中，冠心病起病隐匿，部分患者平素无任何前驱症状即发生心梗或造成猝死，其高致残致死率对群众生命健康造成极大威胁。目前对冠心病的预防多为对危险因素的控制。通过对冠心病的相关致病基因进行筛查，从分子生物学角度对冠心病发病风险进行综合分析，能对冠心病的总体防控起到积极的作用。

高血压作为冠心病的一大危险因素，二者联系紧密。在患有高血压的基础上，何种类型的人群更易罹患冠心病，一直不甚清楚。目前虽然对冠心病的发生发展有了基本认识，但冠心病起病隐匿，进展很快，传统的诊断手段如心电图很难早期发现；运动平板试验有部分老年人不能耐受；冠状动脉造影是目前冠心病诊断和冠状动脉病变程度评价的“金指标”，但也存在检测手段复杂、有创、费用高等

不足，而基因检测手段能够通过相关致病基因的基因型鉴定，从遗传学的角度更好更早期地评估患者罹患疾病的风险。

肾胺酶是近年来新发现的一种生物大分子，被认为可能在心血管调节方面存在重要作用。本研究将从基因多态性的角度分析肾胺酶与高血压合并冠心病之间的关联。

1.2 renalase 概述

肾脏是血压调节的重要器官。2005 年，Xu 等[4]首次阐述了 renalase 的历史及其可能存在的潜在功能，为这个 FAD 依赖的单胺氧化酶揭开了神秘的面纱。他们发现这种由肾脏分泌，血液中循环的酶能够降解血液组织中的儿茶酚胺，可能在交感兴奋性及血压的控制中起到相应的作用[4]。Wu 等[5]发现 renalase 基因敲除的大鼠与野生大鼠相较，肾功能方面并无二致，但是 renalase 基因敲除的大鼠普遍存在血压高、心动过速的情况，并且该类型鼠体内相较野生大鼠有着更高的儿茶酚胺水平。他们通过建立心肌缺血再灌注模型的方法，发现 renalase 基因缺失的大鼠在相同条件下心肌缺血损害较之野生大鼠更为严重，并且注射重组 renalase 能够明显改善心肌损伤[5]，提示 renalase 可能和心肌缺血有关。

透过现有的基因组数据库信息（NCBI 数据库及 HapMap 数据库）可知，renalase 基因位于 10 号染色体的 q23.33。它包含 7 个外显子及存在 2 处转录变异，能够编码 4 种不同的剪接异构体（hRenalase 1-4）[6]，并且这些剪接异构体具有组织特异性。目前只有 hRenalase1 号亚型能在人体血液组织中检测出来，这同时表明分布于其他组织的另外三种亚型可能存在不同的功能。

1.3 renalase 基因多态性研究现状

目前关于 renalase 基因多态性研究有限，且主要围绕高血压展开。基于 renalase 基因多态性研究的 SNP 位点选择多位于基因潜在的功能区域（如位于外显子区域的 SNP）。改变氨基酸序列的位点，很可能直接影响编码蛋白质的结构或功能的稳定性。基于这些原则，目前的单核苷酸多态性研究主要集中在以下几个位点：

1.rs2296545 该位点通过对氨基酸序列的改变，导致第 37 位氨基酸的转变，由天冬氨酸变异为谷氨酸。Zhao 等[7]研究发现，该位点和高血压存在关联。Buraczynska 等[8]研究发现，rs2296545 位点与高血压合并糖尿病相关。

2.rs2576178 该位点位于 renalase 基因的 5'侧翼，可能影响基因的功能。学者研究发现，该位点亦可能和高血压发病相关[4]。Stec 等[9]发现终末期肾病的高

血压发病与该位点相关。

3.rs10887800 该位点位于 renalase 基因外显子与内含子的交界区。研究发现此位点与终末期肾病相关[9]。另有学者发现肾胺酶与动脉粥样硬化存在可能的关联，该研究发现 renalase 基因 rs10887800 位点可能与高血压病人发生脑卒中相关[10]。

1.4 本次研究假说及目的

Renalase 作为目前未被充分认知的生物大分子，其潜在的心血管方面的功能使其成为本次研究选择的目的基因。该基因被证实与高血压、高血压脑梗的发生发展可能相关。冠心病作为日渐影响人类健康的一大因素，其与高血压、动脉粥样硬化紧密相关。高血压作为冠心病的一大危险因素，其易感基因可能与冠心病相关；冠心病与脑卒中可同为动脉粥样硬化在不同靶器官长期作用所致的病症，亦具有一定程度的关联。因此提出假说，与高血压、高血压脑梗具有一定相关性的 renalase 基因多态性可能与高血压合并冠心病有关。本研究探讨的便是 renalase 基因多态性与高血压合并冠心病的相关性，为高血压患者罹患冠心病风险的评估预警提供遗传学依据。

2 研究设计及方法

2.1 对象人群的选择

本研究所收选的人群为2012年9月至2013年2月于中南大学湘雅三医院心内科住院病人以及于我院健康体检中心体检人员。根据研究需要将拟收选病人分为三组，分别为单纯高血压组、高血压合并冠心病组及对照组。高血压病例需满足2010年高血压防治指南修订版的诊断标准，高血压合并冠心病病例参照冠脉造影结果或既往史存在心梗等标准。各组成员均为中国南方汉族人群，以排除种群差异性导致的假阳性可能。

本研究排除以下对象：继发性高血压患者；其他心脏疾病患者(心肌炎、心肌病、心包炎、心脏瓣膜病等)；糖尿病及慢性肾脏病患者；外周血管病患者；自身免疫性疾病及肿瘤患者等。

2.2 SNP 的筛选

一个基因有数十个SNP位点，并非每个位点都有意义。能够高效性代表其他SNP位点进行复杂性遗传研究的SNP称为标签SNP。通过NCBI及HapMap等基因组数据库筛选CHB（中国-北京-汉族）中的renalase数据。使用Haploview4.2软件进行筛选与本实验相关的标签SNP，本研究使用以下标准来挑选适合的SNP：1）该位点SNP次要等位基因频率不小于0.05；2）优先选择影响因子高的阳性发现。本研究最终选取rs10887800、rs2576178、rs2296545三个SNP进行基因型、等位基因测定，以及单体型分析，以明确renalase与中国湖南长沙地区高血压及高血压合并冠心病的相关性。

2.3 一般资料及生化指标

查阅研究期间入选患者住院病历共393份，记录患者性别、年龄、高血压病程、入院时血压状况、吸烟饮酒情况及BMI等一般临床资料；记录患者入院检测的血钠水平、肾功能情况（肌酐水平）、血糖、血脂（总胆固醇、甘油三酯、高密度脂蛋白胆固醇、低密度脂蛋白胆固醇）等生化指标。记录健康人群体检报告共232份，方式同前所述。

所有入选研究的人群均由有较强技能的本科室护士使用紫头真空采血管（EDTA螯合抗凝）采自肘静脉，标本于2500转/分钟离心20分钟，采集血细

胞后置于-70 摄氏度冷冻保存。

2.4 DNA 提取及基因型鉴定

2.4.1 主要试剂

全血基因组 DNA 提取试剂盒	美国普洛麦格公司
异丙醇	长沙明瑞化工有限公司
无水乙醇	长沙明瑞化工有限公司
琼脂糖	美国西格玛奥德里奇公司
EB	美国 Amresco 公司
2xEasy Taq PCR super Mix	南京百斯凯科技有限公司
引物	南京百斯凯科技有限公司
限制性内切酶	南京百斯凯科技有限公司

主要仪器设备

Gene Amp PCR System 9700	美国 ABI 公司
低温离心机	北京时代北利有限公司
DYY-10C 型电泳仪	北京六一仪器厂
电热恒温水浴箱	上海新苗医疗器械制造有限公司
GD8000 型紫外凝胶成像系统	美国 UVP 公司
分光光度计	美国 Beckman 公司
电子天平	上海梅特勒-托利多仪器有限公司
微量移液器	艾本德有限公司
电热恒温鼓风干燥器	上海新苗医疗器械制造有限公司

2.4.2 试剂配制

1）EB：将 500mg 溴化乙锭加入到 50ml 双蒸水中，搅拌充分至完全溶解，制备好的 EB 储液浓度为 10mg/ml，避光于室温保存。

2）1xTAE 电泳缓冲液：选取 1L 烧杯，使用移液器移取 10ml 50xTAE 溶液。向烧杯加注 490ml 去离子水后混合充分。

3）70%乙醇：70ml 无水乙醇加兑 30ml 去离子水，充分混匀。

2.4.3 试验步骤

2.4.3.1 DNA 提取

1）将保存于-70 摄氏度的血细胞标本置于室温环境下，使之温度逐渐回复。

选用 15ml 离心管一支，吸取 1ml 标本加入所选取的离心管。

2）使用移液器吸取 5ml 细胞裂解液加入上述离心管中，颠倒 10 次左右并震荡试管，使之混合充分。

3）将离心管置于室温状态约 10 分钟，以利于裂解液充分发挥作用，期间需重复前述颠倒试管过程数次。

4）将离心管置于离心机中，转速选择 2000 转，离心时间持续 10 分钟。倒掉上清液，尽可能将残余上清吸弃彻底，勿触及白色沉淀物。反复精细操作后，最终使 15ml 离心管余留管底白色沉淀及不超过 100μL 的残留上清。

5）将带有白细胞沉淀的试管置于漩涡振荡器上，反复震荡以使其混匀。重悬的过程能使白细胞充分分散。

6）选取细胞核裂解液 1ml 加入至重悬后的试管中。使用移液器枪头反复吸放试管中的溶液使之充分混匀，待试管内混合物渐变粘稠状，旋转并颠倒试管数次。重复上述过程直至核裂解液与白细胞充分反应。

7）向经过核裂解后的试管内加入蛋白沉淀液 0.5ml，使用漩涡振荡器激烈震荡 30 秒左右。重复此过程直至观察到小的蛋白团块。

8）2500 转室温状态下离心 10 分钟，管底出现深棕色的蛋白沉淀物。

9）选取 1.5ml 离心管，加入约 0.5ml 室温异丙醇，吸取带有蛋白沉淀物试管的上清液约 0.5ml 加入该管。轻微颠倒试管多次以使之混匀充分，重复该过程直至管中出现絮状或线状的 DNA 沉淀。

10）将试管垂直放置，待 DNA 沉淀沉至管底。13000 转离心约 1 分钟后，管底可见明显白色 DNA 沉淀块。

11）使用移液器移弃上清液，加入 1ml 室温 70%乙醇，颠倒试管数次以漂洗 DNA 沉淀。13000 转离心 1 分钟，倒弃上清液，使用枪头小心吸弃沉淀周围及管壁的残留乙醇。倒置离心管于干净吸水纸上，室温下干燥数分钟。

12）吸取 DNA 溶解液 100μL 加入到离心管中，使 DNA 沉淀再次溶解。轻弹管壁使之混匀，于 65 摄氏度孵育 1 小时致充分溶解。

2.4.3.2 DNA 鉴定及稀释

1）移液器吸取溶解后的 DNA 溶液 1μL,加去离子水至 50μL。用紫外分光光度计测量波长在 260nm 及 280nm 时的 OD 值。

2）OD280 的值能够反映蛋白质的污染情况。当 OD260/OD280＞1.7 时，表明提取的 DNA 纯度符合实验要求。此值在 1.8 左右尤佳。

3）进行 PCR 的 DNA 浓度需为 10ng/μL，根据测定的 DNA 原液浓度，具体稀释。DNA 浓度测定公式：DNA（μg/μL）=OD260x50x 稀释倍数/1000。

2.4.3.3 SNP 测定

1）PCR 引物设计结合 NCBI 数据库的 renalase 全基因组序列，参照 Zhao 等[7]研究，引物序列如下（表 1）：

表 1 各 SNP 位点引物设计

SNP		引物序列
rs10887800	Upper Primer	5' CAG GAA AGA AAG AGT TGA CAT 3'
	Lower Primer	5' AAG TTG TTC CAG CTA CTG T 3'
rs2576178	Upper Primer	5' AGC AGA GAA GCA GCT TAA CCT 3'
	Lower Primer	5' TTA TCT GCA AGT CAG CGT AAC 3'
rs2296545	Upper Primer	5' GGA AGT CCC CGA TCA CGT GAC 3'
	Lower Primer	5' TGC TGT GTG GGA CAA GGC TGA 3'

引物由南京百斯凯科技有限公司合成。引物先经 4000rpm 离心 2 分钟，然后使用 1x 灭菌 TE 溶解并稀释，最终制得 10pmol/μL 的工作液，于-20 摄氏度冷冻保存。

2）PCR 反应体系及条件

使用 2xEasy Taq PCR super Mix 对 renalase 目标位点的基因片段进行扩增，反应体系及条件见表 2、3。

表 2 PCR 反应体系

组分	容量
2xEasy Taq PCR super Mix	10μL
DNA 模板	1μL
Upper and Lower Primer	2μL（1+1）
去离子水	加至 20μL

表 3 PCR 反应条件

SNP	退火温度(℃)	延伸时间（s）	循环次数（次）
rs10887800	60	40	32
rs2576178	61	40	32
rs2296545	64	40	30

反应后的产物使用特定的限制性内切酶进行切割。（详见表 6）

3）凝胶电泳

①琼脂糖凝胶制备：

表 4 琼脂糖凝胶浓度与线性 DNA 分离范围参数

凝胶浓度（%）	0.5	0.7	1.0	1.2	1.5	2.0
DNA 长度（bp）	100~30000	800~12000	500~10000	400~7000	200~3000	50~2000

结合本研究中三个 SNP 位点经限制性内切酶切割后 DNA 片段长度，选择制

备凝胶的浓度为 1.5%。

a)天平称取琼脂糖 0.3g，量筒量取 1xTAE 溶液 20ml，两者于 100ml 锥形瓶中混合均匀。

b))将本瓶放置于微波炉中加热，反复煮沸至琼脂糖充分溶解，此时溶液呈清澈透明状。

c)取出溶液置于室温环境下，冷却至 60℃左右时加入 10mg/ml 的 EB 溶液 2μL，混合均匀后倒入制胶模具（预先备好的制胶模具两端封闭切厚度不超过 0.5cm），迅速插入齿梳。

d)待琼脂糖凝胶凝固完全后，将齿梳拔除。

e)将制备的琼脂糖凝胶加入电泳槽内，加入缓冲液（1xTAE）至超出胶面约 1mm。

②电泳

在 3μL 酶切后样品中加入 5μL 缓冲液，使用移液器移取注入电泳槽的一侧的加样孔。

接通电源后，选择合适的电压电泳半小时左右。

③成像

本研究使用的是美国 UVP 公司的 GD8000 型紫外凝胶成像系统。使用该仪器在合适的曝光条件下，显影并保存。

2.5 统计学处理

本研究主要使用 SPSS 19.0 及 Haploview 4.2 两大软件进行数据统计分析。计量资料以均数±标准差表示，多组间差异选用 kruskal-wallis 检验；计数资料以百分比或具体数量表示，不同组间的差异选用 X^2检验。使用 Haploview 软件进行基因型分析、等位基因频率分析、HWE 检验、SNP 位点之间的连锁不平衡分析（D 及 r^2代表各位点之间的 LD 强度）以及单体型的分析[11-13]。Renalase 基因多态性与高血压及合并冠心病之间的风险程度采用 OR 值及 95%CI 表示，这两项的数值通过非条件性 logistic 回归来计算。临床一般资料及各项生化指标与 renalaseSNP 各基因型之间的关系的比较采用线性回归。当 $P<0.05$ 时，差异具有统计学意义。

3 结果

3.1 研究人群的临床特征

本研究共入选 625 人，其中单纯高血压组 202 例，高血压合并冠心病组 191 例，健康对照组 232 例。关于一般资料，三组人群性别、年龄、吸烟饮酒情况均无统计学差异；两组病例组的 BMI 与健康对照组有显著差异。关于生化指标，Na、TG 无统计学差异；三组间 Glu、TC、LDL-C、HDL-C、Cr 均差异显著，前三项指标遵循高血压合并冠心病组＞高血压组＞健康对照组的规律，HDL-C 与之相反。（见表 5）

表 5 三组人群临床一般资料及生化指标比较

变量	HBP+CHD 组（n=191）	HBP 组 (n=202)	对照组 (n=232)	P 值
性别（男/女）	102/89	107/95	109/123	P>0.05
年龄（岁）	56.4±9.7	54.8±8.9	57.3±10.2	P>0.05
BMI（kg/m²）	26.53±3.71	25.98±3.41	23.02±3.17	P<0.05
GLU（mmol/L）	6.51±1.76	5.98±1.69	5.37±1.28	P<0.05
TG（mmol/L）	1.68±0.89	1.71±1.02	1.72±0.96	P>0.05
TC（mmol/L）	5.35±0.84	5.21±0.99	4.78±1.01	P<0.05
HDL-C（mmol/L）	1.19±0.32	1.22±0.31	1.39±0.42	P<0.05
LDL-C（mmol/L）	4.21±0.97	3.17±1.16	2.71±1.24	P<0.05
Cr(μmol/L)	70.29±13.27	74.27±15.21	66.15±11.29	P<0.05
Na(mmol/L)	139.27±4.28	140.19±5.11	ND	P>0.05
吸烟比（%）	41.5%	41.7%	39.8%	P>0.05
饮酒比（%）	29.3%	31.0%	30.1%	P>0.05

注：HBP+CHD 组：高血压合并冠心病组　　HBP 组：高血压组（下同）

3.2 各 SNP 基因型鉴定图

我们根据对样本进行基因测序的方法对酶切的结果进行检验，参照既往的研究，本研究入选的 3 个位点的酶切片段见表 6，其基因型分型电泳图见图 2-4.

表 6 限制性内切酶结果分析

SNP	限制性内切酶	酶切后片段长度
Rs10887800	PstI	A: 554bp<br>G:415bp+139bp
Rs2576178	MspI	A:525bp<br>G:423bp+102bp
Rs2296545	Eco81I	C:188bp+21bp<br>G:209bp

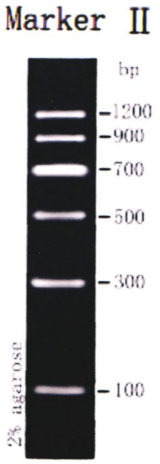

图 1 琼脂糖凝胶电泳 MARK 带

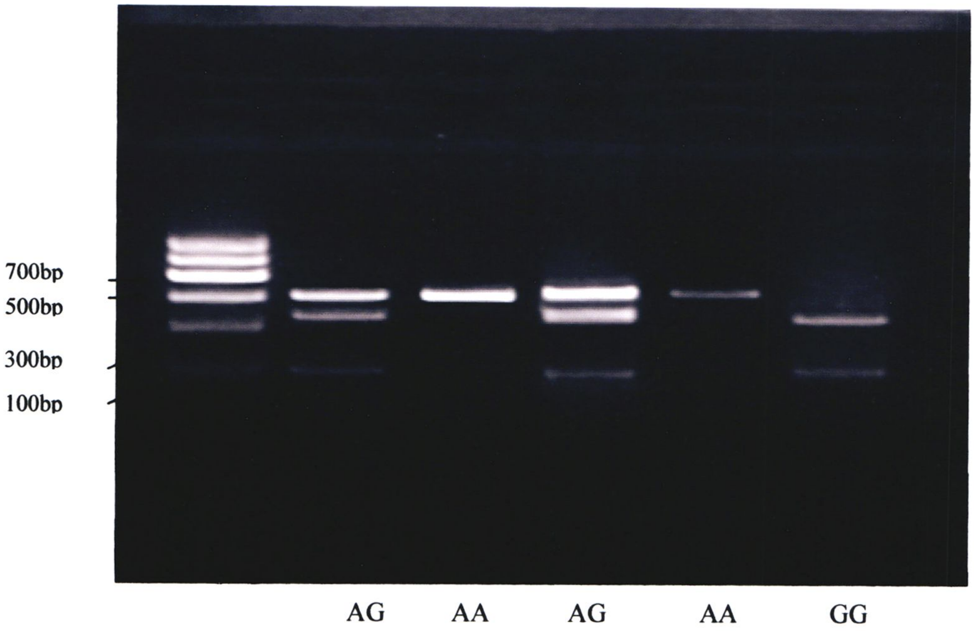

图 2. Rs10887800 电泳图

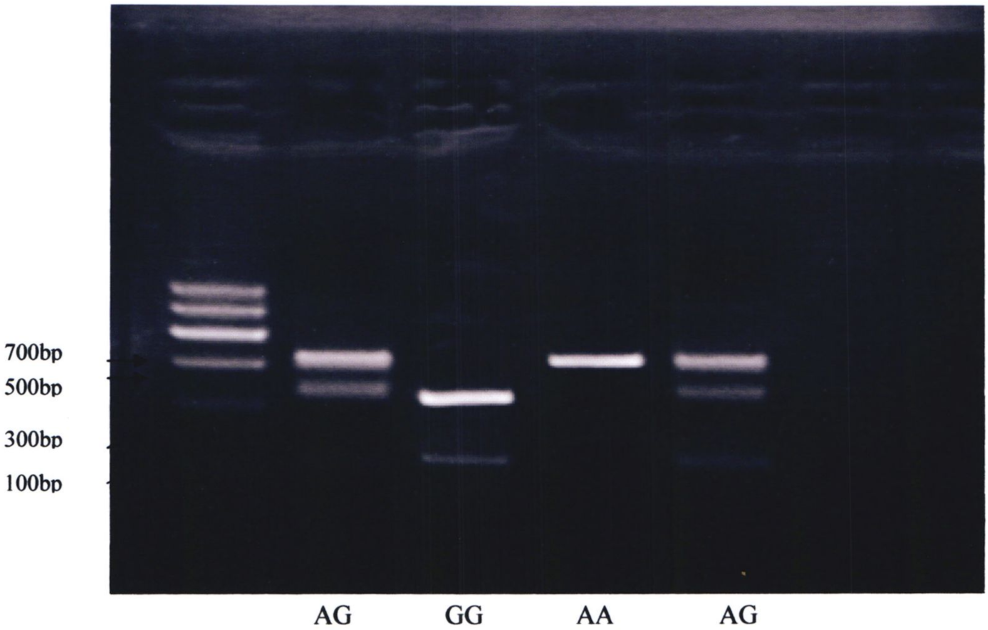

图 3 rs2576178 电泳图

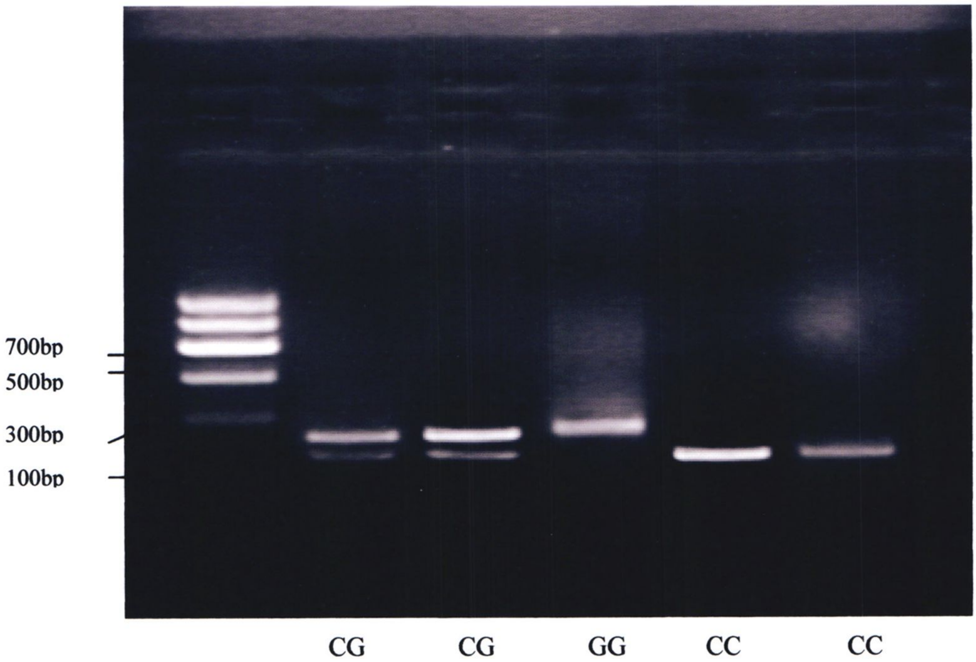

图 4 Rs2296545 电泳图

3.3 SNP 的 HWE 检验及连锁不平衡分析

哈迪-温伯格遗传平衡是指在一个足够大且婚配自由的种群内，没有自然选择、突变及新基因加入的理想状态下，表达各种性状的各等位基因及基因型的频率将保持稳定不变。运用 Haploview4.2 软件进行 HWE 检测时，受检组的自由度（df=1),P＞0.05，则代表该组样本符合 HWE。本研究中，我们对 3 组进行的 HWE 检测显示，3 个 SNP 在 3 组中均符合 HWE。代表本研究所选取的对象来自普通人群，有一定的代表性。

连锁不平衡，亦称等位基因关联。是指处于同一染色体上的两个等位基因的非随机关联。LD 的出现是由基因突变或重组造成的。本研究三个位点间连锁不平衡模式见图 5 及表 7，其连锁强度均不高，其相互预测的价值均不高，我们将对其进行逐一分析。

表 7 各位点间的连锁不平衡关系

D/r^2	Rs2576178	Rs2296545
Rs10887800	0.166/0.025	0.154/0.016
Rs2576178	-	0.040/0.001

各位点间的连锁不平衡模式图

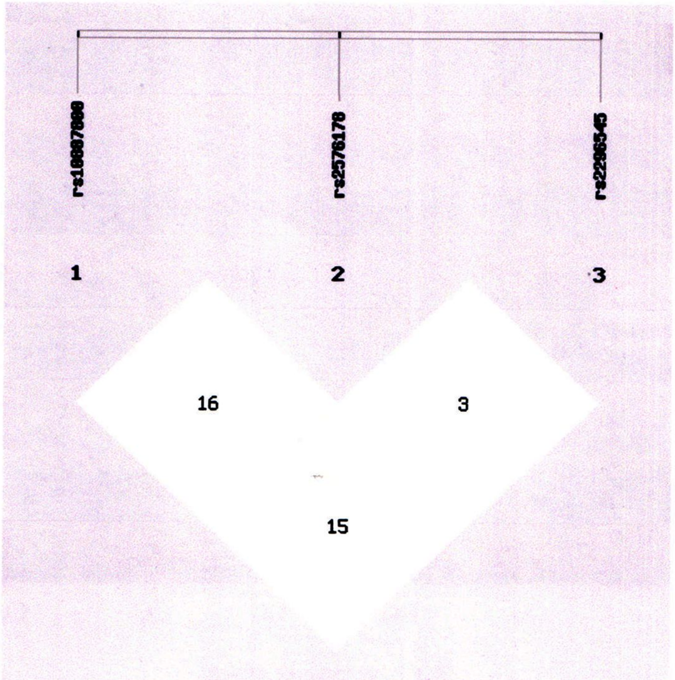

图 5 三个 SNP 间连锁不平衡模式图 （D'X 100%）

3.4 Renalase 基因三组样本统计学分析

3.4.1 rs10887800 基因型关联分析

本研究采取的以每个 SNP 位点逐一进行三组间各基因型及等位基因频率比较，进而进行相互两两比较的方式。高血压合并冠心病组、单纯高血压组、对照组之间的renalase基因rs10887800位点基因型及等位基因频率的结果可见表N。表中分别展示了高血压合并冠心病组、单纯高血压组及对照组人群的基因型分布、等位基因频率、相关等位基因危险程度比较以及 rs10887800 位点在各组的 HWE 检验值。结果可见，该位点在三组间的等位基因频率差异无统计学意义，基因型的差异有统计学意义（P=0.027）。我们继续进行两两比较，分析三组之间相互的差异，结果见表 8-11。结果显示，单纯高血压组与对照组、高血压合并冠心病组与对照组之间的基因型及等位基因频率差异均无统计学意义，而高血压合并冠心病组与单纯高血压组之间进行比较时发现，其等位基因频率仍无统计学意义，但是基因型间的差异明显，可见高血压合并冠心病组的 GG 型明显高于单纯高血压组（P=0.009），鉴于其等位基因频率未见有统计学意义的差异，该基因型可能是高血压进展为高血压合并冠心病的独立危险因素。

表 8 三组人群 rs10887800 各基因型及等位基因频率分析

SNP	HBP+CHD 组（n=191）	HBP 组（n=202）	对照组（n=232）	P 值
Rs10887800				
AA	38（0.199）	36（0.178）	51（0.220）	0.027
AG	94（0.492）	128（0.634）	127（0.547）	
GG	59（0.309）	38（0.188）	54（0.233）	
A 等位基因	170（0.445）	200（0.495）	229（0.494）	0.103
G 等位基因	212（0.555）	204（0.505）	235（0.506）	
HWE 检测	X^2=0.002562<br>P=0.960	X^2=5.449065<br>P=0.082	X^2=2.094271<br>P=0.148	

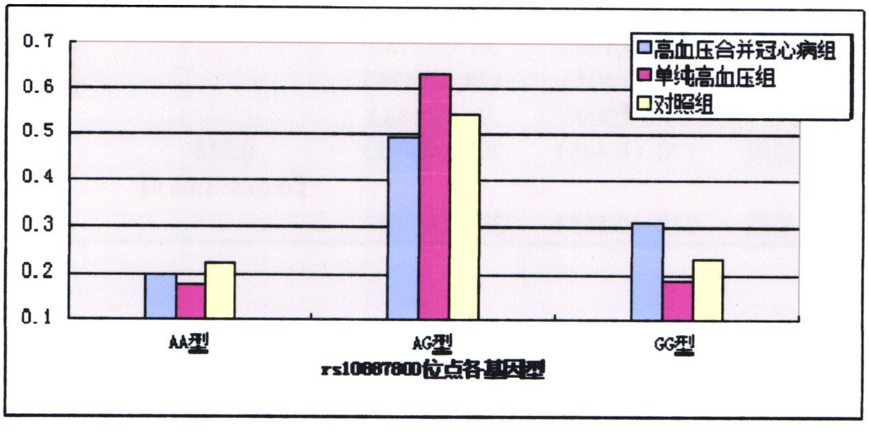

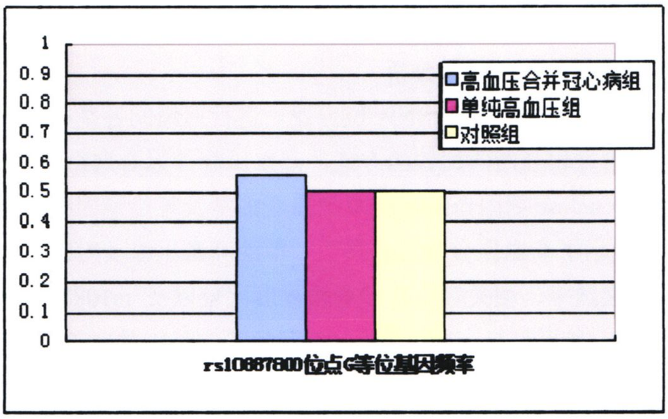

表 9 单纯高血压组与对照组 rs10887800 基因型与等位基因频率比较

SNP	HBP 组 (n=202)	对照组 (n=232)	OR（95%CI）	P 值
rs10887800				
AA	36（0.178）	51（0.220）		0.191
AG	128（0.634）	127（0.547）		
GG	38（0.188）	54（0.233）		
A 等位基因	200（0.495）	229（0.494）	1.006 【0.771~1.314】	0.964
G 等位基因	204（0.505）	235（0.506）		

表 10 高血压合并冠心病组与单纯高血压组基因型及等位基因频率比较

SNP	HBP+CHD 组 (n=191)	HBP 组 (n=202)	OR（95%CI）	P 值
rs10887800				
AA	38（0.199）	36（0.178）		0.009
AG	94（0.492）	128（0.634）		
GG	59（0.309）	38（0.188）		
A 等位基因	170（0.445）	200（0.495）	0.818 【0.618~1.083】	0.160
G 等位基因	212（0.555）	204（0.505）		

表 11 高血压合并冠心病组与对照组 rs10887800 基因型及等位基因频率比较

SNP	HBP+CHD 组 (n=191)	对照组 (n=232)	OR（95%CI）	P 值
rs10887800				
AA	38（0.199）	51（0.220）		0.212
AG	94（0.492）	127（0.547）		
GG	59（0.309）	54（0.233）		
A 等位基因	170（0.445）	229（0.494）	0.823【0.627~1.080】	0.160
G 等位基因	212（0.555）	235（0.506）		

3.4.2 rs2576178 基因型关联分析

高血压合并冠心病组、单纯高血压组、对照组间 rs2576178 位点的基因型及基因频率分析结果可见表 12-15。结果可见三组间的基因型及等位基因频率均差异显著。进一步进行两两组间分析比较，见表 12-15。我们发现 rs2576178 位点在单纯高血压组及对照组之间的基因型及等位基因频率均无具有统计学意义的差异，P 值分别为 0.688 和 0.979。而在高血压合并冠心病组与单纯高血压组间的比较，我们可见存在显著性差异，该位点在高血压合并冠心病组的 AA 型及 A 等位基因频率均显著高于单纯高血压组，P 值均为 0.001。A 等位基因的风险比 OR 值为 1.625，其 95%CI 未横跨 1，代表其 A 等位基因的风险亦有统计学意义。高血压合并冠心病组与对照组间比较可见类似的现象，病例组的 AA 型及 A 等位基因频率均显著高于对照组，且风险比提示 A 等位基因携带者具有高风险，OR 值为 1.619。

表 12 三组人群 rs2576178 基因型及等位基因频率分析

SNP	HBP+CHD 组 (n=191)	HBP 组 (n=202)	对照组 (n=232)	P 值
Rs2576178				
AA	76（0.398）	47（0.233）	59（0.254）	0.031
AG	88（0.461）	112（0.554）	119（0.513）	
GG	27（0.141）	43（0.213）	54（0.233）	
A 等位基因	240（0.628）	206（0.510）	237（0.511）	0.017
G 等位基因	142（0.372）	198（0.490）	227（0.489）	
HWE 检测	X^2=0.035405 P=0.851	X^2=2.415218 P=0.120	X^2=0.160946 P=0.688	

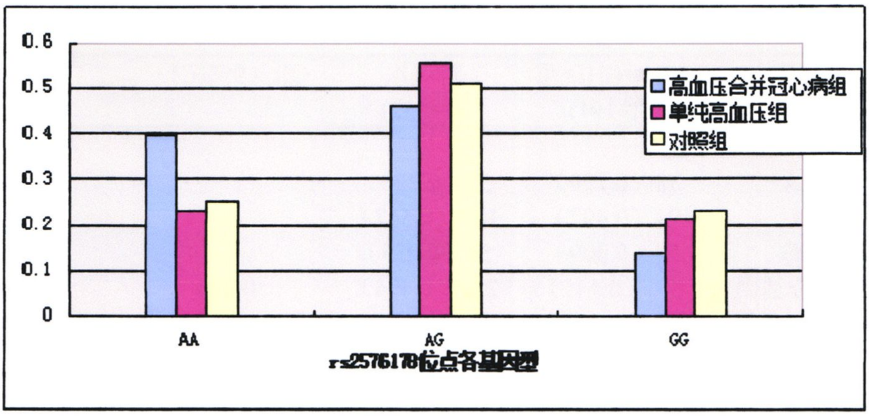

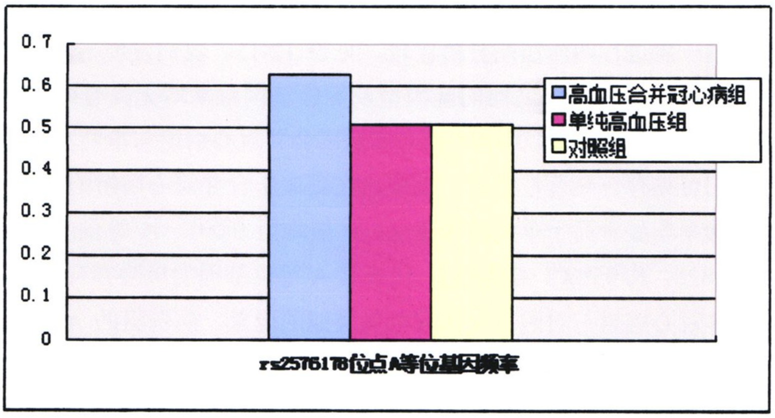

表 13 单纯高血压组与对照组 rs2576178 基因型及等位基因频率比较

SNP	HBP 组（n=202)	对照组 (n=232)	OR（95%CI）	P 值
rs2576178				
AA	47（0.233）	59（0.254）		0.688
AG	112（0.554）	119（0.513）		
GG	43（0.213）	54（0.233）		
A 等位基因	206（0.510）	237（0.511）	0.997【0.763~1.301】	0.979
G 等位基因	198（0.490）	227（0.489）		

表 14 高血压合并冠心病组与单纯高血压组 rs2256178 基因型及等位基因频率比较

SNP	HBP+CHD 组（n=191）	HBP 组 (n=202)	OR（95%CI）	P 值
rs2576178				
AA	76（0.398）	47（0.233）		0.001
AG	88（0.461）	112（0.554）		
GG	27（0.141）	43（0.213）		
A 等位基因	240（0.628）	206（0.510）	1.625【1.221~2.160】	0.001
G 等位基因	142（0.372）	198（0.490）		

表 15 高血压合并冠心病组与对照组 rs2576178 基因型及等位基因频率比较

SNP	HBP+CHD 组（n=191)	对照组 (n=232)	OR（95%CI）	P 值
rs2576178				
AA	76（0.398）	59（0.254）		0.003
AG	88（0.461）	119（0.513）		
GG	27（0.141）	54（0.233）		
A 等位基因	240（0.628）	237（0.511）	1.619【1.228~2.133】	0.001
G 等位基因	142（0.372）	227（0.489）		

3.4.3 rs2296545 基因型关联分析

Rs2296545 位点在高血压合并冠心病组、单纯高血压组、对照组之间的基因型及等位基因频率的关联可见表 16-19。结果显示三组间比较，基因型及等位基因频率的差异均无统计学意义。进而两两组间分析，见表 NNN。单纯高血压组与对照组比较，可见单纯高血压组的 CC 基因型及 C 等位基因频率均明显高于对照组，且差异有统计学意义，基因型及等位基因频率差异的 P 值分别为 0.037、0.009。C 等位基因的风险比为 1.436。而高血压合并冠心病组与单纯高血压组之间、高血压合并冠心病组与对照组之间的基因型及等位基因频率的差异均无统计学意义。

表 16 三组人群 rs2296545 基因型及等位基因频率分析

SNP	HBP+CHD 组（n=191）	HBP 组(n=202)	对照组(n=232)	P 值
Rs2296545				
CC	74（0.387）	78（0.386）	67（0.289）	0.179
CG	78（0.408）	93（0.460）	111（0.478）	
GG	39（0.204）	31（0.153）	54（0.233）	
C 等位基因	226（0.592）	249（0.616）	245（0.528）	0.082
G 等位基因	156（0.408）	155（0.384）	219（0.472）	
HWE 检测	X^2=3.580919 P=0.062	X^2=0.141919 P=0.706	X^2=0.372862 P=0.541	

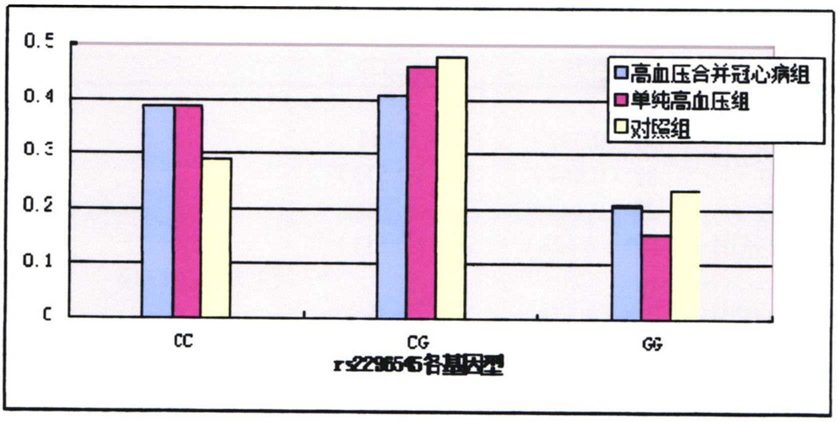

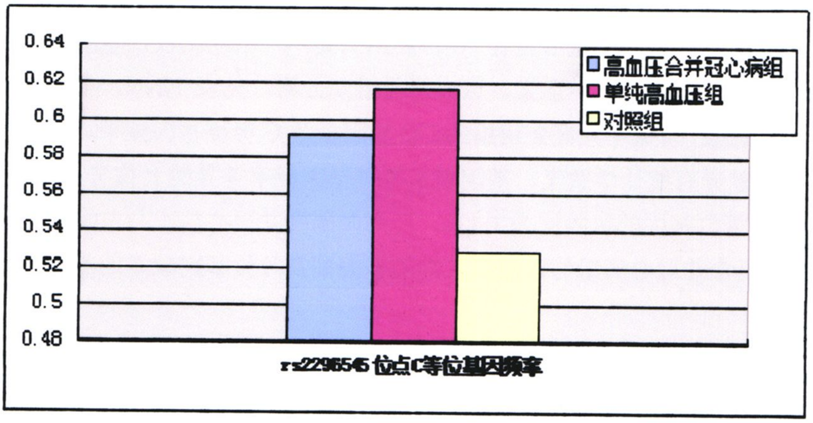

表 17 单纯高血压组与对照组 rs2296545 基因型及等位基因频率比较

SNP	HBP 组（n=202)	对照组 (n=232)	OR（95%CI）	P 值
rs2296545				
CC	78（0.386）	67（0.289）		0.037
CG	93（0.460）	111（0.478）		
GG	31（0.153）	54（0.233）		
C 等位基因	249（0.616）	245（0.528）	1.436【1.095~1.883】	0.009
G 等位基因	155（0.384）	219（0.472）		

表 18 高血压合并冠心病组与单纯高血压组 rs2296545 基因型等位基因频率比较

SNP	HBP+CHD 组（n=191）	单纯高血压组 (n=202)	OR（95%CI）	P 值
rs2296545				
CC	74（0.387）	78（0.386）		0.363
CG	78（0.408）	93（0.460）		
GG	39（0.204）	31（0.153）		
C 等位基因	226（0.592）	249（0.616）	0.902【0.677~1.200】	0.479
G 等位基因	156（0.408）	155（0.384）		

表 19 高血压合并冠心病与对照组 rs2296545 基因型及等位基因频率比较

SNP	HBP+CHD 组（n=191）	对照组 (n=232)	OR（95%CI）	P 值
rs2296545				
CC	74（0.387）	67（0.289）		0.100
CG	78（0.408）	111（0.478）		
GG	39（0.204）	54（0.233）		
C 等位基因	226（0.592）	245（0.528）	1.295【0.985~1.702】	0.064
G 等位基因	156（0.408）	219（0.472）		

3.5 renalase 基因单体型分析

本研究除了进行各个位点单独的统计分析，还进行了单体型的关联分析。

一条染色体区域内所有 SNP 位点等位基因的集合称为单体型，亦称单倍型。当存在 N 个 SNP 位点时，其理论上存在 2^n 个单体型，由于连锁不平衡的存在，当连锁的强度越高，位点间的相互连锁推测的价值也越高，其相应的单体型数目也较理论值少。我们通过单体型的分析，不仅能分析其与相关疾病之间可能存在的关联，而且因为单体型整合了多个位点的遗传信息，通过整体局部的综合分析，更能体现疾病可能的相关易感基因。本研究中分析了 renalase 的三个位点，其连锁不平衡的强度均不高，在单体型的分析当中，我们发现由 3 个位点构成的 8 个单体型中，我们忽视频率小于 0.05 的类型，共有 6 种单体型纳入分析统计。结果见表 20-22。我们分析了两两间的差异。结果显示，单体型 G-G-C 在单纯高血压的频率显著高于对照组，这个单体型含有 rs2296545-C 等位基因；单体型 G-A-C 在高血压合并冠心病的频率明显高于单纯高血压组，这个单体型含有 rs2576178-A 等位基因；单体型 A-A-G 在高血压合并冠心病组的频率显著大于对照组，此单体型亦包含 rs2576178-A 等位基因。

表 20 单纯高血压组与对照组单体型分析

单体型	HBP 组	对照组	X^2值	P 值	OR【95%CI】
A-A-C	0.139	0.146	0.103	0.748	0.939 [0.641~1.377]
A-A-G	0.090	0.059	3.008	0.083	1.571 [0.940~2.625]
A-G-G	0.086	0.113	1.702	0.192	0.741 [0.472~1.164]
G-A-C	0.163	0.124	2.576	0.109	1.366 [0.932~2.001]
G-A-G	0.118	0.181	6.499	0.011	0.609 [0.415~0.894]
G-G-C	0.135	0.082	6.336	0.012	1.745 [1.127~2.703]

表 21 高血压合并冠心病组与单纯高血压组单体型分析

单体型	HBP+CHD组	HBP 组	X^2值	P 值	OR【95%CI】
A-A-C	0.120	0.139	0.580	0.447	0.850 [0.560~1.291]
A-A-G	0.128	0.090	2.879	0.090	1.479 [0.939~2.328]
A-G-C	0.151	0.180	1.245	0.265	0.807 [0.553~1.177]
G-A-C	0.233	0.163	6.167	0.013	1.565 [1.097~2.232]
G-A-G	0.147	0.118	1.387	0.239	1.282 [0.847~1.939]
G-G-C	0.088	0.135	4.420	0.036	0.616 [0.390~0.971]

表 22 高血压合并冠心病组与对照组单体型分析

单体型	HBP+CHD组	对照组	X^2值	P 值	OR【95%CI】
A-A-C	0.120	0.146	1.206	0.272	0.799 [0.534~1.194]
A-A-G	0.128	0.059	11.968	0.001	2.322 [1.425~3.783]
A-G-C	0.151	0.175	0.930	0.335	0.834 [0.577~1.206]
G-A-C	0.233	0.124	17.238	NS	NS
G-A-G	0.147	0.181	1.729	0.189	0.781 [0.540~1.130]
G-G-C	0.088	0.082	0.084	0.772	1.074 [0.661~1.746]

3.6 Renalase 各基因型与临床一般资料及生化指标的比较分析

将肾胺酶基因各位点基因型作为研究对象进行分组，对各基因型的临床一般资料及生化指标进行分析比较。结果见表 23-25。结果可见，rs10887800 位点在 LDL-C 项目差异具有统计学意义，GG 基因型的 LDL-C 值均高于其他两种基因型，其他各项指标均无统计学差异；rs2576178 位点的各临床指标均无明显差异；rs2296545 位点各指标均无具有统计学意义的差异。

表 23 rs10887800 各基因型的临床一般资料及生化指标的比较

变量	AA	AG	GG	P 值
年龄（岁）	55.2±10.1	54.8±9.8	55.3±7.4	P>0.05
BMI（kg/m²）	24.87±3.32	25.01±3.19	24.93±3.25	P>0.05
GLU（mmol/L）	5.67±1.34	5.71±1.29	5.59±1.21	P>0.05
TG（mmol/L）	1.70±0.94	1.68±1.07	1.69±1.12	P>0.05
TC（mmol/L）	5.01±1.02	4.97±0.87	5.05±1.07	P>0.05
HDL-C(mmol/L)	1.15±0.37	1.23±0.41	1.16±0.39	P>0.05
LDL-C(mmol/L)	3.13±1.02	2.87±0.96	4.09±1.17	P<0.05
Cr(μmol/L)	72.82±15.70	73.69±13.87	77.29±12.58	P>0.05

表 24 rs2576178 各基因型与临床一般资料及生化指标的比较

变量	AA	AG	GG	P 值
年龄（岁）	54.3±8.3	55.1±7.9	56.0±9.8	P>0.05
BMI（kg/m²）	25.39±3.16	24.73±3.31	24.81±3.18	P>0.05
GLU（mmol/L）	5.71±1.23	5.57±1.51	5.46±1.34	P>0.05
TG（mmol/L）	1.63±0.97	1.69±1.01	1.67±0.85	P>0.05
TC（mmol/L）	5.09±0.93	4.88±0.95	4.93±1.07	P>0.05
HDL-C(mmol/L)	1.13±0.30	1.22±0.41	1.27±0.39	P>0.05
LDL-C(mmol/L)	3.29±0.89	3.41±1.29	3.49±1.01	P>0.05
Cr(μmol/L)	73.18±13.01	74.01±14.19	74.29±13.20	P>0.05

表 25 rs2296545 各基因型与临床一般资料及生化指标的比较

变量	CC	CG	GG	P 值
年龄（岁）	55.1±10.1	53.9±8.7	55.6±9.6	P>0.05
BMI（kg/m²）	24.39±4.07	24.51±3.92	25.72±4.18	P>0.05
GLU（mmol/L）	5.39±1.71	5.91±1.92	5.64±1.64	P>0.05
TG（mmol/L）	1.70±0.93	1.67±1.01	1.71±0.88	P>0.05
TC（mmol/L）	5.07±0.75	4.93±0.89	5.04±1.03	P>0.05
HDL-C（mmol/L）	1.24±0.37	1.27±0.40	1.18±0.39	P>0.05
LDL-C（mmol/L）	3.50±1.01	3.39±1.17	3.42±1.08	P>0.05
Cr(μmol/L)	71.26±12.09	70.17±13.22	73.01±10.91	P>0.05

4 讨论

高血压作为最常见的慢性病，以其较高的患病率及随之带来的各靶器官风险，逐渐得到人们的重视。据临床流行病学统计，2000 年全球高血压患者约有 9.72 亿，至 2025 年，高血压患者人数将增至惊人的 15.6 亿。高血压亦是心脑血管疾病最常见的危险因素，其与冠心病、脑卒中等关系甚为密切。近年来，通过病例-对照关联方法进行各基因位点多态性分析，进而筛查出疾病的易感基因的研究较为普遍。其中，肾胺酶基因便是近年来发现的可能和高血压发病相关的一组基因。

肾胺酶，主要由肾脏分泌，其生理意义在于降解血液当中的儿茶酚胺，其中多巴胺为其最优先结合的底物[4]，肾上腺素及去甲肾上腺素次之。有研究发现[7]，肾胺酶基因的 rs2576178 和 rs229654 两个位点的多态性与高血压相关。其中，rs2576178 的 G 等位基因频率与 rs2296545 的 C 等位基因频率均与高血压强烈相关，其 P 均小于 0.0001。另有实验[8,9]发现，肾胺酶基因的 rs2296545 位点 C 等位基因频率可能和糖尿病有关。高血压脑梗病人 rs10887800G 等位基因频率是单纯高血压的 1.63 倍，揭示该位点可能是高血压病人发生脑梗的潜在危险因素。

高血压、糖尿病作为冠心病的两大危险因素，脑卒中与冠心病同源于动脉粥样硬化在不同靶器官损害所造成的临床病症，其相关的易感基因是否也是冠心病的危险因素？本实验通过对三组病人（高血压合并冠心病组、单纯高血压组、健康对照组）的病例-对照关联分析，探讨高血压及合并冠心病与肾胺酶的关系。

本研究采用的是首先进行三组基因型及等位基因频率差异比较，进而进行两两组间具体差异比较的方式。通过我们的研究发现，rs10887800 位点在高血压合并冠心病组与单纯高血压组间的等位基因频率无差异，但是基因型在两组间存在差异（p=0.009)，其中 GG 型在高血压合并冠心病组的频率高于单纯高血压组；rs2576178 位点的 A 等位基因在高血压合并冠心病组的等位基因频率显著高于单纯高血压组及健康对照组（p=0.001)，且基因型亦存在差异（p=0.001 ；p=0.003)，而单纯高血压组与对照组间差异无统计学意义，提示该位点的 A 等位基因可能是冠心病的易感因子，相应的 G 等位基因可能是保护因子；rs2296545 的 C 等位基因在单纯高血压组的等位基因频率高于对照组（P=0.009)，高血压组 CC 基因型比率较高（p=0.037)，其他组间差异无统计学意义，提示该位点的 C 等位基因可能是高血压的易感因子，其相应的 G 等位基因可能是保护因子。通过单体型整合的位点信息分析发现，之于单纯高血压组与对照组之间存在统计学差异的 G-G-C 型，其中包含了 rs2296545 位点的 C 等位基因；单纯高血压组与高血压合并冠心病组之间存在统计学差异的 G-A-C 型，亦包含了 rs2576178 位点的 A 等

位基因，进一步提示这两个位点与疾病的关联。

本研究肾胺酶基因多态性与高血压的关联分析与国外报道的不完全一致，可能基于研究对象种群的差异性。本研究为了避免人群分层与混合所带来的假阳性可能，选取的病例均为中国南方汉族人群，其客观上可能和不同人群种族的renalase基因SNP存在差异。本研究进一步证实了肾胺酶基因对高血压的易感性，但肾胺酶是通过何种途径与机制对血压进行调控仍不甚明晰[14-17]。肾胺酶是通过降解血液循环中的儿茶酚胺等物质来调控血压，还是通过对影响肾脏水钠重吸收的条件来影响血压（肾脏组织可以产生多巴胺来进行肾小管钠转运的调节），亦或是其他机制目前仍不明朗，需要更精细的功能学研究来证实，结合基因多态性研究来最终明确肾胺酶与高血压的关系[18]。

通过我们的研究还发现高血压合并冠心病与肾胺酶可能存在的关联，从病因学的角度分析及讨论，其关联性具有逻辑性。SNP位点rs2576178位于肾胺酶基因的5’侧翼，其可能通过对转录起始的影响来改变基因的功能。高血压冠心病与肾胺酶的相关性需要在更多人群中得到重复验证，以及展开的相应功能学研究来明确他们的关系。

5 结 论

Rs2576178 位点的 A 等位基因可能是高血压罹患冠心病的易感因子，其对应的G等位基因可能是保护因子，AA 基因型的高血压患者罹患冠心病的风险增高。

Rs2296545 位点的 C 等位基因可能是高血压的易感因子，其对应的 G 等位基因可能是保护因子，CC 基因型的人群患高血压的风险增高。

参考文献

[1] Lubas A,Zelichowski G,Prochnicka A,Wisniewska M,Wankowicz Z.Renal autoregulation in medical therapy of renovascular hypertension.Arcb Med Sci 2010;6:912-918.

[2] Banach M,Kjeldsen SE,Narkiewicz K.Controversies in hypertension treatment.Curr Vasc Pharmacol 2010;8:731-732.

[3] Malyszko J,Zbroch E,Malyszko JS,Koc-Zorawska E,Mysliwiec M.Renalase was not related to blood pressure,but to residual renal function in haemodialysis and peritoneal dialysis patients.J Am Soc Nephrol 2011;22:723A.

[4] Xu J,Li G,Wang P,Velazquez H,Yao X,Li Y,et al.Renalase is a novel ,soluble monoamine oxidase that regulates cardiac function and blood pressure.J Clin Invest 2005;115:1275-1280.

[5] Wu Y,Xu J,Velazquez H,Wang P,Li G,Liu D,et al.Renalase deficiency aggravates ischemic myocardial damage.Kidney Int 2011;79:853-860.

[6] Hennebry SC,Eikelis N,Socratous F,Desir G,Lambert G,Schlaich M.Renalase,a novel soluble FAD-dependent protein,is synthesized in the brain and peripheral nerves.Mol Psycbiatry 2010;15:234-236.

[7] Zhao Q,Fan Z,He J,Chen S,Li H,Zhang P,Wang L,et al.Renalase gene is a novel susceptibility

Gene for essential hypertension:a two -stage association study in northern Han Chinese population.J Mol Med 2007;85:877-885.

[8] Buraczynska M,Zukowski P,Buraczynska K,Mozul S,Ksiazek A.Renalase Gene polymorphisms in patients with Type 2 Diabetes,Hypertension and Storke.Neuromol Med 2011;13:321-327.

[9] Stec A,Semczuk A,Furmaga J,Ksiazek A,Buraczynska M.Polymorphism of the renalase gene in end-stage renal disease patients affected by hypertension.Nephrol Dial Transplant 2012;27:4162-4166.

[10] Buraczynska M,Zukowski P,Buraczynska K,Mozul S,Ksiazek A.Renalase Gene polymorphisms in patients with Type 2 Diabetes,Hypertension and Storke.Neuromol Med 2011;13:321-327.

[11] Lake SL,Lyon H,Tantisira K,Silverman EK,Weiss ST,Laird NM,Schaid

DJ.Estimation and tests of haplotype-environment interaction when linkage phase is ambiguous.Hum Hered.2003;55:56-65.

[12] Guo SW,Thompson EA.Performing the exact test of Hardy-weinberg proportion for multiple alleles.Biometrics.1992;48:361-372.

[13] Barrett JC,Fry B,Maller J,Daly MJ.Haploview:analysis and visualization of LD and haplotype maps.Bioinformatics.2005;21:263-265.

[14] Pandini V,Ciriello F,Tedeschi G,Rossoni G,Zanetti G,Aliverti A.Synthesis of human renalase1 in Escherichia coli and its purification as a FAD-containing holoprotein.Protein Expr Purif 2010;72:244-253.

[15] Luft FC.Renalase,a catecholamine-metabolizing hormone from the kidney.Cell Metab 2005;1:358-360.

[16] Eikelis N,Hennebry SC,Lambert GW,Schlaich MP.Does renalase degrade catecholamines?Kidney int 2011;79:1380.

[17] Milani M,Ciriello F,Barono S,Pandin V,Canevari G,Bolognesi M,Aliverti A.FAD-binding site and NADP reactivity in human renalase:a new enzyme involved in blood pressure regulation.J Mol Biol 2011;411:463-473.

[18] Lubas A,Zelichowski G,Prochnicka A,Wisniewska M,Wankowicz Z.Renal autoregulation in medical therapy of renovascular hypertension.Arcb Med Sci 2010;6:912-918.

综 述

肾胺酶研究进展

【摘要】肾胺酶是近年新发现的一种酶，主要由肾脏分泌，能降解循环中的儿茶酚胺，可能在对心血管的调控方面存在重要作用。多项临床研究发现其可能与高血压、糖尿病、脑卒中相关。但目前关于如何检测肾胺酶及衡量其活性仍存在争议。关于肾胺酶的激活及抑制剂目前仍不明了。肾胺酶是否能成为心血管领域的另一个方向，有待进一步研究。

【关键词】肾胺酶；高血压；争议

一. 肾胺酶概述

肾脏作为人体内重要器官，不仅仅具有排泄作用，其相应的内分泌功能也越来越得到重视。肾胺酶便是近年来新发现的一种主要由肾脏分泌的生物大分子。2005 年，Xu[1]等首次系统性的阐述了肾胺酶及其可能的生物学功能，揭开了这个生物分子的神秘面纱。

通过现有的基因数据库信息可知，肾胺酶基因位于 10 号染色体 q23.33，包含有 7 个外显子，存在 2 处转录变异，能编码 4 种具有组织特异性的剪接异构体[2]（hRenalase1-4)。目前在人体血液中，仅能检测出 hRenalase1 型，提示具有组织特异性的另外三种亚型可能具体不同的功能[2]。肾胺酶的分子量约为 38KDa。目前大鼠的肾胺酶基因检测显示，该基因位于大鼠的 19 号染色体 C1，并且在氨基酸序列同源性检测方面，大鼠肾胺酶基因（mRenalase）与人类血液中 1 型肾胺酶存在约 72%同源[3]。Xu[1]等人的研究发现，肾胺酶存在黄素腺嘌呤二核苷酸结合结构域，并且黄素腺嘌呤二核苷酸可能是维持肾胺酶稳定及使其体现单胺氧化酶活性的潜在辅助因子。但是肾胺酶的氨基酸序列与在人体内催化儿茶酚胺氧化脱氨的 FAD 依赖性酶（MAO-A）明显地不同。肾胺酶仅有 13.2%氨基酸序列相似[1]。肾胺酶优先表达于肾小管近端，其次肾小球及远端肾小管，在心肌细胞、肝脏及骨骼肌亦有肾胺酶的表达[1]。最近研究表明，周围神经、中枢神经、内皮细胞、肾上腺以及脂肪组织均有肾胺酶的表达[2,4]。

二. 肾胺酶相关基础研究

Xu 等人的研究提示肾胺酶主要由肾脏分泌，能够降解血液循环中的儿茶酚胺，并且可能在交感紧张与血压调控方面扮演重要的角色[1]。该研究将肾胺酶注射进 SD 大鼠体内，导致血压水平下降约 25%，同时伴有心率及心肌收缩功能的

下降[1]。这种介入手段的影响仅能持续数分钟，并且是剂量依赖性的。研究人员同时进行了α及β受体阻滞剂与肾胺酶的类比研究，未见两者具有类似性。将肾胺酶基因敲除后进行传代培养的大鼠试验能够更好的诠释这种酶可能存在的作用。Wu[5]等人报道，野生型大鼠与肾胺酶基因敲除大鼠之间，在肾功能方面无明显差异，但是肾胺酶基因敲除大鼠较之野生大鼠，普遍存在血压水平更高、心率更快、血液中儿茶酚胺水平更高的现象。研究人员据此提出假设，基因敲除的大鼠可能对心肌缺血更易感[5]。通过建立心肌缺血再灌注模型，研究人员对两组大鼠分别施予心肌缺血处理，他们发现，肾胺酶缺乏的大鼠在经历急性心肌缺血后心肌损害较之野生大鼠更为严重，并且注射重组肾胺酶后能明显缓解心肌损伤。因此他们得出结论，心肌组织的肾胺酶缺乏增加心肌缺血时的心肌损害。有研究报道[6]，在基础条件下（即肾胺酶前体）循环系统中的肾胺酶缺乏足够的单胺氧化酶活性，但是在血压正常的大鼠体内，当循环中的肾上腺素水准激增，不超过2分钟即可观察到肾胺酶活性增加的一个峰值。同时，当血压水平增加超过5mmHg时，肾胺酶前体大部分能被激活。有学者将大鼠肾脏切除5/6后，发现其术后2-3周出现了血液肾胺酶的严重缺乏[6]。而且肾脏切除后的大鼠表达的微量肾胺酶也转变为分子量接近70KDa的二聚体形式。而且肾上腺素的滴注较之普通大鼠，其肾胺酶活性激活的时间更短，峰值更低。研究人员得出结论认为肾衰模型大鼠的肾上腺素水平的升高可能源于肾胺酶的缺乏[6]。

在另一个关于肾脏切除小狗的试验中，研究人员[4]发现手术组心肌组织中的去甲肾上腺素转运蛋白与肾胺酶的表达明显低于假手术组及空白对照组，但是去甲肾上腺素的水平两倍于假手术组及空白对照组。试验提示幼龄动物肾脏切除后升高的儿茶酚胺水平源于去甲肾上腺素代谢的降低。Desir[7]等人报道在盐敏感的大鼠皮下注射肾胺酶能够持续性的控制血压达近12小时。

三．肾胺酶临床相关资料

Xu[1]等研究发现终末期肾脏病患者的肾胺酶表达较之普通对照组明显减少，同样的现象在Wang[8]等人的研究中得到了体现。最近有研究发现[9]，接受肾移植的患者的血浆肾胺酶表达较之正常对照组明显升高。在这个研究中，对肾胺酶同样有预测作用的有肾功能、年龄、接受肾移植的时间以及舒张压等。Przybylowski[10]等人研究发现，类似的现象出现在接受心脏移植的患者体内，但是仅仅肾功能与其相关，其与性别、糖尿病及高血压的关系均无统计学意义。在一组临床研究中，Schlaich[11]等人分析了22个顽固性高血压患者的动脉血肾胺酶浓度（使用肾胺酶多克隆抗体的western-blot检测），发现其浓度远低于普通对照组（$p<0.05$），而且顽固性高血压组表现为较之对照组的去甲肾上腺素过度表达，但是去甲肾上腺素的差异没有体现统计学差异（$P=0.12$）。

2007 年 zhao[12]等通过病例-对照关联分析的方法，对大量人群的基因型进行鉴定及等位基因频率计算，首次对高血压与肾胺酶的关系进行筛查与分析。该研究通过对可能影响肾胺酶功能的区域进行对比分析，选取了 8 个 SNP 位点进行基因多态性分析。共入选 2586 例中国北方汉族人群，其中高血压患者 1317 例，健康对照患者 1269 例。通过一阶段初选及二阶段复筛共两阶段对比研究发现，其中 rs2576178 及 rs2296545 位点与高血压显著相关。其中 rs2576178 的 G 等位基因、rs2296545 的 C 等位基因在高血压组的等位基因频率均明显高于健康对照组并且具有统计学意义。并且 rs2576178 的 GG 型及 rs2296545 的 CC 型在高血压组亦均高于对照组。提示，肾胺酶基因可能是高血压的易感基因，其中 rs2576178 的 G 等位基因、rs2296545 的 C 等位基因均可能是高血压的易感因子，其各自对应的 C、G 等位基因可能是高血压的保护因子。

2010 年，Farzaneh-Far[13]等通过对 Heart and Soul Study 选择的 590 例高加索人进行基因型与一般临床资料及生理生化指标间的关联分析，发现肾胺酶基因 Glu37Asp 的一个功能性错位缺失所带来的多态性改变与左室肥大、收缩与舒张功能不全、运动能力较差、心肌缺血易感等因素相关，且该位点的 C 等位基因频率在病例组的等位基因频率明显高于对照组且有统计学差异。但是该实验未发现在稳定性冠心病患者中其 CC 型与高血压中间存在关联。

2011 年，Stec A[14]等研究人员分析了终末期肾脏病患者高血压与肾胺酶之间的关联，研究表明，透析病人的高血压与肾胺酶基因多态性之间存在关联且具有统计学差异。研究收录了 369 名透析病人进行 rs2576178 位点的检测，其中 200 名存在高血压，169 名血压正常。选择了 421 名透析患者进行 rs10887800 位点的检测，其中 278 名存在高血压，143 名为血压正常的对照组。结果显示，rs2576178 的 G 等位基因、rs10887800 的 G 等位基因在病例组的等位基因频率均明显高于对照组。Rs2576178 的 G 等位基因携带者患高血压的风险是对照组的 1.55 倍，而 rs10887800 则体现为 1.76 倍。利用 2 个位点分组，其各自的高血压患病率分别为 54%与 66%。Monika Buraczynska[15]等研究比较分析了肾胺酶与糖尿病、高血压及脑卒中之间的相关性，分析了 892 例病例及 400 例对照，其中 892 例又以是否存在高血压来区分。该研究中发现的有趣现象便是意外的发现了 rs10887800 与对高血压合并卒中患者的关联，该位点的 G 等位基因频率和脑梗的发生存在关联，同时也发现肾胺酶可能和糖尿病存在关联。

由于肾胺酶发现时间不长，大规模的基因与临床的流行病学分析还未得以充分的展现。当临床疾病与基因多态性分析在大量不同种群人群内得到重复验证时，说明其效性较高，再据此展开进一步的精细的基因定位监测及功能学研究，能更好的诠释这个生物大分子的功能。

四．目前存在的争议

血浆特异性酶氨基脲敏感型胺氧化酶（SSAO）能催化血液中的大多数胺类脱氨氧化形成相应的氨、乙醛及过氧化氢[16]。血液中的多巴胺及去甲肾上腺素均由氨基脲敏感型胺氧化酶催化氧化，肾上腺素却不受其代谢调节。血管粘附蛋白-1 是一种含铜的氨基脲敏感型胺氧化酶，主要由血管平滑肌细胞、脂肪细胞已经内皮细胞分泌，具有单胺氧化酶活性[17]。其具有 MAO（单胺氧化酶）的协同作用，但是对 MAO 抑制剂不敏感。其催化氧化进程可能产生有害物质，该物质可能是糖尿病病人发生动脉粥样硬化及造成心血管损害的危险因素。在动脉粥样硬化、糖尿病及肥胖群体可以观察到 SSAO 活性普遍较高[18-20]。人体内 SSAO 水平非常低，在体外培养的环境下，SSAO 可以对儿茶酚胺产生微小的影响。而且，相较于野生型动物，转基因大鼠的 SSAO 的过度表达对高血压并无特殊影响[16]。相反，SSAO 抑制剂的表达及其抗氧化性能却能导致血压水平的下降[21]。综上所述，有研究人员[22]便提出观点：SSAO 与循环中的儿茶酚胺降解无关。Boomsma and Tipton 对 Xu[1]等人检测肾胺酶活性的方法提出质疑：Xu 等检测的是 MAO 的活性，然后据此结果推测肾胺酶的活性。Boomsma and Tipton[16]认为该实验中儿茶酚胺的浓度较之儿茶酚胺病理生理浓度更高。他们进一步得出结论，肾胺酶并不是大家想象中的儿茶酚胺代谢酶，而是一种重要的可能通过其他机制调控心血管功能的酶。在另一组研究中，Pandini[23]等报道了人体肾胺酶在大肠埃希菌菌体的表达及其净化同质作用。更重要的是，他们发现这种肾胺酶与 FAD 之间的联系为非共价结合，这导致其更易游离于 SDS。尽管使用了两种不同的方法，研究人员还是没能证明肾胺酶具有 MAO 活性。然而，当将同样的方法作用于大鼠时，尽管没有表现出 MAO 催化活性，仍然能观察到其降压的作用[23]。因此目前对肾胺酶存在的质疑便是，肾胺酶是否真的是一种 MAO。Luft[24]等提到，多巴胺是同较低血压及心血管危险因素的减少相关联的的，这也是为什么多巴胺紧张度的降低会导致血压及心血管危险因素的增加。Eikelis[25]等人对肾胺酶能够降解儿茶酚胺也提出了质疑，他们认为目前没有任何相关文献或数据能够直接支持“肾胺酶能够降解儿茶酚胺”这个假说。而且他们通过对多种系株小鼠的肾胺酶基因进行基因序列分析发现，多系株小鼠的肾胺酶基因序列普遍较大鼠及人类短，并且不包含 N 端的 FAD 位点结合域，较之 Wu 等人研究差异性较大。因此很难理解这些小鼠在不存在潜在功能结合域的情况下，其肾胺酶基因是如何发挥正常作用的。Milani[26]等指出，肾胺酶不仅仅不是一种单胺氧化酶，更大的可能是其不属于氧化酶的范畴。

Xu 等研究人员使用 western-blot 技术检测多克隆抗体，他们发现相较于健康对照组，8 个进行血液透析治疗的终末期肾脏病患者的血液肾胺酶表达明显减

少。在另一研究中，实验人员制备了重组肾胺酶及其多克隆抗体。从肾脏切除患者的肾脏中提取组织，利用特定的引物进行人类肾胺酶基因的扩增，其多克隆抗体能同时结合重组肾胺酶与血浆中的肾胺酶。但是关于重组肾胺酶及其抗体的后续研究及其更深层次数据均无阐述。

western-blot 仅仅是一种半定量的方法，然而利用特异性针对肾胺酶的多克隆抗体进行的 ELISA 化验更为可取。ELISA 化验的方法从整体上检测血液中 1 型肾胺酶的水平，但不能区分检测出的蛋白是否是有活性的。而且，ELISA 方法的肾胺酶抗体可能结合不同肾胺酶亚型的片段，导致检测水平的偏高及其与肾胺酶活性的不相关性。即使 Wu 等研究人员改用 ELISA 方法进行化验，因为肾胺酶存在多种亚型的关系，实验人员也不能通过抗原-抗体结合检验的方式进行组间肾胺酶活性的比较。尽管检测的成品声称目前在人体内仅能检测出 1 型肾胺酶，但是目前仍然没有任何关于可能交叉反应的数据及报告。肾胺酶不仅仅由肾脏分泌，心肌细胞、脂肪组织、肝脏及其他器官与组织均可能表达肾胺酶，尤其在临床实验中，其检测出的水准可能非常高。实际上当我们评估肾胺酶的表达时，我们并不能确定其活性及比例，正如 Xu[1]等研究提示的，血浆中可能存在着肾胺酶的抑制剂。因此，这种酶的水平可能非常高，但是活性很低或者微不足道。

Li[6]等人发现，在试验动物体内注入儿茶酚胺能够导致血液肾胺酶活性的增高，他们发现肾胺酶常态下是以缺乏活性的前体的形式存在于循环系统中，但是当血浆中儿茶酚胺水平增高时，其前体形式迅速转变为活体形式。但是近 30 年来，我们已经认知慢性肾衰的病人血浆去甲肾上腺素水平是较高的，也导致了交感神经活性的增强。肾脏对循环中儿茶酚胺的代谢起到的作用约占 15%-24%。循环系统中的大部分儿茶酚胺是具有活性的，当他们被非神经元单胺转运体由循环系统转运至其他组织器官后，便被代谢排泄。尽管肾脏对于循环中儿茶酚胺代谢能力的下降是源于肾功能及其纤溶功能障碍，而这种代谢能力的下降导致循环中儿茶酚胺水平的升高，这种增长在肾衰病人中也可能是源于交感神经系统的激活[27]。因此我们能推断，肾功能存在衰竭病人的儿茶酚胺水平过量表达的情况下，其肾胺酶前体的激活在许多组织更迅捷有效。

近年来，关于肾胺酶的表达，研究人员进行了不少探索。但是，在人体多个器官组织广泛分布的肾胺酶不同亚型，其功能也可能存在组织特异性，而关于其不同组织作用下可能的激活及抑制因子却鲜有文献报告。仅有一处研究发现，人体尿肾胺酶与血浆肾胺酶并不完全一致，根据实验室检测，尿中肾胺酶的分子量[6](35KDa)较之循环肾胺酶更低（37.8KDa)[1,3,26]。

五. 小结

基于目前的肾胺酶进展，需要更多深层次的功能性研究来揭示其对于高血压

的可能作用及调节机制。血压的调控与心血管并发症的发生发展是一个相当复杂的进程[28-30]，其与肾胺酶的关系远未明晰。人体内特定条件下肾胺酶的浓度及活性方面的数据仍然缺乏。肾胺酶能否成为高血压治疗的标志元素仍然未知。当证实肾胺酶参与对血压调控的具体机制或途径后，重组肾胺酶的选择或许是未来的一大方向。而且，关于肾胺酶是否是单胺氧化酶这一基础问题仍然有待解决。综上所述，肾胺酶这个生物大分子，目前为止其生物学功能仍未得到充分发掘，其是否能成为一种新的治疗指标，仍然有待进一步研究。

参考文献

[1] Xu J,Li G,Wang P,Velazquez H,Yao X,Li Y,et al.Renalase is a novel ,soluble monoamine oxidase that regulates cardiac function and blood pressure.J Clin Invest 2005;115:1275-1280.

[2] Hennebry SC,Eikelis N,Socratous F,Desir G,Lambert G,Schlaich M.Renalase,a novel soluble FAD-dependent protein,is synthesized in the brain and peripheral nerves.Mol Psycbiatry 2010;15:234-236.

[3] Wang J,Qi S,Cheng W,Li L,Wang F,Li YZ,Zhang SP.Identification,expression and tissue distribution of a renalase homologue from mouse.Mol Biol Rep 2008;35:613-620.

[4] Ghosh SS,Krieg RJ,Sica DA,Wang R,Fakhry I,Gehr T.Cardiac hypertrophy in neonatal nephrectomized rats:the role of the sympathetic nervous system.Pediatr Nephrol 2009;24:367-377

[5] Wu Y,Xu J,Velazquez H,Wang P,Li G,Liu D,et al.Renalase deficiency aggravates ischemic myocardial damage.Kidney Int 2011;79:853-860.

[6] Li G,Xu J,Wang P,Velazquez H,Li Y,Wu Y,Desir GV.Catecholamines regulate the activity,secretion,and synthesis of renalase.Circulation 2008;117:1277-1282.

[7] Desir G,Tang L,Wang P,Li G,Velazquez H.Antihypertensive effect of recombiant renalase in Dahl salt sensitive(DSS)rats.J Am Soc Nephrol 2010;21:748A.

[8] Wang F,Wang N,Xing T,Cao Y,Xiang H.The cloning and expression of renalase and the preparation of its monoclonal antibody.J Shanghai Jiaotong Univ(SCI)2009;14:376-379.

[9] Malyszko J,Zbroch E,Malyszko JS,Koc-Zorawska E,Mysliwiec M.Renalase,

a novel regulator of blood pressure,is predicted by kidney function in renal transplant recipients.Transplant Proc 2011;43:3004-3007.

[10] Przybylowski P,Malyszko J,Kozlowska S,Koc-Zorawska E,Mysliwiec M.Serum renalase,depends on kidney function but not on blood pressure in heart transplant recipients.Transplnat Proc 2011;43:3888-3891.

[11] Schlaich M,Socratous F,Eikelis N,Chopra R,Lambert G,Hennebry S.Renalase plasma levels are associated with systolic blood pressure in patients with resistant hypertension.J Hypertens 2010;28:e437.

[12] Zhao Q,Fan Z,He J,Chen S,Li H,Zhang P,Wang L,et al.Renalase gene is a novel susceptibility

Gene for essential hypertension:a two -stage association study in northern Han Chinese population.J Mol Med 2007;85:877-885.

[13] Farzaneh-Far R,Desir GV,Na B,Schiller NB,Whooley MA.A functional polymorphism in renalase(Glu37Asp)is associated with cardic hypertrophy,d -ysfunction,and ischemia:data from the heart and soul study.Plos One 2010;5:e13496.

[14] Stec A,Semczuk A,Furmaga J,Ksiazek A,Buraczynska M.Polymorphism of the renalase gene in end-stage renal disease patients affected by hypertension.Nephrol Dial Transplant 2012;27:4162-4166.

[15] Buraczynska M,Zukowski P,Buraczynska K,Mozul S,Ksiazek A.Renalase Gene polymorphisms in patients with Type 2 Diabetes,Hypertension and Storke.Neur-omol Med 2011;13:321-327.

[16] Boomsma F,Tipton KF.Renalase,a catecholamine-metabolising enzyme?J Neural Transm 2007;114:775-776.

[17] Bonaiuto E,Lunelli M,Scarpa M,Vettor R,Milan G,Di Paolo ML.A structure-activity study to identify novel and efficient substrates of the human semicarbazide-senstitive amine oxidase/VAP-1 enzyme.Biocbimie 2010;92:858-868.

[18] Noda K,Nakao S, Zandi S,Engelstadter V,Mashima Y,Hafezimoghadam A.Vascular adhesion protein-1 regulates leukocyte transmigration rate in the retina during diabetes.Exp Eye Res 2009;89:774-781.

[19] Stolen CM,Madanat R,Marti L,Kari S,Yegutkin GG,Sariola H,et al.Semicarbazide sensitive amine oxidase overexpression has dual consequences:in-sulin mimicry and diabetes-ike complications.FASEB J 2004;18:70- 2-704.

[20] Mercader J,Iffiu-Soltesz Z,Brenachot X,Foldi A,Dunkel P,Balogh B,et al.SSAO substrates exhibiting insulin-like effects ub aduoicytes as a promising

treatment option for metabolic disorders.Future Med Chem 2010;2:1735-1749.

[21] Lin SY,Wang CC,Lu YL,Wu WC,Hou WC.Antioxidant,antisemicarba -zide-senstive amine oxidase,and antihypertensive activities of geraniin isolated from phyllantbus urinaria.Food Chem Toxicol 2008;46:2485-2492.

[22] Medvedev AE,Veselovsky AV,Fedchenko VI.Renalase,a new secretory enzyme responsible for selective degradation of catecholamines:achievements and unsolved problems.Biochemistry(Mosc) 2010;75:951-958.

[23] Pandini V,Ciriello F,Tedeschi G,Rossoni G,Zanetti G,Aliverti A.Synthesis of human renalase1 in Escherichia coli and its purification as a FAD-containing holoprotein.Protein Expr Purif 2010;72:244-253.

[24] Luft FC.Renalase,a catecholamine-metabolizing hormone from the kidney.Cell Metab 2005;1:358-360.

[25] Eikelis N,Hennebry SC,Lambert GW,Schlaich MP.Does renalase degrade catecholamines?Kidney int 2011;79:1380.

[26] Milani M,Ciriello F,Barono S,Pandin V,Canevari G,Bolognesi M,Aliverti A.FAD-binding site and NADP reactivity in human renalase:a new enzyme involved in blood pressure regulation.J Mol Biol 2011;411:463-473.

[27] Eisenhofer G,Rundquist B,Aneman A,Friberg P,Dakak N,Kopin IJ,et al.Regional release and removal of catecholamines and extraneuronal metabolism to metanephrines.J Clin Endocrinol Metab 1995;80:3009-3017.

[28] Lubas A,Zelichowski G,Prochnicka A,Wisniewska M,Wankowicz Z.Renal autoregulation in medical therapy of renovascular hypertension.Arcb Med Sci 2010;6:912-918.

[29] Banach M,Kjeldsen SE,Narkiewicz K.Controversies in hypertension treatment.Curr Vasc Pharmacol 2010;8:731-732.

[30] Malyszko J,Zbroch E,Malyszko JS,Koc-Zorawska E,Mysliwiec M.Renalase was not related to blood pressure,but to residual renal function in haemodialysis and peritoneal dialysis patients.J Am Soc Nephrol 2011;22:723A.

攻读硕士学位期间主要研究成果

[1]郭运忠，李禄洪，谭丽华 去肾交感神经术对自发性高血压大鼠肾胺酶及络氨酸羟化酶的影响，中南大学学报（医学版）2012，37（8），829-833

[2]《合并心血管损害的OSAHS遗传多态性研究》定于6月见刊。

致 谢

从入学到毕业，转眼一瞬间，三年的点点滴滴历历在目，衷心感谢所以给予我帮助的人。

首先，特别感谢我最尊敬的导师蒋卫红教授，作为您的学生是我最大的荣幸。三年来的点点滴滴，对我生活和工作的指导让我铭记。多少次在我迷茫时、困顿时都是您给了我鼓励与帮助。导师严谨的科研态度、求实的工作作风以及与人交往时和善的态度，永远是我学习的榜样。

感谢唐晓鸿老师、欧阳茂老师在我刚进科室时对我临床方面的指导，使我的临床思维及专业技能方面得到了很大的提升。感谢王晓燕老师在生活及学习方面的照顾。感谢 12 病室、13 病室的所有医护人员，在我进行样本收集的时候给予了极大的便利。

感谢湘雅三医院遗传学实验室在实验环境、设备及技术上的支持。感谢王雨同学在实验流程方面的指导，感谢药理学所有可爱的师姐在统计学方面的指导。

感谢我的师兄郭运忠、师姐谭丽华的关心鼓励，以及你们对本课题的贡献。感谢我的师弟师妹们对于我生活上的帮助和关心。谢谢宿舍 320 的所有兄弟们，有你们陪伴的精彩 3 年。

最后谢谢我的父母、哥哥嫂子，在我遇到挫折时给予的帮助，你们是我的精神支柱。

愿所有爱我的、我爱的人身体健康、一生平安。我会加倍努力，希望能更多的回报你们。

肾胺酶基因多态性与高血压及合并冠心病的相关性研究

万方数据 WANFANG DATA 文献链接

作者：　李禄洪

学位授予单位：　中南大学

引用本文格式：李禄洪 肾胺酶基因多态性与高血压及合并冠心病的相关性研究[学位论文]硕士 2013
