# Supplementary material for: Association of Renalase SNPs rs2296545 and rs2576178 with the Risk of Hypertension: A Meta-Analysis
Source: PLoS One. 2016 Jul 19;11(7):e0158880. doi: 10.1371/journal.pone.0158880 (PMC4951046; doi:10.1371/journal.pone.0158880)
Supplement: S3 File — (ZIP) [file pone.0158880.s003.zip › 11 excluded records/2x Xiaogang Li et al. 2014.pdf]

Kidney & Blood Pressure Research

Kidney Blood Press Res 2014;39:9-16
DOI: 10.1159/000355771
Published online: April 30, 2014
Accepted: March 28, 2014
© 2014 S. Karger AG, Basel
www.karger.com/kbr
1423-0143/14/0391-0009$39.50/0
Karger Open access

This is an Open Access article licensed under the terms of the Creative Commons Attribution-NonCommercial 3.0 Unported license (CC BY-NC) (www.karger.com/OA-license), applicable to the online version of the article only. Distribution permitted for non-commercial purposes only.

Original Paper

Renalase Gene Polymorphism in Patients with Hypertension and Concomitant Coronary Heart Disease

Xiaogang Li Weihong Jiang Luhong Li Ruixia Huang Qiong Yang
Yan Yang Yuan Hong Xiaohong Tang

Department of Cardiovascular Medicine, The Third Xiangya Hospital of Central South University, Changsha, Hunan 410013, China

Key Words
Renalase • Genotype • Hypertension • Coronary heart disease

Abstract
Background/Aims: This study aimed to investigate renalase gene polymorphism in patients with hypertension and concomitant coronary heart disease (CHD) and to evaluate the risk for CHD in hypertensive patients from the view of genetics. ***Methods:*** NCBI and HapMap genome database were employed to screen the Single nucleotide polymorphisms (SNP). These SNPs were detected in hypertensive and CHD patients (n=791), hypertensive patients (n=802) and healthy controls (n=812), and the genotypes were recorded. Haploview 4.2 software was used to determine the genotypes, allele frequency, haplotypes, linkage disequilibrium and Hardy-Weinberg (HWE) equilibrium, and odds ratio (OR) was calculated with non-conditioned logistic regression analysis. ***Results:*** The frequency of allele A of rs2576178 in patients with hypertensive and CHD was markedly higher than that in hypertensive patients (p=0.001, OR=1.625,95% CI 1.221-2.160). The frequency of allele C of rs2296545 in hypertensive patients was significantly higher than that in healthy controls (P=0.009, OR=1.436, 95% CI 1.095-1.883). ***Conclusion:*** The allele A of rs2576178 may be a predisposing factor of CHD in hypertensive patients, and hypertensive patients with AA genotype are susceptible to develop CHD. The allele C of rs2296545 may be a predisposing factor of hypertension and patients with CC genotype are susceptible to develop hypertension.

Copyright © 2014 S. Karger AG, Basel

Introduction

Hypertension is one of common cardiovascular diseases and also a major risk factor of atherosclerosis, and plays crucial roles in the occurrence and development of coronary

Weihong Jiang

Department of Cardiovascular Medicine, The Third Xiangya Hospital of Central South University, Changsha, Hunan 410013 (China)
E-Mail jiangweihongdoc@163.com

KARGER

Downloaded by:
Xi'an Jiaotong University
198.143.44.49 - 4/20/2016 6:28:54 AM

DOI: 10.1159/000355771
Published online: April 30, 2014
© 2014 S. Karger AG, Basel
www.karger.com/kbr

heart diseases (CHD) [1]. It has been shown that the amplitude of the increase in blood pressure is linearly correlated with the incidence of CHD [2]. Renalase is a flavin adenine dinucleotide dependent amine oxidase and secreted in the kidney. Renalase is involved in the regulation of blood pressure and cardiac function via degrading catecholamines in the blood circulation [3, 4]. Human renalase gene is mapped to 10q23.33 [5] and highly expressed in the kidney and heart [6, 7]. To date, available studies focus on the role of renalase gene polymorphism in the hypertension, and the single nucleotide polymorphisms (SNP) studied in previous studies is usually localized at the potential functional domains. There is evidence showing that SNP of rs2296545 is associated with hypertension [8] or hypertension and concomitant diabetes [9]. In addition, the SNP of rs2576178 was also found to be related to the pathogenesis of hypertension [8] or the pathogenesis of hypertension in patients with end stage renal disease (ESRD) [10]. However, the SNP of rs10887800 has been confirmed to be associated with the atherosclerosis and the stroke in hypertensive patients [9].

Our previous study confirmed that renalase was involved in the regulation of blood pressure in spontaneously hypertensive rats (SHR) [11]. In addition, studies also confirmed that renalase participated in the protection against myocardial ischemia in CHD [12]. This study was undertaken to analyze the SNP of renalase gene in hypertensive patients and those with hypertension and concomitant CHD, aiming to investigate the association of renalase gene with hypertension and concomitant CHD. Our findings may provide evidence to identify hypertensive patients who are susceptible to develop CHD and to find new way to predict CHD from the view of genetics.

Materials and Methods

Subjects

Inpatients were recruited from the Department of Cardiology of the Third Xiangya Hospital of Central South University and healthy controls who received physical examination were from Health Management Center from August 2012 to November 2013. A total of 2405 subjects were recruited into present study. There 802 patients with hypertension alone, 791 patients with both hypertension and CHD and 812 healthy controls.

Inclusion criteria

Hypertension was diagnosed according to the Guideline for the Prevention and Treatment of Hypertension in China (2010). All the patients were Han Chinese and aged 45-65 years. The hypertensions of these patients were all at mild-to-moderate degrees (140-160/90-100 mmHg). Long effective angiotensin-converting enzyme inhibitors or (and) calcium channel blockers were used to control the blood pressure. The ratio of males to females was acceptable. The course of hypertension was longer than 10 years.

CHD was diagnosed when there was definite history of vascular stenosis (coronary angiography) or myocardial infarction.

Following patients were excluded: secondary hypertension; patients with other heart diseases (myocarditis, cardiomyopathy, pericarditis, valvular heart disease); patients with diabetes or chronic kidney diseases; patients with cerebral infarction; patients with peripheral vascular diseases; patients with autoimmune diseases; patients with tumors. Based on the inclusion criteria in former studies, we excluded the diabetes mellitus and peripheral vascular diseases, in order to prevent the impacts of atherosclerosis related risk factors or diseases on gene polymorphism phenotypes. We chose the simple hypertension patients at the age of 45-65 years old as the research objects, in order to exclude the young secondary hypertension patients or elder hypertension patients with undiagnosed atherosclerosis.

Samples and data collection

Venous blood (2 ml) was collected and anti-coagulated with EDTA. Then, plasma and blood cells were separated by centrifugation at 3000 r/min for 15 min and stored at -80°C. The demographics (such as gender, age, smoking and drinking status, body mass index [BMI] was recorded. On admission, the blood sodium (Na), renal function (Creatinine, Cr), blood glucose (Glu), and blood lipids (such as total cholesterol

KARGER
Downloaded by:
Xi'an Jiaotong University
198.143.44.49 - 4/20/2016 6:28:54 AM

[TC], triglycerides [TG], high-density lipoprotein cholesterol [HDL-C], low-density lipoprotein cholesterol [LDL-C]) were determined (in the Department of Laboratory of the Third Xiangya Hospital of Central South University).

Screening of SNP

The renalase gene data of Han Chinese in Beijing was screened in the NCBI and HapMap. Haploview4.2 software was used to screen tag SNPs related to the present study. Selection criteria for SNPs: 1) Minor allele frequency of SNP is no lower than 0.05; 2) The SNP with positive findings and high influence factor is preferred.

Identification of genotype

DNA extraction was performed with a DNA extraction kit (QIAGEN, Germany). Detection of SNPs were as follows: 1) Primers used for PCR: Primers for rs2576178: 5'AGC AGA GAA GCA GCT TAA CCT 3' (forward), 5'TTA TCT GCA AGT CAG CGT AAC3' (reverse); primers for rs2296545: 5'GGA AGT CCC CGA TCA CGT GAC3' (forward), 5'TGC TGT GTG GGA CAA GGC TGA3' (reverse). Primers were synthesized in the Nanjing BioSky Sci & Tech Co., Ltd. 2) PCR was done in a 50-μl mixture and the conditions were as follows: predenaturation at 95°C for 10 min, denaturation at 95°C for 15 s, and annealing and extension at 60°C for 1 min. Products from PCR were stored at 4°C. 3) Digestion: After PCR, 10 μl of products were subjected to digestion with restriction endonuclease (5 U; MspI for rs2576178 and Eco81I for rs2296545; Nanjing BioSky Sci & Tech Co., Ltd) at 37°C for 10 h. 3) Following preparation of 1.5% gel, the products after digestion were subjected to electrophoresis. 4) Image analysis was performed with GD8000 imager (UVP, USA).

Statistical analysis

SPSS 19.0 and Haploview 4.2 were used for statistical analysis. Quantitative data were expressed as mean ± standard deviation (x±s). Comparisons among groups were done with Kruskal-Wallis test. Qualitative data were expressed as percentage (%) or specific values, and comparisons were performed chi square test. Haploview software was used for genotype analysis, analysis of allele frequency, HWE test and analysis of linkage disequilibrium (LD) of SNPs (D and r^2 represent the degree of LD of SNPs) and analysis of haplotype. The association of renalase gene SNPs with hypertension and concomitant CHD was evaluated by odd risk and 95% confidence interval (CI) which were calculated with non-conditioned logistic regression analysis. A value of $P<0.05$ was considered statistically significant.

Results

In the present study, genotype, allele and haplotype were analyzed in rs2576178 and rs2296545, aiming to elucidate the association of renalase with hypertension and hypertension combined with CHD.

Analysis of demographics

There were no significant differences in the gender, age, smoking and drinking status among three groups. However, significant difference was observed in BMI between patients and healthy controls. In addition, the levels of Na and TG were comparable among three groups, and remarkable differences were noted in Glu, TC, LDL-C, HDL-C and Cr. The levels of Glu, TC and LDL-C were the highest in patients with hypertension and CHD and the lowest in healthy controls. On the contrary, the HDL-C was highest in healthy controls and the lowest in patients with hypertension and CHD (Table 1). In the present study, after digestion of rs2576178 and rs2296545 with corresponding restriction endonuclease, the length of products was 525 bp for allele A and 423bp+102bp for allele G, and 188 bp+21bp for allele C and 209 bp for allele G, respectively (Figure 1).

HWE test and LD analysis of renalase gene SNP

Hardy-Weinberg genetic equilibrium states that allele and genotype frequencies in a population will remain constant from generation to generation in the absence of other

KARGER
Downloaded by:
Xi'an Jiaotong University
198.143.44.49 - 4/20/2016 6:28:54 AM

DOI: 10.1159/000355771
Published online: April 30, 2014
© 2014 S. Karger AG, Basel
www.karger.com/kbr

Table 1. Demographics and biochemical parameters of subjects in three groups

Variables	① (n=791)	② (n=802)	③ (n=812)	P
Gender (M/F)	102/89	107/95	109/123	P>0.05
Age (yr)	56.4±9.7	54.8±8.9	57.3±10.2	P>0.05
BMI (kg/m^2)	26.53±3.71	25.98±3.41	23.02±3.17	P<0.05
GLU (mmol/L)	6.51±1.76	5.98±1.69	5.37±1.28	P<0.05
TG (mmol/L)	1.68±0.89	1.71±1.02	1.72±0.96	P>0.05
TC (mmol/L)	5.35±0.84	5.21±0.99	4.78±1.01	P<0.05
HDL-C (mmol/L)	1.19±0.32	1.22±0.31	1.39±0.42	P<0.05
LDL-C (mmol/L)	4.21±0.97	3.17±1.16	2.71±1.24	P<0.05
Cr (μmol/L)	70.29±13.27	74.27±15.21	66.15±11.29	P<0.05
Na (mmol/L)	139.27±4.28	140.19±5.11	ND	P>0.05
Smoker (%)	41.5%	41.7%	39.8%	P>0.05
Drinker (%)	29.3%	31.0%	30.1%	P>0.05

Note: ① HBP+CHD group: patients with hypertension and CHD; ② HBP group: hypertensive patients; ③ healthy controls

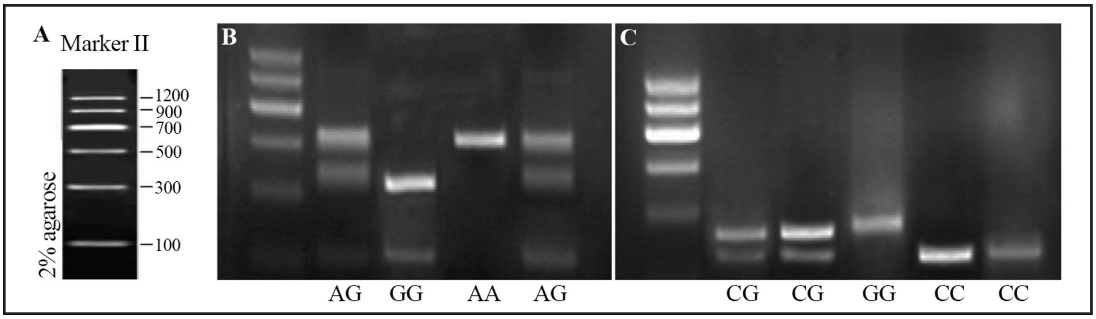

Fig. 1. Electrophoresis of rs2576178 and rs2296545. **(A)**: Marker used in electrophoresis. After digestion of rs2576178 **(B)** and rs2296545 **(C)** with corresponding restriction endonuclease, the length of products was 525 bp for allele A and 423bp+102bp for allele G, and 188 bp+21bp for allele C and 209 bp for allele G, respectively.

evolutionary influences (such as natural selection, mutation and production of new genes). Haploview4.2 software was used for HWE test. The free degree of 1 and P of >0.05 represent the HWE. In the present study, HWE test was done in three groups, and results showed 2 SNPs in three groups met HWE, suggesting that the subjects investigated in the present study were from the general population and representative.

LD is also known as allelic association and refers to the non-random association of alleles at two or more loci that descend from single, ancestral chromosomes. LD is caused by gene mutation or gene recombination. In the present study, LD analysis of Rs2576178 and Rs2296545 showed the D/r^2 ratio was 0.040/0.001, suggesting the low degree of LD at two sites and a low mutual predictive value. Further haplotype analysis is required [13].

Correlation analysis of renalase gene among three groups

The genotypes and allele frequency of rs2576178 of hypertension patients with CHD, hypertensive patients and controls are shown in Table 2. Results showed marked differences in the genotypes and allele frequency. After comparisons between two groups, no significant difference in the genotypes and allele frequency of rs2576178 were observed between hypertensive patients and controls (P=0.688 and 0.979). However, remarkable differences in the genotypes and allele frequency of rs2576178 were noted between hypertensive patients with CHD and hypertensive patients. The proportion of patients with AA genotype and the allele A frequency in hypertensive patients with CHD were significantly higher than those in hypertensive patients (P>0.001 for both). The OR of allele A was 1.625, and 1 was not included in the 95% CI, suggesting the marked difference in the risk for allele A.

KARGER
Downloaded by:
Xi'an Jiaotong University
198.143.44.49 - 4/20/2016 6:28:54 AM

DOI: 10.1159/000355771
Published online: April 30, 2014
© 2014 S. Karger AG, Basel
www.karger.com/kbr

Table 2. Genotypes and allele frequency of rs2576178 and rs2296545 in three groups

Groups / SNP	① (n=791)	② (n=802)	③ (n=812)	P ①②	P ②③	P ①③	P ①②③
**rs2576178**							
AA	76(0.398)	47(0.233)	59(0.254)	0.001	0.688	0.003	0.031
AG	88(0.461)	112(0.554)	119(0.513)				
GG	27(0.141)	43(0.213)	54(0.233)				
Allele A	240(0.628)	206(0.510)	237(0.511)	0.001	0.979	0.001	0.017
Allele G	142(0.372)	198(0.490)	227(0.489)				
HWE test	X^2=0.035405	X^2=2.415218	X^2=0.160946				
	P=0.851	P=0.120	P=0.688				
OR (95%CI)	①②	②③	①③				
	1.625 [1.221-2.160]	0.997 [0.763-1.301]	1.619 [1.228-2.133]				
**rs2296545**							
CC	74(0.387)	78(0.386)	67(0.289)	0.363	0.037	0.363	0.179
CG	78(0.408)	93(0.460)	111(0.478)				
GG	39(0.204)	31(0.153)	54(0.233)				
Allele C	226(0.592)	249(0.616)	245(0.528)	0.479	0.009	0.479	0.082
Allele G	156(0.408)	155(0.384)	219(0.472)				
HWE test	X^2=3.580919	X^2=0.147919	X^2=0.372862				
	P=0.062	P=0.706	P=0.541				
OR (95%CI)	①②	②③	①③				
	0.902 [0.677-1.200]	1.436 [1.095-1.883]	0.902 [0.677-1.200]				

Note: ① HBP+CHD group: patients with hypertension and CHD; ② HBP group: hypertensive patients; ③ healthy controls

Similar findings were observed between hypertensive patients with CHD and controls. The proportion of patients with AA genotype and the allele A frequency in hypertensive patients with CHD were significantly higher than those in controls, and allele A carriers had a high risk (OR=1.619).

The genotypes and allele frequency of rs2296545 of hypertension patients with CHD, hypertensive patients and controls are shown in Table 2. Results showed there were no marked differences in the genotypes and allele frequency of rs2296545 among three groups. Comparisons between two groups showed the proportion of patients with CC genotype and allele C frequency in hypertensive patients were markedly higher than those in controls (P=0.037 and 0.009). The OR of allele C was 1.436. However, no remarkable differences were observed in the genotypes and allele frequency of rs2296545 between hypertensive patients with CHD and hypertensive patients and between hypertensive patients with CHD and controls.

Haplotype analysis of renalase

Haplotype analysis can not only evaluate the association of haplotype with relevant diseases, but also reflect the susceptibility gene related to a specific disease after comprehensive analysis because it integrates the genetic information of multiple sites [14]. In the present study, two SNPs of renalase gene were analyzed, and results showed a low degree of LD. Further haplotype analysis was performed in which the haplotype with the frequency of <0.05 was neglected among 4 haplotypes. Finally, 3 haplotypes were included for haplotype analysis (Table 3). Results showed the frequency of haplotype G-C in hypertensive patients was significantly higher than that in controls, and haplotype G-C contains the allele C of rs2296545. The frequency of haplotype A-C in hypertensive patients with CHD was markedly higher than that in patients with hypertension alone, and this haplotype contains the allele A of rs2576178. The frequency of haplotype A-G in hypertensive patients with CHD was markedly higher than that in controls and this haplotype also contains the allele A of rs2576178.

Genotypes, general clinical information and biochemical parameters

On the basis of genotypes of rs2576178 (AA, AG, GG) and rs2296545 (CC, GG, GC) of renalase gene, patients were classified, and the general clinical information and bio-

KARGER
Downloaded by:
Xi'an Jiaotong University
198.143.44.49 - 4/20/2016 6:28:54 AM

chemical parameters were compared among groups with different genotypes. Results showed there were no significant differences in the general information and biochemical parameters among groups with different genotypes for both SNPs (P>0.05)

Table 3. Distribution of different haplotypes in three groups

Haplotype \ Results	Frequency		X^2	P	OR [95%CI]
② and ③	②	③			
A-G	0.090	0.059	3.008	0.083	1.571 [0.940-2.625]
A-C	0.163	0.124	2.576	0.109	1.366 [0.932-2.001]
G-C	0.135	0.082	6.336	0.012	1.745 [1.127-2.703]
① and ②	①	②			
A-G	0.128	0.090	2.879	0.090	1.479 [0.939-2.328]
A-C	0.233	0.163	6.167	0.013	1.565 [1.097-2.812]
G-C	0.088	0.135	4.420	0.036	0.616 [0.390-0.971]
① and ③	①	③			
A-G	0.128	0.059	11.968	0.001	2.322 [1.425-3.783]
A-C	0.233	0.124	17.238	NS	NS
G-C	0.088	0.082	0.084	0.772	1.074 [0.661-1.746]

Note: ① HBP+CHD group: patients with hypertension and CHD; ② HBP group: hypertensive patients; ③ healthy controls. NS: not significant

Discussion

Hypertension as the most common chronic disease has been paid attention to due to a high morbidity and high risk for damage to the target organs. Hypertension is the most common risk factor of cardiovascular and cerebrovascular diseases and closely related to the CHD and brain stroke. Gene polymorphism analysis in case-control studies has found that renalase gene is related to several diseases in humans. Renalase gene has been found to be involved in the regulation of blood pressure [15]. Further studies reveal that renalase is closely associated with hypertension, myocardial ischemia [12], acute kidney injury [16], diseases related to organ transplantation [17-19], heart dysfunction [20, 21], ischemic stroke [9, 22] and diabetes [23, 24]. The two SNPs of renalase gene (rs2576178 and rs229654) have been found to be related to hypertension [8]. Of two SNPs, the allele G frequency of rs2576178 and the allele C frequency of rs2296545 are closely related to hypertension. In addition, there is evidence [9, 10] showing that the allele C frequency of rs2296545 of renalase gene might be associated with diabetes.

Hypertension is a risk factor of CHD, and it relevant susceptibility genes might be risk factors of CHD. As known to all, renalase gene expression is related to renal function. Renal functions of patients in this study were within normal range. This study was designed to investigate the association of hypertension or CHD with renalase gene expression in patients with normal renal function. In the present study, case-control association analysis was performed in hypertensive patients with CHD, patients with hypertension alone and healthy controls. We investigated the association of renalase gene SNPs with hypertension combined with CHD. The genotypes and allele frequency were compared among groups. Results showed the allele A frequency of rs2576178 in hypertensive patients with CHD was markedly higher than that in hypertensive patients and controls, and marked difference was also observed in the genotypes. However, there were no remarkable differences between hypertensive patients and controls. These suggest that allele A of rs2576178 is a susceptibility factor of CHD, and allele G a protective factor. In addition, the allele C frequency of rs2296545 in hypertensive patients was significantly higher than that in controls, and the proportion of patients with CC genotype in hypertensive patients was markedly higher, but no remarkable differences were observed in any two groups. These suggest that the allele C of rs2296545 is a susceptibility factor of hypertension and allele G a protective factor. Haplotype analysis showed marked differences were observed in haplotype G-C between hypertensive patients and controls which contains allele C of rs2296545, in haplotype A-C between patients

KARGER
Downloaded by:
Xi'an Jiaotong University
198.143.44.49 - 4/20/2016 6:28:54 AM

with hypertension alone and hypertensive patients with CHD which contains allele A of rs2576178. These findings further demonstrate that these two SNPs are related to diseases.

In the present study, to avoid false positive due to population stratification and mixing, only Han Chinese from South China were recruited. Thus, objectively, subjects in our study might have difference in renalase gene SNP with population of different races and in different areas. Our findings demonstrate the renalase gene is associated with the susceptibility to hypertension. In addition, the presence of hypertension and concomitant CHD might be related to renalase. To analyze and discus from the view of etiology is logical to elucidate this correlation. rs2576178 of renalase gene is localized at the 5‘ wing of renalase gene, and rs2296545 in the exon 2 of renalase gene. rs2576178 may influence the initiation of transcription and rs2296545 might change the gene functions via influencing the structure or functions of proteins. However, the ways and mechanisms by which renalase influences the blood pressure and myocardial ischemia to play a role in diseases are still poorly understood [25-27].

There were limitations in this study. This study was conducted in Han Chinese from South China and had a small sample size. The exact association of hypertension and concomitant CHD with renalase gene is required to be elucidated and validated in studies with large sample size. There are no findings from functional studies to support whether two SNPs investigated in the present study influence the functions of renalase gene. Thus, more functional studies are required to confirm the association of renalase with hypertension and concomitant CHD.

Disclosure Statement

There is no conflict of interest to declare.

Acknowledgements

This study was supported by the Natural Science Foundation of Hunan Province (14JJ2035), Fundation for Key Disciplines of the Third Xiangya Hospital of Central South University and Special Funds for Fundamental Research in Central Universities (2013zzts344).

References

1 Olafiranye O, Zizi F, Brimah P, Jean-Louis G, Makaryus AN, McFarlane S, Ogedegbe G: Management of Hypertension among Patients with Coronary Heart Disease. Int J Hypertens 2011;2011:653903.
2 Lewington S, Clarke R, Qizilbash N, Peto R, Collins R: Age-specific relevance of usual blood pressure to vascular mortality: a meta-analysis of individual data for one million adults in 61 prospective studies. Lancet 2002;360:1903-1913.
3 Milani M, Ciriello F, Baroni S, Pandini V, Canevari G, Bolognesi M, Aliverti A: FAD-binding site and NADP reactivity in human renalase: a new enzyme involved in blood pressure regulation. J Mol Biol 2011;411:463-473.
4 Zbroch E, Koc-Zorawska E, Malyszko J, Malyszko J, Mysliwiec M: Circulating levels of renalase, norepinephrine, and dopamine in dialysis patients. Ren Fail 2013;35:673-679.
5 Xu J, Li G, Wang P, Velazquez H, Yao X, Li Y, Wu Y, Peixoto A, Crowley S, Desir GV: Renalase is a novel, soluble monoamine oxidase that regulates cardiac function and blood pressure. J Clin Invest 2005;115:1275-1280.

KARGER
Downloaded by:
Xi'an Jiaotong University
198.143.44.49 - 4/20/2016 6:28:54 AM

6 Hennebry SC, Eikelis N, Socratous F, Desir G, Lambert G, Schlaich M: Renalase, a novel soluble FAD-dependent protein, is synthesized in the brain and peripheral nerves. Mol Psychiatry 2010;15:234-236.
7 Fedchenko V, Globa A, Buneeva O, Medvedev A: Renalase mRNA levels in the brain, heart, and kidneys of spontaneously hypertensive rats with moderate and high hypertension. Med Sci Monit Basic Res 2013;19:267-270.
8 Zhao Q, Fan Z, He J, Chen S, Li H, Zhang P, Wang L, Hu D, Huang J, Qiang B, Gu D: Renalase gene is a novel susceptibility gene for essential hypertension: a two-stage association study in northern Han Chinese population. J Mol Med (Berl) 2007;85:877-885.
9 Buraczynska M, Zukowski P, Buraczynska K, Mozul S, Ksiazek A: Renalase gene polymorphisms in patients with type 2 diabetes, hypertension and stroke. Neuromolecular Med 2011;13:321-327.
10 Stec A, Semczuk A, Furmaga J, Ksiazek A, Buraczynska M: Polymorphism of the renalase gene in end-stage renal disease patients affected by hypertension. Nephrol Dial Transplant 2012;27:4162-4166.
11 Jiang W, Guo Y, Tan L, Tang X, Yang Q, Yang K: Impact of renal denervation on renalase expression in adult rats with spontaneous hypertension. Exp Ther Med 2012;4:493-496.
12 Wu Y, Xu J, Velazquez H, Wang P, Li G, Liu D, Sampaio-Maia B, Quelhas-Santos J, Russell K, Russell R, Flavell RA, Pestana M, Giordano F, Desir GV: Renalase deficiency aggravates ischemic myocardial damage. Kidney Int 2011;79:853-860.
13 Barrett JC, Fry B, Maller J, Daly MJ: Haploview: analysis and visualization of LD and haplotype maps. Bioinformatics 2005;21:263-265.
14 Lake SL, Lyon H, Tantisira K, Silverman EK, Weiss ST, Laird NM, Schaid DJ: Estimation and tests of haplotype-environment interaction when linkage phase is ambiguous. Hum Hered 2003;55:56-65.
15 Desir GV, Tang L, Wang P, Li G, Sampaio-Maia B, Quelhas-Santos J, Pestana M, Velazquez H: Renalase lowers ambulatory blood pressure by metabolizing circulating adrenaline. J Am Heart Assoc 2012;1:e002634.
16 Lee HT, Kim JY, Kim M, Wang P, Tang L, Baroni S, D'Agati VD, Desir GV: Renalase protects against ischemic AKI. J Am Soc Nephrol 2013;24:445-455.
17 Koc-Zorawska E, Malyszko J, Malyszko JS, Mysliwiec M: VAP-1, a novel molecule linked to endothelial damage and kidney function in kidney allograft recipients. Kidney Blood Press Res 2012;36:242-247.
18 Przybylowski P, Malyszko J, Kozlowska S, Malyszko J, Koc-Zorawska E, Mysliwiec M: Serum renalase depends on kidney function but not on blood pressure in heart transplant recipients. Transplant Proc 2011;43:3888-3891.
19 Zbroch E, Malyszko J, Malyszko J, Koc-Zorawska E, Mysliwiec M: Renalase, kidney function, and markers of endothelial dysfunction in renal transplant recipients. Pol Arch Med Wewn 2012;122:40-44.
20 Gu R, Lu W, Xie J, Bai J, Xu B: Renalase deficiency in heart failure model of rats--a potential mechanism underlying circulating norepinephrine accumulation. PLoS One 2011;6:e14633.
21 Ghosh SS, Krieg RJ, Sica DA, Wang R, Fakhry I, Gehr T: Cardiac hypertrophy in neonatal nephrectomized rats: the role of the sympathetic nervous system. Pediatr Nephrol 2009;24:367-377.
22 Zhang R, Li X, Liu N, Guo X, Liu W, Ning C, Wang Z, Sun L, Fu S: An association study on renalase polymorphisms and ischemic stroke in a Chinese population. Neuromolecular Med 2013;15:396-404.
23 Barrett JC, Clayton DG, Concannon P, Akolkar B, Cooper JD, Erlich HA, Julier C, Morahan G, Nerup J, Nierras C, Plagnol V, Pociot F, Schuilenburg H, Smyth DJ, Stevens H, Todd JA, Walker NM, Rich SS: Genome-wide association study and meta-analysis find that over 40 loci affect risk of type 1 diabetes. Nat Genet 2009;41:703-707.
24 Reddy MV, Wang H, Liu S, Bode B, Reed JC, Steed RD, Anderson SW, Steed L, Hopkins D, She JX: Association between type 1 diabetes and GWAS SNPs in the southeast US Caucasian population. Genes Immun 2011;12:208-212.
25 Pandini V, Ciriello F, Tedeschi G, Rossoni G, Zanetti G, Aliverti A: Synthesis of human renalase1 in Escherichia coli and its purification as a FAD-containing holoprotein. Protein Expr Purif 2010;72:244-253.
26 Eikelis N, Hennebry SC, Lambert GW, Schlaich MP: Does renalase degrade catecholamines? Kidney Int 2011;79:1380; author reply 1380-1381.
27 Lubas A, Zelichowski G, Prochnicka A, Wisniewska M, Wankowicz Z: Renal autoregulation in medical therapy of renovascular hypertension. Arch Med Sci 2010;6:912-918.

KARGER
Downloaded by:
Xi'an Jiaotong University
198.143.44.49 - 4/20/2016 6:28:54 AM
