# Supplementary material for: Association of Renalase SNPs rs2296545 and rs2576178 with the Risk of Hypertension: A Meta-Analysis
Source: PLoS One. 2016 Jul 19;11(7):e0158880. doi: 10.1371/journal.pone.0158880 (PMC4951046; doi:10.1371/journal.pone.0158880)
Supplement: S3 File — (ZIP) [file pone.0158880.s003.zip › 11 excluded records/2x Yang Wang et al. 2014.pdf]

Kidney & Blood Pressure Research

Kidney Blood Press Res 2014;39:497-506
DOI: 10.1159/000368460
Published online: November 26, 2014
Accepted: September 30, 2014
© 2014 S. Karger AG, Basel
www.karger.com/kbr
1423-0143/14/0395-0497$39.50/0
Karger Open access

This is an Open Access article licensed under the terms of the Creative Commons Attribution-NonCommercial 3.0 Unported license (CC BY-NC) (www.karger.com/OA-license), applicable to the online version of the article only. Distribution permitted for non-commercial purposes only.

Original Paper

Genetic Variants in Renalase and Blood Pressure Responses to Dietary Salt and Potassium Interventions: A Family-Based Association Study

Yang Wang Chao Chu Jie Ren Jian-Jun Mu Dan Wang Fu-Qiang Liu Ke-Yu Ren Tong-Shuai Guo Zu-Yi Yuan

Department of Cardiovascular Medicine, First Affiliated Hospital of Medical College, Xi'an Jiaotong University and Key Laboratory of Environment and Genes Related to Diseases, Ministry of Education, Xi'an, P. R. China

Key Words
Renalase • Salt • Potassium • Gene polymorphism • Blood pressure

Abstract
Background/Aims: Renalase (gene name *RNLS*), a recently discovered enzyme with monoamine oxidase activity, is implicated in the degradation of catecholamines. Recent studies indicate that common variations in the gene with *RNLS* are associated with hypertension. The aim of this study was to examine the association between genetic variants in *RNLS* and blood pressure (BP) responses to strict dietary interventions of salt and potassium intake. ***Methods:*** A total of 334 subjects from 124 families were selected and sequentially maintained on a low-salt diet for 7 days (3.0 g/day, NaCl), then a high-salt diet for 7 days (18.0 g/day, NaCl), high-salt diet with potassium supplementation for another 7 days (4.5 g/day, KCl). ***Results:*** SNPs rs919115 and rs792205 of the *RNLS* gene were significantly associated with diastolic BP (DBP) and mean arterial pressure (MAP) responses to high-salt intervention. In addition, rs12356177 was significantly associated with systolic BP (SBP) and DBP responses to low-salt diet, and SBP, DBP or MAP during the high-salt intervention. Unfortunately, no associations for the 7 *RNLS* SNPs with BP response to high-salt diet with potassium supplementation reached nominal statistical significance. ***Conclusions:*** This family-based study indicates that genetic variants in the *RNLS* gene are significantly associated with BP responses to dietary salt intake.

Copyright © 2014 S. Karger AG, Basel

Y. Wang, C. Chu and J. Ren contributed equally to this work and thus share first authorship

Jianjun Mu, PhD

Department of Cardiovascular Medicine, First Affiliated Hospital of Medical College, Xi'an Jiaotong University, No. 277, Yanta West Road, Xi'an ,710061 (P. R. China)
Tel. +86-29-85323804, E-Mail mujjun@163.com

KARGER

Downloaded by:
Xi'an Jiaotong University
198.143.53.33 - 4/20/2016 6:34:59 AM

Introduction

Hypertension is a major global health challenge because of its high prevalence and the concomitant increase in risk of vascular disease [1, 2]. As a complex trait, hypertension susceptibility is influenced by the interaction of multiple genetic and environmental factors [3]. Among environmental determinants, high dietary salt and low potassium intake are the most important risk factors for hypertension [4, 5]. High dietary salt and low potassium intake have been associated with an increased blood pressure (BP) in animal experiments, observational epidemiologic studies, and randomized controlled clinical trials [6-8]. However, there is substantial scientific evidence suggesting that BP responses to dietary salt or potassium intake vary considerably among individuals [8, 9]. The variation in salt or potassium sensitivity of BP might be partially determined by an individual's genetic predisposition. Thus, identifying the genetic variants underlying BP response to salt and potassium intake will enhance our understanding of biological mechanisms of BP regulation and may help the development of targeted prevention and treatment strategies for hypertension.

Renalase, a 342-amino acid protein with a calculated molecular mass of 38kDa, is encoded by the *RNLS* gene on chromosome10 at q23.33 [10]. This protein is a newly discovered monoamine oxidase enzyme originating from renal tissues. It degrades circulating catecholamines and plays an important role in BP regulation [10]. Renalase has wide representation in kidney and extrarenal sites such as heart, skeletal muscle and small intestine, and is emerging as an important regulator of cardiovascular homeostasis [11]. Renalase deficiency is reportedly associated with ischemic myocardial damage, ventricular arrhythmias, heart failure, and hypertension [12]

Recently, researchers have tried to identify common variants of *RNLS* gene that may influence the risk of essential hypertension. Zhao et al. [13] firstly showed that in the Han Chinese population, the renalase encoding gene was a novel susceptibility gene for essential hypertension and its genetic variations might influence BP. Renalase gene polymorphisms have also been showed to be correlated with hypertension in patients on hemodialysis and in patients with type 2 diabetes [14, 15]. However, none of these studies took into account the gene-environment interactions, especially regarding dietary salt or potassium, on BP. Failure to measure gene and environment interactions may result in the inability to fully detect genetic contribution to BP variability in those studies. Therefore, the aim of this study was to examine the association between genetic variants in *RNLS* gene and BP responses to the dietary salt and potassium interventions among 124 Chinese families.

Materials and Methods

Study Population

In Northern China, probands with a mean systolic BP (SBP) of 130-160 mmHg and/or diastolic BP (DBP) of 85-100 mmHg were identified by community-based BP screening among all 18-60 years in the study villages. Both 2-generation (probands, their parents and siblings) and 3-generation (additionally including the probands' spouse and offspring) families were recruited for the study. Probands, along with their siblings, spouses, and offspring participated in the dietary intervention. The exclusion criteria were stage 2 hypertension; secondary hypertension; a history of clinical cardiovascular disease, chronic kidney disease, or diabetes; use of antihypertensive medication; pregnancy; high alcohol intake.

The institutional ethics committee of Xi'an Jiaotong University Medical School approved the study protocol. Written informed consents for the baseline observation and for the intervention program were obtained from each participant. This study adheres to the principles of the Declaration of Helsinki, and all studies procedures were performed in accordance with institutional guidelines.

KARGER
Downloaded by:
Xi'an Jiaotong University
198.143.53.33 - 4/20/2016 6:34:59 AM

Dietary Intervention

The chronic salt intake and potassium supplementation intervention protocol was performed as previously described [16, 17]. The protocol comprised a questionnaire survey and physical examination (height, weight, waist circumference, and BP measurements) during a 3-day baseline observation period, a low-salt diet for 7 days (3 g of salt or 51.3 mmol of sodium per day), a high-salt diet for 7 days (18 g of salt or 307.8 mmol of sodium per day), and a high-salt diet with potassium supplementation for 7 days (18 g of salt or 307.8 mmol of sodium + 60 mmol of potassium per day). During the baseline period, each subject was given detailed dietary instructions to avoid table salt, cooking salt and high-salt foods for the 21-day study duration. To ensure study participants' compliance with the intervention program, they were required to have their breakfast, lunch, and dinner at the study kitchen under supervision of the study staff during the entire study period. All foods were cooked without salt. Onsite study staff members added prepackaged salt to the individual subjects' meals as indicated by the study protocol.

BP Measurement and Defnition of BP Response to Dietary Intervention

Three random-zero BP measurements were obtained using a Hawksley random-zero sphygmomanometer (Hawksley & Sons Ltd, Lancing, UK; zero range 0–20 mmHg) with a 1-minute interval. BP was measured by trained and certified observers during the 3-day baseline observation period as well as on days 5, 6, and 7 of each of the three 7-day intervention periods according to a common protocol adapted from procedures recommended by the American Heart Association. The subjects were instructed to avoid alcohol, cigarette smoking, coffee/tea, and exercise for at least 30 min prior to their BP measurement. BP observers were blinded to the participants' dietary interventions. SBP and DBP were determined as the first and fifth Korotkoff sounds, respectively. The pulse pressure was calculated as SBP – DBP. The mean arterial pressure (MAP) was calculated as DBP + (1/3 × pulse pressure). The BP at baseline and during the intervention was calculated as the mean of nine measurements from three clinical visits during the 3-day baseline observation period and the mean of the measurements on days 5, 6, and 7 of each of the three 7-day intervention periods, respectively. BP responses were defined as follows: BP response to low-salt = BP on low-salt diet – BP at baseline; BP response to high salt = BP on high-salt diet – BP on low-salt diet; and BP response to potassium supplement = BP on high-salt with potassium supplementation – BP on high-salt diet.

Twenty-four-hour Urinary Salt and Potassium Determination

Twenty-four-hour urine samples were collected at baseline and on day 7 of each intervention period. The samples were kept frozen at –40ºC until analysis. The sodium and potassium concentrations in the urine were measured on fame photometry. The 24-h urinary excretions of sodium and potassium were calculated by multiplying the concentration of sodium and potassium, respectively, by the 24-h total urine volume.

SNP Selection and Genotyping

We selected 7 tagSNPs from *RNLS* gene, each with a minor allele frequency (MAF) > 5% in the HapMap Chinese Han Beijing population. Peripheral venous blood was drawn from each participant. Genomic DNA was isolated from whole blood samples using the GoldMag-Mini Purification Kit (GoldMag Co. Ltd. Xian, China). DNA concentration was measured using the NanoDrop 2000 (Thermo Scientific, Waltham, Massachusetts, USA). All the genotyping experiments were done by Shaanxi Lifegen Co. Ltd (available at http:// www.lifegen.com/). Genotyping was performed using MassARRAY high-throughput DNA analysis (Sequenom, San Diego, CA, USA) with matrix-assisted laser desorption/ionization time-of-light mass spectrometry techniques, according to the manufacturer's instructions. The primers were designed by MassARRAY Assay Design software (version 3.0). SNPs were genotyped using iPLEX Gold technology (Sequenom) followed by an automated data analysis with the TYPERRT software version 4.0.

Statistical analyses

The Mendelian consistency of the SNP genotype data was assessed by PLINK and PedCheck on parental SNP data. Hardy-Weinberg equilibrium was tested with chi-square test on parental SNP data. We used Haploview (version 4.1, http://www.broad.mit.edu/mpg/haploview) to estimate the extent of pairwise linkage disequilibrium between SNPs. We used Family-Based Association Test (FBAT; version

KARGER
Downloaded by:
Xi'an Jiaotong University
198.143.53.33 - 4/20/2016 6:34:59 AM

Table 1. Baseline characteristics and BP response to sodium and potassium supplementation of participants

	Probands	Siblings	Spouses	Offspring	Parents
No. of participants	99	167	19	49	181
Age (years)	41.8±8.4	39.8±7.4	47.4±6.1	23.3±6.9	66.1±8.3
Male (%)	69.7	49.1	26.3	49.0	48.4
Body mass index (kg/m ^{2})	23.0±2.8	22.2±2.9	23.1±4.7	20.1±2.7	20.4±2.6
BP at baseline (mmHg)					
Systolic	120.9±12.5	107.6±11.1	108.6±12.2	102.7±10.7	123.2±21.3
Diastolic	79.0±8.3	70.1±8.1	70.6±6.9	63.4±8.9	70.5±10.5
Mean arterial pressure	93.0±9.0	82.6±8.7	83.3±7.9	76.5±9.2	88.0±13.1
BP response to low sodium (mmHg)					
Systolic	-8.65±9.52	-3.90±5.41	-6.15±7.88	-2.38±4.79	-
Diastolic	-6.00±6.71	-3.64±4.83	-3.48±6.36	-2.70±5.21	-
Mean arterial pressure	-6.88±7.07	-3.73±4.55	-4.37±6.52	-2.59±4.56	-
BP response to high sodium (mmHg)					
Systolic	7.16±7.40	5.09±6.50	5.93±7.90	1.72±4.07	-
Diastolic	3.49±7.33	2.29±5.73	1.51±4.69	0.22±4.52	-
Mean arterial pressure	4.71±6.86	3.22±5.60	2.98±5.61	0.72±3.79	-
BP response to high sodium and potassium supplement (mmHg)					
Systolic	-6.54±5.65	-5.48±5.86	-5.59±7.10	-1.02±4.15	-
Diastolic	-3.21±4.76	-2.63±4.88	-2.69±4.42	-1.02±4.25	-
Mean arterial pressure	-4.32±4.40	-3.58±4.77	-3.66±5.05	-1.02±3.35	-

Continuous variables are expressed as mean ± SD. BP, blood pressure

2.0.4, http://www.biostat. harvard.edu/fbat/default.html) to test the association of single marker with adjusted phenotypes. Three genetic models (additive, dominant, and recessive) were tested. To assess the effect of genetic variants on the trait value, a univariate FBAT test was performed for each allele. This test provides a z-statistic with its corresponding P-value. The false discovery rate (FDR) method was used to correct for multiple testing.

Result

Baseline Characteristics and BP Response to Dietary Intervention

We recruited 515 individuals from 124 families, including 26 families with 3 generations of pedigree and 98 families with 2 generations of pedigree. All the families had 2 or more children. Table 1 shows the baseline characteristics and BP responses to the low-salt, high-salt and high-salt + potassium supplementation interventions among family members. The probands had higher mean baseline SBP and DBP than their siblings, spouses, and offspring. In general, BP levels decreased from baseline to low-salt intervention, increased from low-salt to high-salt intervention, and decreased again from the high-salt intervention to the high-salt plus potassium supplementation intervention. The BP responses during the three dietary interventions were greater in probands compared with their siblings, spouses, and offspring.

Effects of Salt Intake and Potassium Supplementation on 24-hour Urinary Sodium and Potassium Excretions

The 24-h sodium and potassium excretions in the urine were calculated at the end of each intervention period to ensure the subjects' compliance with the study protocol. At baseline, the high sodium and low potassium excretions indicated that the dietary pattern in

KARGER
Downloaded by:
Xi'an Jiaotong University
198.143.53.33 - 4/20/2016 6:34:59 AM

Table 2. Influence of dietary intervention on urinary sodium and potassium excretions

	Probands	Siblings	Spouses	Offspring
Baseline				
24 h Urinary sodium, mmol	225.1±11.6	213.8±16.4	218.3±20.8	205.4±23.4
24 h Urinary potassium, mmol	36.8±10.8	38.1±9.13	39.4±12.3	35.8±15.7
Low-sodium intervention				
24 h Urinary sodium, mmol	54.9±11.3	53.6±9.8	52.8±13.5	58.2±15.1
24 h Urinary potassium, mmol	35.4±8.9	39.8±7.6	34.2±6.7	40.5±9.8
High-sodium intervention				
24 h Urinary sodium, mmol	317.0±21.8	304.4±28.6	318.6±20.5	298.4±25.1
24 h Urinary potassium, mmol	43.4±13.9	39.6±9.1	40.2±8.3	41.1±14.5
High sodium and potassium supplement intervention				
24 h Urinary sodium, mmol	319.8±24.2	331.2±25.9	324.7±28.4	327.3±21.8
24 h Urinary potassium, mmol	86.8±9.6	79.3±11.5	94.6±12.9	87.5±10.7

Continuous variables are expressed as mean ± SD

Table 3. Characteristics of the *RNLS* SNPs analyzed in this study

SNP	Position	Region	Alleles [a]	MAF	*P*-value[b†]
rs919115	90097239	Intron 5	A/G	0.42	0.876
rs1582224	90142490	Intron 4	G/A	0.39	0.469
rs792205	90166989	Intron 4	T/C	0.49	0.833
rs7913372	90184916	Intron 4	A/G	0.42	0.781
rs12357948	90202273	Intron 4	C/T	0.12	0.014
rs12356177	90243176	Intron 4	T/C	0.07	0.608
rs12357364	90290625	Intron 4	G/A	0.14	0.964

SNP, single nucleotide polymorphism; MAF, minor allele frequency. †parents only (parental generation); [b]*P* values of Hardy-Weinberg equilibrium test; [a] Alleles are presented as major : minor allele

Northern China is characterized by high salt intake and insufficient intake of potassium, consistent with our previous survey [18]. As shown in Table 2, the urinary sodium excretion significantly decreased from baseline to the low-salt diet, but increased from the low-salt to high-salt diet (all $P < 0.05$). Potassium supplementation resulted in an increase in urinary potassium excretion and a slight increase in urinary sodium excretion. These results confirmed the subjects' compliance with the dietary intervention protocol.

Allele Frequencies and Hardy-Weinberg Equilibrium Test

Table 3 lists the genomic location, minor allele frequency, and Hardy-Weinberg tests for the 7 *RNLS* SNPs analyzed. No SNPs deviated statistically significantly from Hardy-Weinberg equilibrium, except for SNP rs12357948, which was excluded from the further analysis.

RNLS and BP Response to Dietary Intervention

As shown in Table 4, FBAT identified significant associations for rs919115 and rs792205 with DBP and MAP responses high-salt intervention. SNP rs12356177 was significantly associated with SBP and DBP responses to low-salt intervention. Furthermore, rs12356177 were also found to be associated with SBP, DBP and MAP responses during the high-salt intervention (all $P < 0.05$). However, we did not observe any association between the analyzed SNPs and BP response to the potassium supplementation.

To further confirm the association between these SNPs and BP response to salt interventions, we also performed haplotype-based analysis based on its LD block structure, as defined by Haploview software. Regretfully, no haplotype was constructed in these SNPs (Figure 1).

KARGER
Downloaded by:
Xi'an Jiaotong University
198.143.53.33 - 4/20/2016 6:34:59 AM

Kidney Blood Press Res 2014;39:497-506
DOI: 10.1159/00036846
Published online: November 26, 2014
© 2014 S. Karger AG, Basel
www.karger.com/kbr

Table 4. Summary of individual SNPs significantly associated with BP response to low-sodium, high-sodium interventions and high-sodium-and potassium supplement interventions

		SBP response		DBP response		MAP response	
SNP	Allele	z	*P*	z	*P*	z	*P*
Low-sodium intervention							
rs919115	G	-0.848	0.396	-0.741	0.459	-0.858	0.391
rs1582224	A	-0.928	0.354	-0.943	0.346	-1.023	0.306
rs792205	C	0.656	0.512	0.361	0.718	0.510	0.610
rs7913372	G	0.235	0.814	-1.178	0.239	-0.715	0.474
rs12356177	C	**-2.009**	**0.045**[b]	**2.045**	**0.041**[a]	1.680	0.093
rs12357364	A	0.470	0.638	1.946	0.052	1.498	0.134
High-sodium intervention							
rs919115	G	-0.856	0.392	**-2.418**	**0.016**[b]	**-2.327**	**0.020**[b]
rs1582224	A	-0.572	0.567	0.276	0.783	-0.017	0.987
rs792205	C	0.574	0.566	**2.101**	**0.036**[a]	**1.984**	**0.047**[a]
rs7913372	G	-0.087	0.931	-1.283	0.200	-0.963	0.336
rs12356177	C	**-2.502**	**0.012**[b]	**-2.484**	**0.013**[b]	**-2.533**	**0.011**[b]
rs12357364	A	-0.303	0.762	-0.165	0.869	-0.226	0.821
High-sodium-and potassium supplement intervention							
rs919115	G	-0.395	0.693	-0.353	0.724	-0.410	0.682
rs1582224	A	-0.245	0.806	0.672	0.501	0.583	0.560
rs792205	C	-0.155	0.877	-0.188	0.851	-0.198	0.843
rs7913372	G	-0.162	0.871	-0.302	0.762	-0.288	0.773
rs12356177	C	-0.089	0.929	-0.072	0.943	-0.087	0.493
rs12357364	A	-0.122	0.903	-0.200	0.841	-0.096	0.923

For associations that were not significant under any model, Z and P values for an additive model are listed. All genetic models are based on the minor allele of each SNP. BP, blood pressure; SBP, systolic blood pressure; DBP, diastolic blood pressure. MAP, mean arterial pressure; SNP, single nucleotide polymorphism; *P* values are corrected for multiple testing (FDR < 0.05). z indicates test statistic for FBAT; [a] dominant model; [b] recessive model; [c] additive model

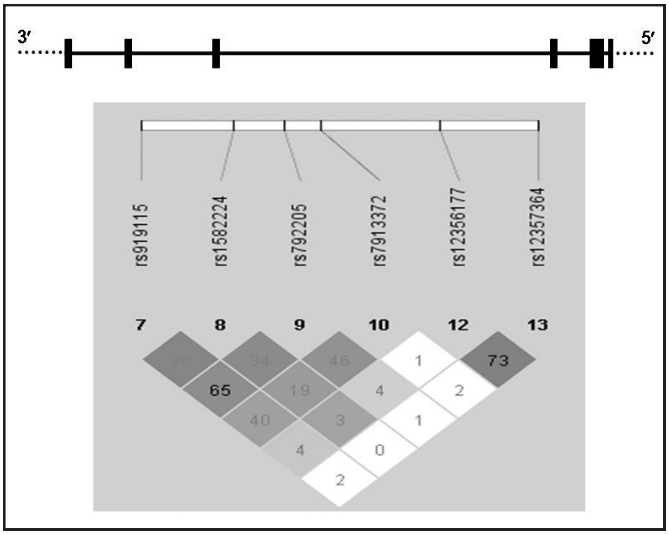

Fig. 1. The gene structures of *RNLS* and the position of single-nucleotide polymorphisms. The black boxes in each gene represent exons. Solid lines show introns and dashed lines show gene flanking regions. Pairwise linkage disequilibrium (LD) coefficients D′×100 are shown in each cell (D′ values of 1.0 are not shown). The r^2 color scheme of Haploview was applied (r^2 = 0 shown in white, 0 < r^2 <1 shown in shades of gray, and r^2 = 1 shown in black).

Association between RNLS and BP levels at baseline

Table 5 displays the association between each SNP and baseline levels of SBP, DBP and MAP adjusted for age, gender, body mass index. Single-locus analyses conducted with

KARGER
Downloaded by:
Xi'an Jiaotong University
198.143.53.33 - 4/20/2016 6:34:59 AM

the use of FBAT revealed no significant evidence of an association of BP level with the six common variations in *RNLS* gene (all $P > 0.05$). To further increase the statistical power of the study, we also applied the 3 genetic models and false discovery rate (FDR) method, but we still found no significant differences.

Table 5. Association between *RNLS* SNPs and BP levels at baseline

SNP	Allele	Additive *P* value	Dominant *P* value	Recessive *P* value
Systolic blood pressure				
rs919115	G	0.826	0.618	0.852
rs1582224	A	0.419	0.619	0.415
rs792205	C	0.718	0.978	0.584
rs7913372	G	0.290	0.358	0.496
rs12356177	C	0.817	0.763	0.548
rs12357364	A	0.758	0.722	0.746
Diastolic blood pressure				
rs919115	G	0.553	0.483	0.839
rs1582224	A	0.187	0.628	0.078
rs792205	C	0.523	0.815	0.461
rs7913372	G	0.661	0.469	0.928
rs12356177	C	0.362	0.488	0.161
rs12357364	A	0.129	0.159	0.438
Mean arterial pressure				
rs919115	G	0.639	0.514	0.959
rs1582224	A	0.235	0.602	0.139
rs792205	C	0.580	0.891	0.489
rs7913372	G	0.460	0.392	0.802
rs12356177	C	0.503	0.574	0.409
rs12357364	A	0.287	0.313	0.644

All genetic models are based on the minor allele of each SNP. BP, blood pressure; SNP, single nucleotide polymorphism

Discussion

This family-based association study identified multiple SNPs in *RNLS* gene, such as rs919115, rs792205, rs12356177, were associated with BP responses to changes in dietary salt intake. These results suggested that the *RNLS* gene might be mechanistically involved in BP salt-sensitivity and their genetic variants might contribute to the variation of this complex phenotype.

RNLS resides on chromosome10 at q23.33 has nine exons spanning 311000 base pairs and encodes a 342 amino acids protein with a calculated molecular mass of approximately 38 kDa [11]. Recently, renalase has been implicated in BP regulation and reported to be associated with hypertension. For example, Recombinant hRenalase1 administered subcutaneously has a potent hypotensive effect in 5/6 nephrectomy and stroke-prone spontaneously hypertensive rats [19]. A single subcutaneous injection of 1.3mg/kg hRenalse1 is as effective in decreasing BP as is 5mg/kg enalapril administered orally over 24 hours [19]. Schlaich et al. [20] showed that in patients with resistant hypertension, arterial renalase was significantly higher in 4 normotensive control participants relative to 22 hypertensive patients ($P < 0.05$). Additionally, few studies have investigated the relationship between polymorphisms in the *RNLS* gene and hypertension; however, findings are conflicting. SNP rs2576178 and rs2296545 within the *RNLS* gene were associated with essential hypertension among 2586 Chinese individuals [13]. After adjustments for age, gender, BMI, glucose, lipids, renal function, smoking and alcohol use, these two SNPs were associated with increased odds of hypertension (odds ratio, OR=1.58 for rs2576178 GG and 1.61 for rs2296545 CC, both $P = 0.0002$) [13]. Stec et al. [15] reported that among hypertensive hemodialyzed patients, the G allele frequencies of both rs2576178 and rs10887800 SNP in the *RNLS* gene were associated with hypertension (OR=1.55 for rs2576178, OR=1.76 for rs10887800). In the present study, no genotyped SNPs of *RNLS* were significantly associated with BP levels at baseline after correcting for age, gender and body mass index. Our results are similar to those of two separate reports of negative findings [21, 22]. These differences may be explained by different design, selection criteria, clinical characteristics and genetic background among studies.

KARGER
Downloaded by:
Xi'an Jiaotong University
198.143.53.33 - 4/20/2016 6:34:59 AM

It has been established that BP levels are heritable with the proportion of variance explained by genetic factors ranging from 20 to 50% [23]. Previous studies have revealed that an individual's genetic profile may contribute to their BP responses to dietary salt intake. In addition, Ghosh et al. [24] discovered that renalase expression levels are regulated by salt intake. Blood and kidney tissue renalase levels were significantly lower in Dahl salt-sensitive (DSS) rats maintained on an 8% salt diet for 3 weeks and became virtually undetectable after 4 weeks of a high-salt diet [24]. This observation had been confirmed by Desir G [25]. Similarly, our preliminary study found that after 4 weeks of salt intake, the expression of renalase in kidneys of DSS rats significantly decreased, and this trend was more obvious than their counterparts (SS-13^{BN} rats) (unpublished results). These studies suggested that renalase might be involved in the pathogenesis of BP salt sensitivity. In the current study, we identified three SNPs in the *RNLS* gene, rs919115, rs792205, and rs12356177 that were significantly associated with BP responses after adjustment for multiple testing. These three SNPs are located at the intronic region and have no inferred functional implication, based on the analysis by Fast SNP. However, evidence has suggested that intronic polymorphisms may be etiologically involved in the development of complex disorders [26]. Additional studies designed to investigate the positive associations reported here are warranted, and functional studies are needed to elucidate the potential mechanism underlying the association of the *RNLS* gene polymorphisms with BP responses to salt intake.

Observational epidemiologic studies have reported an inverse association between dietary potassium intake and BP [8, 18, 27]. Clinical trials have documented that potassium supplementation reduced BP in both hypertensive and normotensive subjects [8, 18]. The BP-lowering effect of potassium supplementation might vary among individuals. The GenSalt study showed that BP response to potassium supplementation varied even in a homogenous Han Chinese population [28]. These findings suggest that "potassium sensitivity" of BP might exist in humans. Genetic mutations were previously found to be associated with BP response to potassium supplementation in few studies. Zhao et al. [29] discovered an association between genetic variants in the *APLN* and *ACE2* genes and BP responses to potassium supplementation in a Han Chinese population. This group also reported that polymorphisms of key genes in vascular endothelial pathways were related to BP response to potassium including endothelin 1 (*EDN1*), nitric oxide synthase 3 (*NOS3*), and E selectin (*SELE*) in rural Chinese [30]. However, the association between *RNLS* and BP responses to potassium supplementation has never been studied. Our study is the first investigation to examine the association between common variants of *RNLS* gene and BP responses to dietary potassium intervention, however, no significant relationship was observed. This might be due to the genetic heterogeneity across populations, small effect size or low power. Replication of these results in the current analysis is needed in the future.

The present study has several important strengths and limitations. The subjects were recruited from several neighboring rural communities that were similar with respect to lifestyle and environmental risk factors, including diet and physical activity. Furthermore, because this study was based on the family pedigree, the within-family association test can eliminate the effect of an admixed and stratified population using FBAT. Thus, confounding of genetic associations due to these factors should have been minimal. In addition, the compliance of subjects to the dietary intervention was excellent, as evidenced by 24-hour urinary sodium and potassium excretions assessments. Furthermore, BP response to dietary sodium and potassium intervention was analyzed as a continuous variable, rather than dichotomous trait (such as sodium-or potassium-sensitive and sodium-or potassium-resistant groups), to improve the sensitivity of the detection of the small effect of the gene polymorphisms. In contrast, the study population was relatively small and restricted to Northern Chinese individuals. Therefore, our results will require replication in other cohorts to determine generalizability to other ethnicities and to populations with different backgrounds. Moreover, further studies to identify the causal polymorphism loci along with their functions are also critically important.

KARGER
Downloaded by:
Xi'an Jiaotong University
198.143.53.33 - 4/20/2016 6:34:59 AM

Conclusion

We reported here for the first time significant associations between genetic variants of the *RNLS* gene and BP responses to dietary salt intervention in a Northern Chinese population. These findings may contribute to a better understanding of the genetic mechanisms underlying BP regulation and may have potential clinical and public health implications.

Disclosure Statement

The authors declare that there is no conflict of interest.

Acknowledgements

We are indebted to the participants in the study and their families for their outstanding commitment and cooperation. This study was supported by grant 2012CB517804 from the National Program on Key Basic Research Project of China (973 Program) and grants 81200512 (Dr. Liu), 81070218 and 81370357 (Dr. Mu) from the Natural Science Foundation of China.

References

1 Kearney PM, Whelton M, Reynolds K, Muntner P, Whelton PK, He J: Global burden of hypertension: analysis of worldwide data. Lancet 2005;365:217-223.
2 Danaei G, Finucane MM, Lin JK, Singh GM, Paciorek CJ, Cowan MJ, Farzadfar F, Stevens GA, Lim SS, Riley LM, Ezzati M: National, regional, and global trends in systolic blood pressure since 1980: systematic analysis of health examination surveys and epidemiological studies with 786 country-years and 5.4 million participants. Lancet 2011;377:568-577.
3 Kato N, Takeuchi F, Tabara Y, Kelly TN, Go MJ, Sim X, Tay WT, Chen CH, Zhang Y, Yamamoto K, Katsuya T, Yokota M, Kim YJ, Ong RT, Nabika T, Gu D, Chang LC, Kokubo Y, Huang W, Ohnaka K, Yamori Y, Nakashima E, Jaquish CE, Lee JY, Seielstad M, Isono M, Hixson JE, Chen YT, Miki T, Zhou X, Sugiyama T, Jeon JP, Liu JJ, Takayanagi R, Kim SS, Aung T, Sung YJ, Zhang X, Wong TY, Han BG, Kobayashi S, Ogihara T, Zhu D, Iwai N, Wu JY, Teo YY, Tai ES, Cho YS, He J: Meta-analysis of genome-wide association studies identifies common variants associated with blood pressure variation in east Asians. Nat Genet 2011;43:531-538.
4 He J, Whelton PK: Salt intake, hypertension and risk of cardiovascular disease: an important public health challenge. Int J Epidemiol 2002;31:327-331.
5 Appel LJ, Brands MW, Daniels SR, Karanja N, Elmer PJ, Sacks FM: Dietary approaches to prevent and treat hypertension: a scientific statement from the American Heart Association. Hypertension 2006;47:296-308.
6 Denton D, Weisinger R, Mundy NI, Wickings EJ, Dixson A, Moisson P, Pingard AM, Shade R, Carey D, Ardaillou R: The effect of increased salt intake on blood pressure of chimpanzees. Nat Med 1995;1:1009-1016.
7 Elliott P, Stamler J, Nichols R, Dyer AR, Stamler R, Kesteloot H, Marmot M: Intersalt revisited: further analyses of 24 hour sodium excretion and blood pressure within and across populations. Intersalt Cooperative Research Group. BMJ 1996;312:1249-1253.
8 Whelton PK, He J, Cutler JA, Brancati FL, Appel LJ, Follmann D, Klag MJ: Effects of oral potassium on blood pressure. Meta-analysis of randomized controlled clinical trials. JAMA 1997;277:1624-1632.
9 He J, Gu D, Chen J, Jaquish CE, Rao DC, Hixson JE, Chen JC, Duan X, Huang JF, Chen CS, Kelly TN, Bazzano LA, Whelton PK: Gender difference in blood pressure responses to dietary sodium intervention in the GenSalt study. J Hypertens 2009;27:48-54.
10 Xu J, G Li, Wang P, Velazquez H, Yao X, Li Y, Wu Y, Peixoto A, Crowley S, Desir GV: Renalase is a novel, soluble monoamine oxidase that regulates cardiac function and blood pressure. J Clin Invest 2005;115:1275-1280.

KARGER
Downloaded by:
Xi'an Jiaotong University
198.143.53.33 - 4/20/2016 6:34:59 AM

11 Desir GV: Regulation of blood pressure and cardiovascular function by renalase. Kidney Int 2009;76:366-370.
12 Gu R, Lu W, Xie J, Bai J, Xu B: Renalase deficiency in heart failure model of rats--a potential mechanism underlying circulating norepinephrine accumulation. Plos One 2011;6:e14633.
13 Zhao Q, Fan Z, He J, Chen S, Li H, Zhang P, Wang L, Hu D, Huang J, Qiang B, Gu D: Renalase gene is a novel susceptibility gene for essential hypertension: a two-stage association study in northern Han Chinese population. J Mol Med 2007;85:877-885.
14 Buraczynska M, Zukowski P, Buraczynska K, Mozul S, Ksiazek A: Renalase gene polymorphisms in patients with type 2 diabetes, hypertension and stroke. Neuromolecular Med 2011;13:321-327.
15 Stec A, Semczuk A, Furmaga J, Ksiazek A, Buraczynska M: Polymorphism of the renalase gene in end-stage renal disease patients affected by hypertension. Nephrol Dial Transplant 2012;27:4162-4166.
16 Liu F, Zheng S, Mu J, Chu C, Wang L, Wang Y, Xiao H, Wang D, Cao Y, Ren K, Liu E, Yuan Z: Common variation in with no-lysine kinase 1 (WNK1) and blood pressure responses to dietary sodium or potassium interventions- family-based association study. Circ J 2013;77:169-174.
17 Gu D, Zhao Q, Chen J, Chen JC, Huang J, Bazzano LA, Lu F, Mu J, Li J, Cao J, Mills K, Chen CS, Rice T, Hamm LL, He J: Reproducibility of blood pressure responses to dietary sodium and potassium interventions: the GenSalt study. Hypertension 2013;62:499-505.
18 Mu J, Liu Z, Liu F, Xu X, Liang Y, Zhu D: Family-based randomized trial to detect effects on blood pressure of a salt substitute containing potassium and calcium in hypertensive adolescents. Am J Hypertens 2009;22:943-947.
19 Baraka A, El Ghotny S: Cardioprotective effect of renalase in 5/6 nephrectomized rats. J Cardiovasc Pharmacol Ther 2012;17:412-416.
20 Schlaich M, Socratous F, Eikelis N, Chopra R, Lambert G, Hennebry S: Renalase plasma levels are associated with systolic blood pressure in patients with resistant hypertension. J Hypertens 2010;28:e437.
21 Fava C, Montagnana M, Danese E, Sjögren M, Almgren P, Engström G, Hedblad B, Guidi GC, Minuz P, Melander O: The Renalase Asp37Glu polymorphism is not associated with hypertension and cardiovascular events in an urban-based prospective cohort: the Malmo Diet and cancer study. BMC Med Genet 2012;13:57.
22 Abdallah E, Sabry D: Renalase gene polymorphisms in end-stage renal disease patients: An Egyptian study. J Am Sci 2013;9:346-349.
23 Saavedra JM: Studies on genes and hypertension: a daunting task. J Hypertens 2005;23:929-932.
24 Ghosh SS, Gehr TWB, Sica DA, Masilamani S, Fakhry I, Wang R, McGuire E, Ghosh S: Renalase regulates blood pressure in salt sensitive Dahl rats. J Am Soc Nephrol 2006;17:208A.
25 Desir G: Novel insights into the physiology of renalase and its role in hypertension and heart disease. Pediatr Nephrol 2012;27:719-725.
26 Tokuhiro S, Yamada R, Chang X, Suzuki A, Kochi Y, Sawada T, Suzuki M, Nagasaki M, Ohtsuki M, Ono M, Furukawa H, Nagashima M, Yoshino S, Mabuchi A, Sekine A, Saito S, Takahashi A, Tsunoda T, Nakamura Y, Yamamoto K: An intronic SNP in a RUNX1 binding site of SLC22A4, encoding an organic cation transporter, is associated with rheumatoid arthritis. Nat Genet 2003;35:341-348.
27 Geleijnse JM, Kok FJ, Grobbee DE: Blood pressure response to changes in sodium and potassium intake: a metaregression analysis of randomised trials. J Hum Hypertens 2003;17:471-480.
28 Gu D, Rice T, Wang S, Yang W, Gu C, Chen CS, Hixson JE, Jaquish CE, Yao ZJ, Liu DP, Rao DC, He J: Heritability of blood pressure responses to dietary sodium and potassium intake in a Chinese population. Hypertension 2007;50:116-122.
29 Zhao Q, Gu D, Kelly TN, Hixson JE, Rao DC, Jaquish CE, Chen J, Huang J, Chen CS, Gu CC, Whelton PK, He J: Association of Genetic Variants in the Apelin–APJ System and ACE2 With Blood Pressure Responses to Potassium Supplementation: The GenSalt Study. Am J Hypertens 2010;23:606-613.
30 Montasser ME, Shimmin LC, Gu D, Chen J, Gu C, Kelly TN, Jaquish CE, Rice T, Rao DC, Cao J, Chen J, Liu DP, Whelton P, He J, Hixson JE: Blood pressure response to potassium supplementation is associated with genetic variation in endothelin 1 (EDN1) and interactions with E selectin (SELE) in rural Chinese. J Hypertens 2010;28:748.

KARGER
Downloaded by:
Xi'an Jiaotong University
198.143.53.33 - 4/20/2016 6:34:59 AM
