# Supplementary material for: Association of Renalase SNPs rs2296545 and rs2576178 with the Risk of Hypertension: A Meta-Analysis
Source: PLoS One. 2016 Jul 19;11(7):e0158880. doi: 10.1371/journal.pone.0158880 (PMC4951046; doi:10.1371/journal.pone.0158880)
Supplement: S3 File — (ZIP) [file pone.0158880.s003.zip › 11 excluded records/Anna Stec et al. 2012.pdf]

Nephrol Dial Transplant (2012) 27: 4162–4166
doi: 10.1093/ndt/gfr293
Advance Access publication 26 May 2011

Polymorphism of the renalase gene in end-stage renal disease patients affected by hypertension

Anna Stec[1], Andrzej Semczuk[2], Jacek Furmaga[3], Andrzej Ksiazek[1] and Monika Buraczynska[4]

[1]Department of Nephrology, University Medical School, Lublin, Poland, [2]Second Department of Gynecology, University Medical School, Lublin, Poland, [3]Department of General and Transplant Surgery, University Medical School, Lublin, Poland and [4]Department of Nephrology, Laboratory for Molecular Diagnostics of Multifactorial Diseases, University Medical School, Lublin, Poland

Correspondence and offprint requests to: Anna Stec; E-mail: annastec@poczta.onet.pl

Downloaded from http://ndt.oxfordjournals.org/ at Xi'an Jiaotong University on April 19, 2016

Abstract

Background. Renalase is a novel flavin adenine dinucleotide-dependent amine oxidase that is secreted by the kidney. It circulates in the blood and modulates the cardiac function and systemic blood pressure. Insufficiency of renalase in patients with chronic kidney disease may explain the frequent occurrence of hypertension among patients with end-stage renal disease (ESRD) and an increased risk of cardiovascular events in this group. The aim of the study was to assess the relationship of two renalase gene polymorphisms with hypertension in dialysed patients.

Methods. Rs2576178 polymorphism was genotyped in 369 patients, rs10887800 polymorphism was genotyped in 421 dialysed patients, using polymerase chain reaction (PCR) and subsequent cleavage with Msp I and Pst I restriction endonucleases.

Results. Genotype distribution and allele frequencies of rs2576178 polymorphism were compared in the following subgroups of patients: dialysed patients with hypertension: ESRD HY + ($n = 200$) and dialysed patients without hypertension: ESRD HY − ($n = 169$). There was a significant difference in the frequency of the G allele carriers. G allele carriers were associated with a 1.55 times higher risk of hypertension [odds ratio (OR) = 1.55; 95% confidence interval (CI): 1.023–2.357, $P = 0.039$]. Distribution of genotypes and frequencies of alleles of rs10887800 polymorphism were compared in the following subgroups of patients: ESRD HY + ($n = 278$) and ESRD HY − ($n = 143$). The G allele carriers were recognized with a significantly higher frequency in ESRD HY + patients (0.46 in ESRD HY + versus 0.37 in ESRD HY −) [OR = 1.76; 95% CI: (1.159–2.667, $P = 0.008$)].

Conclusions. Our results are the first to suggest an association between renalase gene polymorphisms analysed and hypertension in dialysed patients. It may be an important step towards gaining a deeper insight into cardiovascular pathophysiology. Furthermore, it might provide an optimal treatment and better prognosis for patients with chronic kidney disease.

Keywords: end-stage renal disease; hypertension; renalase; single-nucleotide; polymorphism

Introduction

In 2005, Xu *et al.* [1] described renalase as a novel protein produced by the kidneys, secreted into the blood stream and participating in the metabolism of circulating catecholamines. The human renalase gene is located on chromosome 10 (q23.33), has 10 exons, and encodes a protein of 311 kbp. Renalase belongs to a group of amine oxidases containing flavin adenine dinucleotide (FAD) as a cofactor [2]. This group also includes amine oxidase [monoaminooxidase (MAO)] and polyamide oxidase [3–5].

In contrast to well-known amine oxidases containing FAD, renalase is synthesized mainly by the kidneys and is excreted directly into the blood, where it participates in the metabolism of circulating catecholamines [6]. Renalase activity is not inhibited by known MAO inhibitors such as pargyline and clorgyline [7, 8]. To emphasize these differences, renalase is called monoaminooxidase C (MAO-C) [9].

At least four isoforms of renalase molecules have been shown. Two of them possess an unchanged amino acid domain (h-renalase 1), whereas two of them have shortened domains (h-renalase 2). H-renalase 1 contains 342 amino acids and has a molecular weight of 38 kDa. H-renalase 2 has 315 amino acids and differs from the previous in the C-terminus of the polypeptide chain. Both isoforms show signs of overlap: the signal peptide (amino acids 1–17) and FAD-binding domain (amino acids 4–45) on the N-terminus [9].

Li *et al.* [10] described a multiple step mechanism regulating renalase synthesis and secretion. The increased

© The Author 2011. Published by Oxford University Press on behalf of ERA-EDTA. All rights reserved.
For Permissions, please e-mail: journals.permissions@oup.com

concentration of catecholamines, with a subsequent increase in systolic blood pressure (SBP), seemed to be the key factor stimulating the activation of MAO-C. Interestingly, diastolic and mean blood pressure did not correlate with renalase activity.

Renalase plays a direct and significant role in the regulation of blood pressure. Insufficiency of renalase predisposes to higher blood pressure values [11, 12].

Patients with end-stage renal disease (ESRD) have a significant renalase deficiency. These observations were confirmed by animal experimental models of uraemia in rats after 5/6 nephrectomies [13]. Renalase deficiency observed in patients with renal failure leads to impaired degradation of catecholamines and, as a consequence, to elevated serum concentration causing excessive tension of the sympathetic nervous system. High activity of the sympathetic nervous system is related to a high risk of cardiovascular diseases [14–16].

Therefore, renalase deficiency could be an unknown pathophysiological mechanism and might at least partially explain the high rates of hypertension in ESRD patients.

The aim of the current study was to analyse the potential association of two polymorphisms of the renalase gene with hypertension in patients with ESRD.

Materials and methods

Patients

Rs2576178 polymorphism was genotyped in 369 dialysed patients including 200 hypertensive cases and 169 controls (patients without hypertension). R*s10887800* polymorphism was genotyped in 421 dialysed patients including 278 hypertensive cases and 143 controls (patients without hypertension). The patients, Caucasians of Polish origin, had undergone renal replacement therapy in a form of haemodialysis or peritoneal dialysis. ESRD resulted from chronic glomeluronephritis (29.7%), diabetic nephropathy (20%), interstitial nephritis (12.5%), polycystic kidney disease (8%), hypertensive nephropathy (7.1%), obstructive nephropathy (3.5%) and other causes. Demographic and clinical characteristics of the groups studied are shown in Table 1.

Patients were enrolled into the study consistently from dialysis centres located at Lublin region. The allocating to particular study group (ESRD HY + or ESRD HY −) depended on the presence or absence of hypertension diagnosis. Hypertension was defined as a SBP of ≥140 mmHg, diastolic blood pressure (DBP) of ≥90 mmHg or taking antihypertensive medication. All blood pressure measurements were determined by office blood pressure methods with a mercury manometer. Patient was at a supine position for at least 5 min before measurement. The average values of predialysis, systolic and DBP reported in the first 4 weeks of the study were collected and used for the analysis. Mean arterial pressure was calculated from the following standard equation: 1/3 of the SBP + 2/3 of the DBP. Blood pressure data for examined groups of dialysed patients are shown in Table 2. Informed consent for genetic studies was obtained from all subjects. The protocol of the study was approved by the Ethics Committee of the University Medical School in Lublin, Poland.

Isolation of human genomic DNA

Human genomic DNA was isolated from peripheral blood leukocytes according to the technique of Madisen *et al.* [17] with minor modifications.

Determination of genotypes of the studied polymorphisms by polymerase chain reaction

DNA was amplified by polymerase chain reaction (PCR) with primers specific for renalase gene polymorphisms [18]. The composition of a typical 30 µL PCR reaction included: 300 ng genomic DNA, 10 mM TRIS–HCl buffer (pH 8.3), 50 mM KCl, 1.5 mM $MgCl_2$, 200 µM each of ditag primer, 2 U *Taq* of DNA polymerase. All reagents were obtained from MBI Fermentas Company (St Leon-Rot, Germany). Amplification of DNA was carried out in a PTC200 thermocycler (MJ Research, Inc. Waltham, MA).

Table 1. Demographic and clinical characteristics of analysed group of patients[a]

Characteristic	Dialysed patients
Age (in years)	54.60 ± 15.71
BMI (kg/m^2)	25.49 ± 4.93
Total cholesterol (mg/dL)	187.79 ± 51.97
HDL-cholesterol (mg/dL)	46.88 ± 41.09
Trigliceryde (mg/dL)	170.11 ± 97.85
LDL-cholesterol (mg/dL)	110.44 ± 62.82
Serum creatinine (mg/dL)	7.18 ± 31
Dialysis time (in years)	2.78 ± 3.62
Time to ESRD (in years)	6.46 ± 8.57

[a]BMI, body mass index; HDL, high-density lipoprotein; LDL, low-density lipoprotein.

Table 2. Blood pressure data for examined groups of dialysed patients[a]

Blood pressure (mmHg)	ESRD HY +[b] (mean ± SD)	ESRD HY −[c] (mean ± SD)
SBP	148.17 ± 5.78	118.31 ± 6.23
DBP	89.54 ± 4.66	68.48 ± 5.34
MAP	109.02 ± 7.12	85.12 ± 4.42

[a]There was no statistical difference in the number of years on dialysis between ESRD HY + and ESRD HY − groups.
[b]ESRD HY + , dialysed patients with hypertension.
[c]ESRD HY − , dialysed patients without hypertension; MAP, mean arterial pressure.

PCR reaction conditions were as follows: initial DNA denaturation step for 6 min at 94–96°C, followed by 30–35 cycles of amplification, DNA denaturation for 1 min at 96°C, annealing of primers for 1 min in the temperature dependent on used primers, DNA chain elongation (extension) for 1–2 min at 72°C. The final step of DNA chain elongation lasted 7–10 min at 72°C. In the case of restriction fragment length polymorphism, PCR products were digested with the appropriate restrictive endonuclease at a temperature of 37°C for 6–10 h. The reaction products were separated by electrophoresis in 1.5–2.5% agarose gel.

rs2576178 polymorphism of the renalase

This polymorphism consisted of a substitution of a single-nucleotide polymorphism (SNP) G → A in the 5'-flanking region of the gene. Amplification was performed using primers: sense: 5'-AGCAGAGAAG-CAGCTTAACCT-3' antisense: 5'-TTATCTGCAAGTCAGCGTAAC-3'. The binding temperature of the primers in the PCR reaction was 60°C. PCR product length was 525 bp. After digestion with restrictive endonuclease Msp I (SMEs), 423 + 102 bp fragments for GG genotype and 525 bp for AA genotype were observed.

rs10887800 polymorphism of the renalase

This polymorphism consisted of a substitution of a SNP A → G in intron 6 of the gene. Amplification using primers: sense: 5'-CAGGAAA-GAAAGAGTTGACAT-3' antisense: 5'-AAGTTGTTCCAGCTACTGT-3'. The binding temperature of the primers in PCR reactions was 60°C. PCR product length was 554 bp. After digestion with restrictive endonuclease Pst I, fragments 554 bp for AA genotype and 415 + 139 bp for the GG genotype were observed.

Statistical analysis

Hardy–Weinberg equilibrium for alleles at individual loci was tested using the chi-square test. Data was presented as means ± standard deviations. Genotype distribution and allele frequencies were assessed by a

Downloaded from http://ndt.oxfordjournals.org/ at Xi'an Jiaotong University on April 19, 2016

chi-square test of independence with 2 × 2 contingency tables and z-statistics.

Variation analysis with the previous test for normal distribution (Shapiro–Wilk test) and homogeneity of variance (Fischer's exact test) were used to analyse the differences for the measurable parameters in genotype subgroups. Values of $P < 0.05$ were considered statistically significant. We estimated the odds ratio (OR) with 95% confidence intervals (CIs) for the effects of high-risk alleles. The statistical analysis was performed using STATISTICA v. 7.1 (StatSoft) packages.

Results

rs2576178 polymorphism of renalase

Table 3 shows the distribution of genotypes and alleles frequencies of rs2576178 polymorphism in the two subgroups of dialysed patients, with and without hypertension.

Distribution of phenotypes and frequencies of alleles of rs2576178 *renalase* polymorphism were compared in the following subgroups of patients: ESRD HY + ($n = 200$) and ESRD HY − ($n = 169$). The frequencies of genotypes in each group satisfied the Hardy–Weinberg equilibrium (P = 0.990 in ESRD HY + group and P = 0.257 in ESRD HY − group). In the whole study group, significant differences in genotype distributions were not observed (P = 0.100). The GG genotype was rare in both groups. It was slightly more often recognized among patients affected by hypertension (7.5% in the ESRD HY + versus 7.1% in the ESRD HY − group, P = 0.883). There was no relationship between the G allele homozygosity and an increased risk of hypertension [OR = 1.06 (95% CI: 0.482–2.333)]. The high frequency of the AA genotype in nonhypertensive patients (62.7% in ESRD HY − versus 52% in ESRD HY +, P = 0.038) accounted for its possible protective function. An analysis of the individual subgroups revealed a statistically higher incidence of the G allele in hypertensive patients (0.28 in the ESRD HY + versus 0.22 in ESRD HY −, P = 0.039). The carrier state of the G allele was associated with a 1.55 times higher risk of hypertension [OR = 1.55; (95% CI: 1.023–2.357)].

rs10887800 polymorphism of renalase

Table 4 shows the distribution of genotypes and frequencies of alleles of rs10887800 polymorphism in two subgroups of dialysed patients, with and without hypertension.

Genotype distribution and allele frequencies of rs10887800 *renalase* polymorphism were compared in the following subgroups of patients: ESRD HY + ($n = 278$) and ESRD HY − ($n = 143$). The genotype frequencies in each group satisfied the Hardy–Weinberg equilibrium ($P = 0.401$ for ESRD HY + group and P = 0.120 for ESRD HY − group). An analysis of the study group showed statistically significant differences in genotype distribution of the polymorphism analysed (P = 0.026). A higher prevalence of GG genotype among patients with hypertension (23.4% in the ESRD HY + versus 17.5% in the ESRD HY −) was demonstrated. G allele homozygosity was not associated with an increased risk of hypertension [OR = 1.44; (95% CI: 0.862–2.406), P = 0.163]. The high frequency of AA genotype in nonhypertensive patients (44.1% in the ESRD HY − versus 30.9% in the ESRD HY +, P = 0.008) highlighted its possible protective function. The incidence of the G allele in the subgroup of hypertensive patients was compared with nonhypertensive subjects. The difference was statistically significant. The carrier state of the G allele was associated with a 1.76 times higher risk of hypertension [OR = 1.76; (95% CI: 1.159–2.667), P = 0.008].

Table 3. The distribution of genotypes and alleles frequencies of rs2576178 *renalase* polymorphism in patients with ESRD

Genotypes	ESRD HY + [a]	ESRD HY − [b]	Significance: (P)[c]
GG, *n* (%)	15 (7.5)	12 (7.1)	GG/(GA + AA): 0.883
GA, *n* (%)	81 (40.5)	51 (30.2)	GA/(GG + AA): 0.039
AA, *n* (%)	104 (52)	106 (62.7)	AA/(GG + GA): 0.038
Total, *n* (%)	200 (100)	169 (100)	AA/AG/GG: 0.100
Alleles			
G	0.28	0.22	G/A: 0.039
A	0.72	0.78	

[a]ESRD HY +, dialysed patients with hypertension.
[b]ESRD HY −, dialysed patients without hypertension.
[c]Test of comparison of genotypes and alleles distributions (Fischer's exact test). For G allele carriers: OR = 1.55; (95% CI: 1.023–2.357), P = 0.039, For GG genotype carriers: OR = 1.06; (95% CI: 0.482–2.333), P = 0.883.

Table 4. The distribution of genotypes and alleles frequencies of rs10887800 *renelase* polymorphism in patients with ESRD

Genotypes	ESRD HY + [a]	ESRD HY − [b]	Significance: (P)[c]
AA, *n* (%)	86 (30.9)	63 (44.1)	AA/(AG + GG): 0.008
AG, *n* (%)	127 (45.7)	55 (38.5)	AG/(AA + GG): 0.157
GG, *n* (%)	65 (23.4)	25 (17.5)	GG/(AA + AG): 0.163
Total, *n* (%)	278 (100)	143 (100)	AA/AG/GG: 0.026
Alleles			
G	0.46	0.37	G/A: 0.008
A	0.54	0.63	

[a]ESRD HY +, dialysed patients with hypertension.
[b]ESRD HY −, dialysed patients without hypertension.
[c]Test of comparison of genotypes and alleles distributions (Fischer's exact test). For G allele carriers: OR = 1.76; (95% CI: 1.159–2.667), P = 0.008, For GG genotype carriers: OR = 1.44; (95% CI: 0.862–2.406), P = 0.163.

Discussion

Hypertension is a multifactorial disease, and its pathogenesis is not yet fully understood [19, 20]. Over the last few years, the genetic susceptibility to hypertension has been a special focus of attention, and many candidate genes have been studied so far [21–23].

There was only one study that investigated renalase gene polymorphisms. That study was conducted by Zhao *et al.* [24], who assessed the correlation of SNPs of the renalase gene with the primary hypertension occurrence. The authors marked eight SNP genotypes in a group of 2586 patients (1317 patients with spontaneous hypertension and 1269 healthy controls), investigated within the framework of the International Collaborative Study of Cardiovascular Disease in Asia. This two-stage

Downloaded from http://ndt.oxfordjournals.org/ at Xi'an Jiaotong University on April 19, 2016

association study estimated the relationship between essential hypertension and two polymorphisms: rs2576178 and rs2296545.

In our study, we analysed the relationship of renalase gene molecular variants with the occurrence of hypertension in ESRD patients, the group of patients at risk of hypertension, with a significantly high prevalence of death from cardiovascular events [25, 26]. The study investigated two polymorphisms of the renalase gene: rs2576178 and rs10887800.

The distribution analysis of the genotype and allele frequencies of rs2576178 renalase gene polymorphism showed a significantly higher incidence of the G allele in hypertensive dialysed patients. The carrier state of the G allele was associated with a 1.55 times higher risk of hypertension [OR = 1.55; (95% CI: 1.023–2.357), P = 0.039]. The results are similar to those published by Zhao *et al.* [24]. Although the G allele frequencies were different in both populations (0.28 in the group of hypertensive patients on dialysis versus 0.22 in the dialysed group without hypertension, P = 0.039 in the Polish population as compared with 0.55 in the hypertensive group versus 0.49 in the group without hypertension, P < 0.0001 in the Asian population), Zhao *et al.* [24] also observed a significantly higher incidence of allele G in hypertensive patients. Therefore, as a conclusion, the carrier state of the G allele increased the risk of hypertension in both populations.

In the study presented here, there was no correlation between hypertension and G allele homozygosity of rs2576178 polymorphism [OR = 1.06; (95% CI: 0.482–2.333), P = 0.883], as compared with the data reported by Zhao *et al.* [24]. As an explanation, differences in the high number of patient groups or interpopulational genetic differences may influence the results obtained. Both populations showed a significantly increased prevalence of the AA genotype in the subgroup of nonhypertensive patients, suggesting its role as a protective factor.

The analysis of the distribution of genotypes and alleles of the second renalase gene polymorphism rs10887800 displayed a significantly higher frequency of the G allele in hypertensive patients on dialysis as compared with nonhypertensive patients (0.46 versus 0.37, respectively, P = 0.008). The carrier state of the G allele was associated with a 1.75 times higher risk of hypertension [OR = 1.75; (95% CI: 1.159–2.667)]. A significantly higher incidence of AA genotype in the nonhypertensive patients, similar to the first polymorphism case, may suggest its protective function.

Zhao *et al.* [24] did not demonstrate the correlation of rs10887800 polymorphism with spontaneous hypertension. The distributions of genotypes and allele frequencies did not differ significantly between the groups analysed in this study. The differences may be due to the previously mentioned differences between the populations and the numerical disproportion of the groups.

Due to the precursory character of the two previous studies of *renalase* polymorphisms, the interpretation of the results needs careful verification. The fact that we achieved comparable results of the G allele of the rs2576178 renalase gene polymorphism in two distinct populations seems to confirm its significant role in the pathogenesis of hypertension. Despite the differences between the populations, the role of rs10887800, a second *renalase* polymorphism in the development of hypertension, cannot be finally excluded.

In conclusion, the study presented here is the first molecular analysis of *renalase* variants in the population of dialysed patients, demonstrating the relationship between polymorphisms studied and the development or existence of hypertension. It is an important step towards gaining a deeper insight into the hypertension pathophysiology and its complications. It allows us to improve the treatment and prognosis of patients affected by hypertension, especially those undergoing renal replacement therapy.

Conflict of interest statement. The results of this study were previously presented at the XLVII ERA-EDTA Congress in Munich, Germany (June 25–28, 2010) during the poster session.

References

1. Xu J, Guoyong L, Wang P *et al*. Renalase is a novel, soluble monoamine oxidase that regulates cardiac function and blood pressure. *J Clin Invest* 2005; 115: 1275–1280
2. Pandini V, Ciriello F, Tedeschi G *et al*. Synthesis of human renalase1 in Escherichia coli and its purification as a FAD-containing holoprotein. *Protein Expr Purif* 2010; 72: 244–253
3. Wang J, Edmondson DE. Do monomeric vs dimeric forms of MAO-A make a difference? A direct comparison of the catalytic properties of rat and human MAO-A's. *J Neural Transm* 2007; 114: 721–724
4. Binda C, Hubálek F, Li M *et al*. Crystal structure of human monoamine oxidase B, a drug target enzyme monotopically inserted into the mitochondrial outer membrane. *FEBS Lett* 2004; 30: 225–228
5. Wang J, Qi S, Cheng W *et al*. Identification, expression and tissue distribution of a renalase homologue from mouse. *Mol Biol Rep* 2008; 35: 613–620
6. Hennebry SC, Eikelis N, Socratous F *et al*. Renalase, a novel soluble FAD-dependent protein, is synthesized in the brain and peripheral nerves. *Mol Psychiatry* 2010; 15: 234–236
7. Boomsma F, Tipton KF. Renalase, a catecholamine-metabolising enzyme? *J Neural Transm* 2007; 114: 775–776
8. Luft FC. Renalase, a catecholamine-metabolizing hormone from the kidney. *Cell Metab* 2005; 1: 358–360
9. Desir GV. Renalase deficiency in chronic kidney disease, and its contribution to hypertension and cardiovascular disease. *Curr Opin Nephrol Hypertens* 2008; 17: 181–185
10. Li G, Xu J, Wang P *et al*. Catecholamines regulate the activity, secretion, and synthesis of renalase. *Circulation* 2008; 11: 1277–1282
11. Xu J, Desir GV. Renalase, a new renal hormone: its role in health and disease. *Curr Opin Nephrol Hypertens* 2007; 16: 373–378
12. Desir GV. Renalase is a novel renal hormone that regulates cardiovascular function. *J Am Soc Hypertens* 2007; 1: 99–103
13. Desir GV. Regulation of blood pressure and cardiovascular function by renalase. *Kidney Int* 2009; 76: 366–370
14. Schlaich MP, Socratous F, Hennebry S *et al*. Sympathetic activation in chronic renal failure. *J Am Soc Nephrol* 2009; 20: 933–939
15. Joles JA, Koomans HA. Causes and consequences of increased sympathetic activity in renal disease. *Hypertension* 2004; 43: 699–704
16. Farzaneh-Far R, Desir GV, Na B *et al*. A functional polymorphism in renalase (Glu37Asp) is associated with cardiac hypertrophy, dysfunction, and ischemia: data from the heart and soul study. *PLoS One* 2010; 5: e13496
17. Madisen L, Hoar DI, Holroyd CD *et al*. The banking: the effects of storage of blood and isolated DNA on the integrity of DNA. *Am J Med Genet* 1987; 27: 379–390

Downloaded from http://ndt.oxfordjournals.org/ at Xi'an Jiaotong University on April 19, 2016

18. Mullis K. The unusual origin of the polymerase chain reaction. *Sci Am* 1990; 262: 56–65
19. Oparil S, Zaman MA, Calhoun DA. Pathogenesis of hypertension. *Ann Intern Med* 2003; 139: 761–776
20. Paulis L, Unger T. Novel therapeutic targets for hypertension. *Nat Rev Cardiol* 2010; 7: 431–441
21. Jeunemaitre X, Gimenez-Roqueplo AP, Disse-Nicodeme S, Corvol P. Molecular basis of human hypertension. In: Rimoin DL, Connor JM, Pyeritz RE, Korf BR eds. *Emery and Rimon's Principles and Practice of Medical Genetics*, 5th edn. Philadelphia, PA: Churchill Livingstone, 2007: 1283–1300
22. Dongfeng G, Shaoyong S, Dongliang G *et al*. Association study with 33 single-nucleotide polymorphisms in 11 candidate genes for hypertension in Chinese. *Hypertension* 2006; 47: 1147–1154
23. Wilson FH, Disse-Nicodeme S, Choate KA *et al*. Human hypertension caused by mutations in WNK kinases. *Science* 2001; 293: 1107–1112
24. Zhao Q, Fan Z, He J *et al*. Renalase gene is a novel susceptibility gene for essential hypertension: a two-stage association study in northern Han Chinese population. *J Mol Med* 2007; 85: 877–885
25. Santos SF, Peixoto AJ. Hypertension in dialysis. *Curr Opin Nephrol Hypertens* 2005; 14: 111–118
26. Lameire N. Cardiovascular problems in ESRD patients. *Nefrologia* 2000; 20 (Suppl 3): 33–40

Received for publication: 15.9.2010; Accepted in revised form: 26.4.2011

Nephrol Dial Transplant (2012) 27: 4166–4172
doi: 10.1093/ndt/gfs337
Advance Access publication 20 August 2012

Growth arrest-specific gene 6 (Gas6) levels are elevated in patients with chronic renal failure

Iris J. Lee[1], Brendan Hilliard[2], Abhishek Swami[1], John C. Madara[5], Swati Rao[1], Tapan Patel[1], John P. Gaughan[3], Jean Lee[1], Crystal A. Gadegbeku[1], Eric T. Choi[4] and Philip L. Cohen[2]

[1]Section of Nephrology, Hypertension and Kidney Transplantation, Temple University, Philadelphia, PA, USA, [2]Section of Rheumatology, Temple University, Philadelphia, PA, USA, [3]Biostatistics Consulting Center, Temple University, Philadelphia, PA, USA, [4]Department of Vascular Surgery, Temple University, Philadelphia, PA, USA and [5]School of Medicine, Temple University, Philadelphia, PA, USA

Correspondence and offprint requests to: Iris J. Lee; E-mail: iris.lee@tuhs.temple.edu

Abstract

Background. The TAM receptors (tyro3, axl and mer) and their ligands (vitamin K-dependent proteins—Gas6 and Protein S) are crucial modulators of inflammation, which may be relevant in chronic kidney disease (CKD). Gas6 and axl have multiple roles in mediating vascular atherosclerosis and injury, thrombosis and inflammation, yet nothing is known about the Gas6–axl pathway in humans with CKD. Given the prevalence of chronic inflammation and vascular disease in this population, we measured TAM ligands in patients with various levels of renal function.

Methods. Gas6 and protein S were quantified in the plasma by ELISA in three patient groups: end-stage renal disease on chronic hemodialysis (HD), CKD and normal controls.

Results. Significantly increased levels of Gas6 and protein S were found in CKD patients compared with normal controls ($P < 0.01$ and <0.001, respectively). In HD patients, Gas6 levels were elevated compared with controls ($P < 0.001$) and positively associated with low albumin ($r = 0.33$; $P = 0.01$), dialysis vintage ($r = 0.36$; $P = 0.008$) and IV iron administration ($r = 0.33$; $P = 0.01$). The levels of Gas6 rose with CKD stage and were inversely associated with estimated GFR ($P < 0.0001$).

Conclusions. Dysregulation of circulating Gas6 is associated with renal disease and inversely proportional to renal function. Low albumin and higher IV iron administration were associated with higher Gas6 levels, suggesting a possible connection between inflammation and oxidative stress mediated by iron. Protein S levels were also elevated in CKD patients, but the relevance of this finding needs to be further investigated.

Keywords: chronic kidney disease; dialysis; Gas6; inflammation; mortality; vascular disease

Introduction

Morbidity and mortality rates remain very high in chronic kidney disease (CKD). In CKD, the overall mortality rate is up to 30-fold higher than age-adjusted mortality in the general population [1–3]. Vascular disease, anemia, chronic inflammation and malnutrition are more prevalent in patients with CKD. Despite knowledge that CKD is associated with a state of chronic inflammation and with elevated levels of pro-inflammatory cytokines, both

© The Author 2012. Published by Oxford University Press on behalf of ERA-EDTA. All rights reserved.
For Permissions, please e-mail: journals.permissions@oup.com

Downloaded from http://ndt.oxfordjournals.org/ at Xi'an Jiaotong University on April 19, 2016
