# Supplementary material for: Association of Renalase SNPs rs2296545 and rs2576178 with the Risk of Hypertension: A Meta-Analysis
Source: PLoS One. 2016 Jul 19;11(7):e0158880. doi: 10.1371/journal.pone.0158880 (PMC4951046; doi:10.1371/journal.pone.0158880)
Supplement: S3 File — (ZIP) [file pone.0158880.s003.zip › 11 excluded records/Qi Zhao et al. 2007.pdf]

J Mol Med (2007) 85:877–885
DOI 10.1007/s00109-006-0151-4

ORIGINAL ARTICLE

Renalase gene is a novel susceptibility gene for essential hypertension: a two-stage association study in northern Han Chinese population

Qi Zhao · Zhongjie Fan · Jiang He · Shufeng Chen · Hongfan Li · Penghua Zhang · Laiyuan Wang · Dongsheng Hu · Jianfeng Huang · Boqin Qiang · Dongfeng Gu

Received: 24 July 2006 / Revised: 19 September 2006 / Accepted: 2 November 2006 / Published online: 10 January 2007
© Springer-Verlag 2007

Abstract Renalase, a novel flavin adenine dinucleotide-dependent amine oxidase, is secreted by the kidney, degrades circulating catecholamines, and modulates cardiac function and systemic blood pressure (BP). Its discovery may provide novel insights into the mechanisms of BP regulation and the pathogenesis of essential hypertension (EH). We designed a two-stage case-control study to investigate whether the renalase gene harbored any genetic variants associated with EH in the northern Han Chinese population. From the International Collaborative Study of Cardiovascular Disease in Asia (InterASIA in China), 1,317 hypertensive cases and 1,269 normotensive controls were recruited. These total 2,586 subjects were taken as the main study population in this study. In stage 1, all the eight selected single nucleotide polymorphisms (SNPs) of the

Q. Zhao · S. Chen · H. Li · P. Zhang · L. Wang · J. Huang · D. Gu (✉)
Department of Evidence Based Medicine and Division of Population Genetics, Cardiovascular Institute and Fuwai Hospital, Chinese Academy of Medical Sciences and Peking Union Medical College,
No. 167 Beilishi Road,
Beijing 100037, China
e-mail: gudf@yahoo.com

Q. Zhao · B. Qiang · D. Gu
National Human Genome Center at Beijing,
North Yongchang Rd 3-707,
Beijing 100176, China

Z. Fan
Department of Cardiology,
Peking Union Medical College Hospital,
Beijing 100730, China

J. He
Tulane University Medical Center,
New Orleans, LA 70112-2699, USA

D. Hu
Department of Epidemiology, College of Public Health,
Zhengzhou University,
Zhengzhou, Henan 450052, China

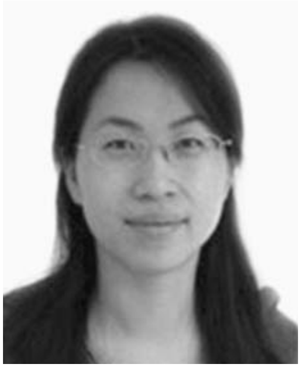

QI ZHAO
received her B.S. degree in preventive medicine from Peking University Health Science Center in Beijing, China. She is currently a Ph.D. student of Prof. Gu at Department of Evidence Based Medicine and Division of Population Genetics, Fuwai Hospital, Chinese Academy of Medical Sciences and Peking Union Medical College in Beijing, China. Her research interests include identification of genetic factors for essential hypertension.

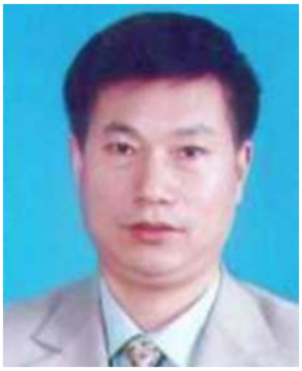

DONGFENG GU
is Professor of Epidemiology and Medical Genetics, Chair of Department of Evidence Based Medicine and Division of Population Genetics at Fuwai Hospital, Chinese Academy of Medical Sciences and Peking Union Medical College in Beijing, China. He received his medical training in Nanjing Medical University and Peking Union Medical College in Beijing and did his postdoctoral training at University of Minnesota and University of Southampton. His research interests in the genetic field include identification of genetic factors and gene–environmental interaction for major cardiovascular and related diseases.

renalase gene were genotyped and tested within a subsample (503 cases and 490 controls) of the main study population. By single locus analyses, three SNPs, rs2576178, rs2296545, and rs2114406, showed significant associations with EH ($P<0.05$). In stage 2, these three SNPs were genotyped on the remaining individuals and analyzed using all the individuals. After Bonferroni correction for multiple comparisons, the associations of rs2576178 and rs2296545 with EH were still significant in stage 2. The cases had higher frequencies of rs2576178 G allele and rs2296545 C allele than the controls (0.55 versus 0.49, $P<0.0001$; 0.61 versus 0.55, $P<0.0001$). Particularly, under the codominant model, the adjusted odds ratios for rs2576178 GG genotype and rs2296545 CC genotype were 1.58 (95% CI, 1.25 to 2.00; $P=0.0002$) and 1.61 (95% CI, 1.26 to 2.04; $P=0.0002$), respectively. We also found risk-associated haplotypes and diplotypes, which further confirmed the significant association between the renalase gene and EH. These findings may provide novel genetic susceptibility markers for EH and lead to a better understanding of EH pathophysiology. In addition, further replications in other populations and functional studies would be warranted.

Keywords Case-control studies · Hypertension · Kidney · Monoamine oxidase · Single nucleotide polymorphism

Introduction

Hypertension is not only a disease but a major risk factor for cardiac, brain, and kidney pathology as well [1, 2]. Essential hypertension (EH) accounts for approximately 90–95% of patients diagnosed with hypertension. It is widely believed that EH is a complex disease influenced by multiple factors. Inherited predisposition combines with environmental factors to determine the manifestation and severity of this disorder [3]. The genetic element contribution to blood pressure (BP) variation ranges from 30 to 50% [4, 5]. The genetic background of EH is complex and currently not fully understood. Some candidate genes, which were selected based on known mechanisms of BP regulation, have shown association with EH [6]. However, their variants associated with EH cannot fully explain the genetic component of EH risk. One possibility is that some biological mechanisms regulating BP level are still unknown to people. Therefore, the discovery of new molecules and pathways involved in BP regulation may lead to the identification of novel gene sequence variations participating in the susceptibility of EH.

Renalase is a novel flavin adenine dinucleotide (FAD)-dependent amine oxidase and has been recently discovered by Xu et al. [7]. They found that renalase was secreted by the kidney, degraded catecholamines, and regulated systemic BP. The catecholamines include such compounds as norepinephrine, epinephrine, and dopamine. It is well known that catecholamines can regulate heart rate, myocardial contractility, and the tone of resistance vessels and thus play an important role in controlling BP. Renalase injection to Sprague-Dawley rats elicited a rapid depressor response with a maximal decrease in mean arterial pressure of almost 30%. The BP lowering effect of renalase was explained by the degradation of circulating catecholamines, which would be expected to decrease cardiac contractility and heart rate. Certainly, the possibility could not be excluded that the hypotensive effect of renalase might be partly receptor-mediated.

As renalase is produced within the kidney, its major function possibly resides within the organ [8]. Xu et al. [7] found that dopamine was the preferred substrate of renalase, followed by epinephrine and norepinephrine. Dopamine produced locally is important in the paracrine/autocrine regulation of renal tubular sodium transport [9–11]. Renal sodium reabsorption is a critical process serving to maintain both extracellular fluid volume and arterial BP. Proteins participating in sodium reabsorption and its regulation are therefore important candidate proteins whose genes may contain sequence variations contributing to the inherited tendency for EH [12]. It is possible that renalase can regulate salt and water excretion to influence BP level by directly metabolizing dopamine within the kidney. Therefore, the discovery of renalase may enhance knowledge of the role of the kidney in BP control and lead to the identification of novel genetic susceptibility markers for EH.

The main objective of our study was to investigate whether the renalase coding gene was associated with EH in a northern Han Chinese population. Recently, Satagopan et al. proposed a two-stage design for association study that could provide near-optimal power to detect the true marker conferring disease risk while substantially reducing the total number of marker evaluations [13, 14]. In the present study, we conducted a two-stage case-control study which was similar but not identical to the approach proposed by Satagopan et al. In stage 1, all eight selected single nucleotide polymorphisms (SNPs) of renalase gene were genotyped and tested in a relatively small case-control subsample of the main study population. Initial associations found in stage 1 were taken as hypotheses that were further tested and analyzed in stage 2 using the main study population.

Materials and methods

Subjects and strategy

All the studied subjects were recruited from the International Collaborative Study of Cardiovascular Disease in

Asia (InterASIA in China). InterASIA used a four-stage stratified sampling method to select a nationally representative sample of the general population aged 35 to 74 years in China. A total of 15,838 persons completed the survey and examination [15]. InterASIA stratified China into north and south, as delineated by the Yangtze River. In this study, we enrolled 1,317 hypertensives (655 men and 662 women) with systolic BP (SBP)≥150 mmHg, diastolic BP (DBP)≥ 95 mmHg, or current use of antihypertensive medication and 1269 healthy normotensives (658 men and 611 women) with SBP<140 mmHg and DBP<90 mmHg from the northern field centers of InterASIA, namely, Beijing, Jilin, Shandong, and Shanxi. These total 2,586 subjects were unrelated and taken as the main study population in this study.

We adopted a two-stage association study strategy. From the main study population, we selected 993 subjects as a subsample containing 503 hypertensive patients (SBP≥ 160 mmHg and/or DBP≥100 mmHg) and 490 age- and gender-matched normotensive controls (SBP<140 mmHg and DBP<90 mmHg). The criterion for case selection of the subsample was based on the hypothesis that individuals with higher BP were likely to be enriched for genetic susceptibility, which might increase the difference in frequency of susceptibility alleles between cases and controls to improve power. Under this two-stage approach, all the selected SNPs were genotyped and tested at stage 1 using the subsample, and the promising SNPs were genotyped on the remaining individuals and tested using the main study population at stage 2.

During clinic or home visits, trained research staff administered a standard questionnaire. They obtained information on demographic characteristics including age, gender, ethnicity, education, occupation, and household income. The interview also included questions related to the diagnosis and treatment of hypertension [16]. Three BP measurements were obtained from each participant by trained and certified observers according to a standard protocol recommended by the American Heart Association [17]. BP was measured with the participant in the sitting position after 5 min of rest. In addition, participants were advised to avoid alcohol, cigarette smoking, coffee/tea, and exercise for at least 30 min before their BP measurement. Subjects with a clinical history of secondary hypertension, coronary heart disease, diabetes, and chronic kidney disease were excluded from the study. The protocol was approved by the local bioethical committee, and informed consent was obtained from each participant.

SNP selection

The renalase gene is located on chromosome 10 at q23.31 and spans 309,388 bp with nine exons (official symbol: C10orf59). There were 749 entries of SNPs for the renalase gene in the public NCBI Single Nucleotide Polymorphism database (dbSNP, build126; available at http://www.ncbi.nlm.nih.gov/SNP/; last accessed May 25, 2006), and 389 of them had available frequency data among Han Chinese in Beijing, China (CHB) from the International HapMap project website http://www.hapmap.org/; HapMap Public Release #20/phaseII; last accessed May 25, 2006). The renalase gene SNPs were selected based on the following criteria: minor allele frequency (MAF)≥0.05 in CHB validated by the HapMap and location in putative functional regions of the gene (e.g., exons, gene flanking regions, and exon/intron boundaries). Under these criteria, eight SNPs were selected for genotyping in stage 1 (Table 1), and their relative physical positions are presented in Fig. 1. SNP rs2296545 was the only nonsynonymous-coding SNP in the renalase gene at the time of database research, which resulted in an amino acid substitution (aspartic acid to glutamic acid at codon 37, Asp37Glu) and might affect the function of the gene product. The other SNPs were located at flanking regions or near exon/intron boundaries where gene variants might cause variation in gene regulation and expression or differential splicing. Our goal was not to investigate all of the renalase gene sequence variation but to generate a set of markers that could partly represent the functional region of the gene.

Genotyping

Blood for genotyping was taken into ethylenediamine tetraacetic acid (EDTA)-containing receptacles; DNA was isolated according to a standard phenol-chloroform method and stored at −20°C until required for batch genotyping. All SNPs were genotyped according to standard polymerase chain reaction and restriction fragment length polymorphism methods. The primers and related restriction endonuclease can be obtained by request. Ninety-six randomly

Table 1 Selected SNPs in renalase gene region

SNP	dbSNP accession number	Region	Alleles[a]	MAF[b]
1	rs2576178	5′ flanking	G/A	0.48
2	rs2296545	Exon 2	C/G	0.44
3	rs2765446	Intron 4	T/C	0.46
4	rs11202776	Intron 5	C/T	0.12
5	rs1648512	Intron 6	A/G	0.32
6	rs10887800	Intron 6	A/G	0.50
7	rs1035796	Intron 7	C/T	0.47
8	rs2114406	3′ flanking	A/G	0.22

MAF minor allele frequency
[a] Major/minor allele
[b] MAF in the controls of stage 1 study

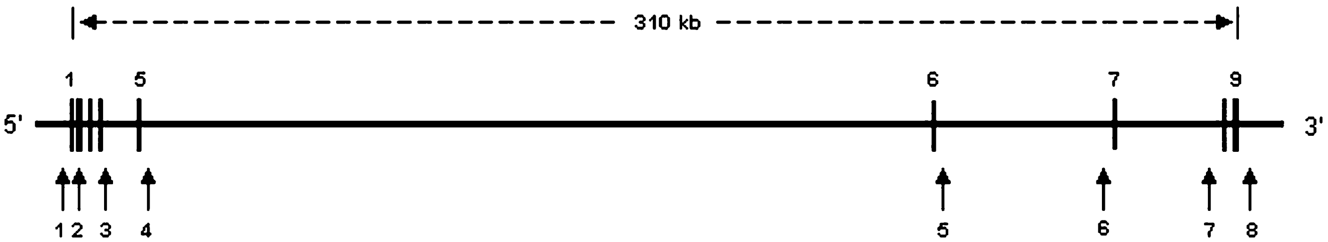

Fig. 1 Genomic structure of the human renalase gene and location of the eight genotyped SNPs in stage 1 study. Exons are shown as *vertical lines*. The locations of SNPs are indicated by *arrows* and they are denoted *numerically* with reference to Table 1

selected individuals were genotyped again for quality control with complete concordance.

Statistical analysis

In stage 1, we used single locus analyses to detect initial associations between the eight tested SNPs and EH. The alleles and genotypes of the SNPs were counted, and their distributions between the case and control groups were compared by the χ^2 test. The criterion used to select SNPs for stage 2 study was that the SNPs had a P value of <0.05 for the comparisons of allele frequencies or genotype distributions (under 2-*df* codominant, 1-*df* dominant, and 1-*df* recessive models) between the cases and the controls of stage 1. Because stage 1 served for hypothesis generation, correction for multiple comparisons was not done.

In stage 2, all statistical analyses were performed using the main study population. In single locus analyses, a stepwise logistic regression was conducted to adjust for covariates including age, gender, body mass index (BMI), glucose (Glu), triglycerides (TG), total cholesterol (TC), high density lipoprotein cholesterol (HDL-C), creatinine (Cr), and smoking and drinking status. Multiple testing was adjusted using Bonferroni correction. Multilocus analyses including haplotype and diplotype analyses were also performed in stage 2. To test the associations of statistically inferred haplotypes with EH, we used the Haplo.score approach as outlined by Schaid et al. [18]. The method models an individual's phenotype as a function of each inferred haplotype, weighted by their estimated probability, to account for haplotype ambiguity. To obtain odds ratios (ORs) of risk haplotypes, the Haplo.glm approach was performed [19]. Both Haplo.score and Haplo.glm were implemented in the Haplo.stats software. A diplotype analysis was then followed by using a weighted logistic regression, with the weights being the probability for each possible haplotype pair combination for an individual as estimated by Haplo.score. Only the haplotypes and diplotypes with frequency >5% were considered for the haplotype and diplotype analyses, respectively.

Descriptive statistical analyses were performed with Statistical Analysis Software (SAS; SAS Institute, Cary, NC, USA). Hardy–Weinberg equilibrium (HWE) of the SNPs was evaluated by Fisher's exact test using the program HWE [20]. The pattern of pairwise linkage disequilibrium (LD) between the SNPs was measured by D' and r^2 calculated by the program Haploview [21].

Results

Clinical characteristics

Table 2 shows the characteristics of the subjects included in the stage 1 and stage 2 studies. Age and the percentage of men were not significantly different between cases and controls in both studies. The cases generally had lower HDL-C and higher BMI, TC, TG, and Glu levels than the corresponding controls. In both studies, there were no significant differences in the prevalence of drinking and smoking between cases and controls. As expected, the SBP and DBP levels were both significantly higher in the cases of stage 1 than those of stage 2 (both P values<0.0001).

Single locus analyses in stage 1 study

During stage 1, which served for hypothesis generation, we evaluated all eight selected SNPs in the subsample. A graphical representation of pairwise LD as measured by $|D'|$ is displayed in Fig. 2. Table 3 summarizes the genotype and allele distributions of the eight SNPs among the cases and controls involved in stage 1. All SNPs were in HWE in both cases and controls. We found that the frequencies of rs2576178 G allele and rs2296545 C allele in the cases were significantly higher than those in the controls (both P values<0.05). The differences in allele frequencies of rs2114406 between the case and control groups showed a trend toward statistical significance (P=0.073) and a modest dominant effect of rs2114406 G allele was also found (P=0.047). According to the criterion defined for stage 1, these three SNPs, rs2576178, rs2296545, and

Table 2 Characteristics of the subjects in stage 1 and stage 2 studies

Characteristics	Stage 1		Stage 2	
	Cases (n=503)	Controls (n=490)	Cases (n=1317)	Controls (n=1269)
Male (%)	52.1	52.4	49.7	51.9
Age (years)	53.6±9.3	53.5±9.2	54.2±10.2	53.5±9.5
SBP (mmHg)	177.07±28.05*	117.47±11.64	159.70±26.13*	115.21±10.72
DBP (mmHg)	104.34±12.28*	75.05±8.00	95.85±12.70*	73.69±7.81
BMI (kg/m^2)	26.32±3.85*	24.30±3.56	25.99±3.64*	24.02±3.44
TC (mmol/l)	5.23±0.99****	5.06±1.05	5.14±0.97*	4.93±1.04
HDL-C (mmol/l)	1.25±0.30***	1.32±0.34	1.25±0.30**	1.29±0.32
LDL-C (mmol/l)	3.19±0.86	3.09±0.87	2.81±1.29****	2.71±1.23
TG (mmol/l)	1.70±1.06*	1.43±0.86	1.61±0.94*	1.40±0.89
Glu (mmol/l)	5.93±1.79***	5.60±1.68	5.60±1.24*	5.38±1.20
Cr (μmol/l)	71.22±14.59****	69.20±11.57	70.85±14.08	70.18±12.67
Smokers (%)	40.6	43.1	40.0	43.0
Drinkers (%)	34.4	33.5	31.9	30.5

Mean ± standard deviation values for continuous variables

SBP systolic blood pressure, *DBP* diastolic blood pressure, *BMI* body mass index, *TC* total cholesterol, *HDL-C* high density lipoprotein cholesterol, *LDL-C* low density lipoprotein cholesterol, *TG* triglyceride, *Glu* glucose, *Cr* creatinine

*P<0.0001 for comparison with corresponding controls

**P<0.001 for comparison with corresponding controls

***P< 0.01 for comparison with corresponding controls

****P<0.05 for comparison with corresponding controls

rs2114406, would be taken as hypotheses and enter stage 2 study.

Analyses in stage 2 study

In stage 2 study, the three SNPs (rs2576178, rs2296545, and rs2114406) associated with EH in stage 1 were genotyped on the remaining cases and controls and analyzed using all 2,586 individuals who had been taken as the main study population. We performed not only single locus analyses but also multilocus analyses, which included haplotype and diplotype analyses. Through the single locus analyses, we found that rs2576178 G allele and rs2296545 C allele were significantly higher in the cases than in the controls (both P values<0.0001), and the genotype distributions of these two SNPs were also significantly different between the two groups (P<0.0001 and P=0.0001, respectively). However, the association of rs2114406 with hypertension was not replicated in this stage (Table 4). After Bonferroni correction, where was set at 0.0063 (0.05 of 8), rs2576178 and rs2296545 were still significantly associated with EH. Particularly, under the codominant model, the adjusted ORs for EH associated with rs2576178 GG genotype (GG versus AA) and rs2296545 CC genotype (CC versus GG) were 1.58 (95% CI, 1.25 to 2.00; P=0.0002) and 1.61 (95% CI, 1.26 to 2.04; P= 0.0002), respectively (Fig. 3).

In the haplotype analyses, we found that five haplotypes constructed by these three SNPs had the frequencies >0.05 and accounted for 95.5% haplotype variations (Table 5). The adjusted haplotype global score test was significant (P=0.0007, 5 df), and the frequencies of Hap2 (G-C-A) and Hap3 (G-C-G), which consisted of both rs2576178 G and rs2296545 C risk alleles, were significantly higher in the cases than in the controls. With Hap1 (A-G-A) used as the

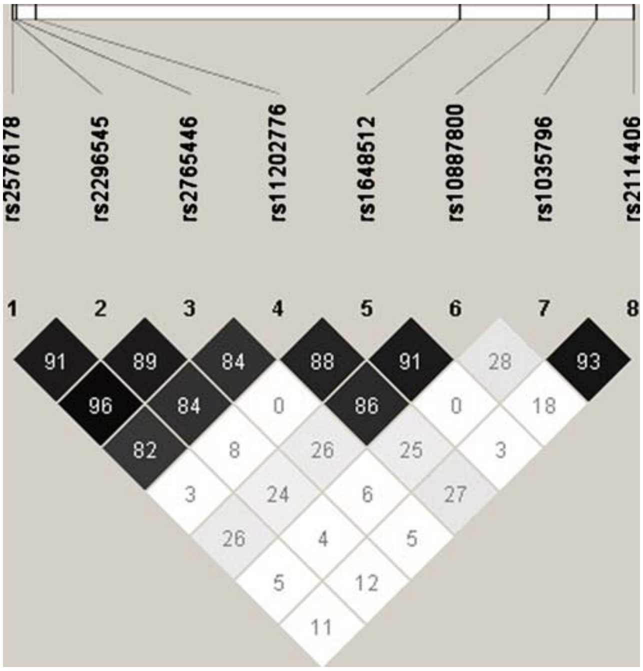

Fig. 2 Linkage disequilibriums among eight SNPs in the controls of stage 1 study. The numbers inside the *squares* are D′×100%

Table 3 Genotype distributions and allele frequencies of the eight SNPs tested in stage 1

	SNP	Genotype	Allele frequency	P value (χ^2-test)			
				Codominant model[a]	Dominant model[b]	Recessive model[b]	Allele
1	rs2576178	GG/GA/AA	G/A	**0.026**	0.090	**0.012**	**0.012**
	Cases	158/263/82	0.58/0.42				
	Controls	130/249/111	0.52/0.48				
2	rs2296545	CC/CG/GG	C/G	**0.034**	0.148	**0.012**	**0.018**
	Cases	182/247/74	0.61/0.39				
	Controls	156/232/102	0.56/0.44				
3	rs2765446	TT/TC/CC	T/C	0.436	0.516	0.209	0.265
	Cases	149/268/86	0.56/0.44				
	Controls	136/255/99	0.54/0.46				
4	rs11202776	CC/CT/TT	C/T	0.896	0.707	0.729	0.673
	Cases	390/108/5	0.88/0.12				
	Controls	375/109/6	0.88/0.12				
5	rs1648512	AA/AG/GG	A/G	0.264	0.907	0.114	0.406
	Cases	225/215/63	0.66/0.34				
	Controls	221/223/46	0.68/0.32				
6	rs10887800	AA/AG/GG	A/G	0.843	0.938	0.597	0.787
	Cases	117/271/115	0.50/0.50				
	Controls	115/256/119	0.50/0.50				
7	rs1035796	CC/CT/TT	C/T	0.578	0.403	0.386	0.300
	Cases	149/254/100	0.55/0.45				
	Controls	133/249/108	0.53/0.47				
8	rs2114406	AA/AG/GG	A/G	0.136	**0.047**	0.683	0.073
	Cases	283/186/34	0.75/0.25				
	Controls	306/154/30	0.78/0.22				

Dominant and recessive models were based on minor allele of each locus. *P* values<0.05 are shown in bold.
[a]With degree of freedom 2
[b]With degree of freedom 1

reference, the adjusted ORs for EH associated with Hap2 and Hap3 were 1.20 (95% CI, 1.03 to 1.41; *P*=0.0220) and 1.46 (95% CI, 1.20 to 1.78; *P*=0.0002), respectively. Similarly, diplotype analyses showed that compared with Dip1 (Hap1-Hap1), the ORs for Dip2 (Hap1-Hap2), Dip3 (Hap2-Hap2), and Dip4 (Hap2-Hap3) were 1.56 (95% CI, 1.13 to 2.15; *P*=0.0071), 1.64 (95% CI, 1.15 to 2.34; *P*=0.0066), and 1.88 (95% CI, 1.28 to 2.78; *P*=0.0014).

Discussion

By the two-stage association study, we found that the renalase coding gene was a novel susceptibility gene for EH. No other study to date has assessed the relationship between renalase gene variation and hypertension. These findings may lead to a novel insight into the mechanisms of BP regulation and the pathogenesis of hypertension.

Our two-stage case-control study design was similar but not identical to the approach proposed by Satagopan et al. [14]. Using simulations, they showed that for a given sample size, when the markers were independent or correlated, a two-stage design could provide near-optimal power to detect the true marker conferring disease risk while substantially reducing the total number of marker evaluations. In particular, evaluating all the markers on 50% of the individuals in stage 1 and evaluating the most promising 10% of the markers on the remaining individuals in stage 2 provides a practical cost-effective strategy for association studies. Although this is only a general guideline, it is helpful to improve association study design for disease gene mapping. On the other hand, the correct

Table 4 Genotype distributions and allele frequencies of the three SNPs tested in stage 2

	Cases (*n*=1317)	Controls (*n*=1269)	*P* value
rs2576178			
GG	367	298	
GA	702	641	
AA	242	321	<0.0001
G allele frequency	0.55	0.49	<0.0001
rs2296545			
CC	481	391	
CG	641	619	
GG	193	257	0.0001
C allele frequency	0.61	0.55	<0.0001
rs2114406			
GG	98	81	
GA	475	452	
AA	741	734	0.5059
G allele frequency	0.26	0.24	0.2794

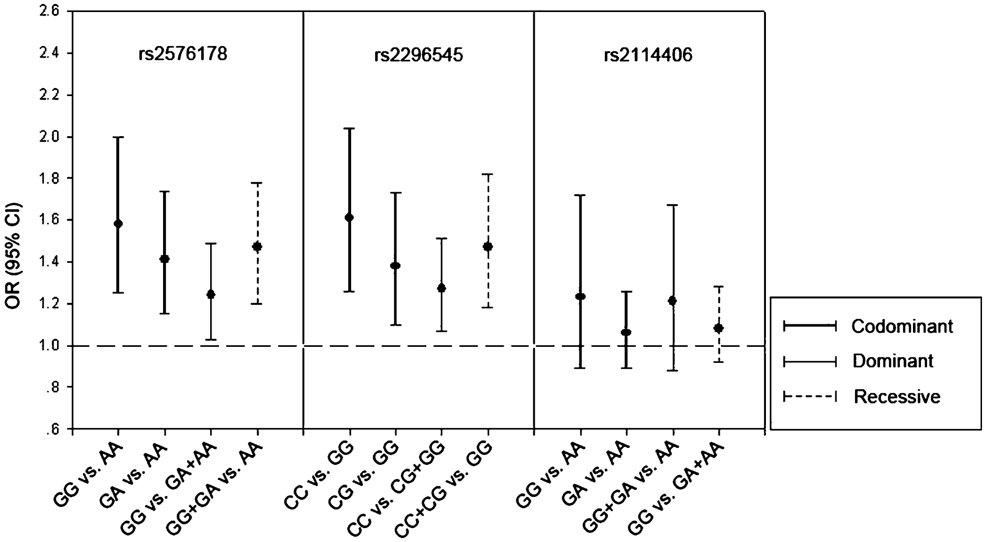

Fig. 3 Adjusted odd ratios (*ORs*) for EH associated with genotypes of the three SNPs tested in stage 2. *CI* indicates confidence interval. ORs were adjusted for age, gender, BMI, TC, HDL-C, TG, Glu, Cr, and drinking and smoking status. Dominant and recessive models were based on minor allele of each locus as listed in Table 1. The 95% CI *lines* crossing the OR line of 1 indicate no significance

and powerful strategy depends on disease-specific and study-specific factors, which are both known (e.g., the cost) and unknown (e.g., the genetic architecture of the disease). In our study, the individuals used in stage 1 were less than half of the total study individuals, which might decrease the power to some extent. However, the hypertensive cases used in stage 1 had higher BP level and were likely to be enriched for genetic susceptibility, which might increase the difference in frequency of susceptibility alleles between cases and controls to improve the power. In addition, when we used an uncorrected $P<0.05$ as the criterion, three SNPs, more than 10% of the total markers, were selected for stage 2 study, which might further maintain the power. Of course, the total number of variants we tested was much less than that simulated by Satagopan et al., so the criterion that would be used to evaluate the most promising 10% of the markers in stage 2 was inappropriate to our study.

In stage 1, three SNPs (rs2576178, rs2296545, and rs2114406) showed significant associations with EH in a subsample containing 503 hypertensive cases and 490 normotensive controls. There is significant but not perfect LD between rs2576178 and rs2296545 (r^2=0.719). Commonly, pairwise r^2 threshold of 0.8 between two markers is used in selecting tag-SNPs which will be genotyped and can predict effects of untyped SNPs. With a lower r^2 threshold, the power of prediction will decrease rapidly. Considering this point, we tested both rs2576178 and rs2296545 in stage 2. In the single locus analyses, rs2576178 and rs2296545 still showed significant associations with EH in the total 2,586 study subjects, whereas rs2114406 did not any more. SNPs rs2576178 and rs2296545 are located in the 5′ flanking region and in the exon 2 of renalase gene, respectively. There are at least three possibilities for the associations between these two SNPs and EH: (1) both SNPs are functional and affect the expression or activity of renalase protein; (2) one SNP is nonfunctional, and its association with EH is due to the tight LD with the other functional SNP; or (3) both SNPs are nonfunctional and in LD with another functional variant

Table 5 Associations between haplotypes, diplotypes, and EH

Variables		All	Cases	Controls	OR (95% CI)	*P* value*
	Haplotype[a]					
Hap1[b]	A-G-A	0.304	0.284	0.324	–	–
Hap2	G-C-A	0.361	0.377	0.344	1.20(1.03–1.41)	0.0220
Hap3	G-C-G	0.134	0.147	0.120	1.46(1.20–1.78)	0.0002
Hap4	A-G-G	0.089	0.083	0.096	NS	NS
Hap5	A-C-A	0.067	0.065	0.068	NS	NS
	Diplotype					
Dip1[c]	Hap1-Hap1	0.090	0.071	0.109	–	–
Dip2	Hap1-Hap2	0.235	0.241	0.229	1.56(1.13–2.15)	0.0071
Dip3	Hap2-Hap2	0.128	0.135	0.120	1.64(1.15–2.34)	0.0066
Dip4	Hap2-Hap3	0.090	0.102	0.078	1.88(1.28–2.78)	0.0014
Dip5	Hap1-Hap3	0.079	0.084	0.075	NS	NS
Dip6	Hap2-Hap4	0.063	0.063	0.063	NS	NS
Dip7	Hap1-Hap4	0.050	0.043	0.058	NS	NS

[a]Loci are arranged in the order rs2576178–rs2296545–rs2114406.
[b]Haplotype A-G-A (Hap1) was chosen to be the reference haplotype.
[c]Diplotype Hap1-Hap1 (Dip1) was chosen to be the reference diplotype.
NS not significant
*Covariates were adjusted.

which was untested in our study. SNP rs2296545 results in an aspartate to glutamate at codon 37 (Asp37Glu), which is closely near C terminus of a deduced FAD-binding site of the renalase protein. Renalase is critically dependent on FAD for oxidase activity. Whether Asp37Glu variant has any effect on the FAD-binding properties of renalase should need further functional study.

To assess the combined effect of SNPs on the EH risk and find possible risk haplotypes and diplotypes in the population, we further performed multilocus analyses in stage 2. The results of haplotype-specific score test showed that the frequency of Hap1 (A-G-A) was significantly lower in the cases than in the controls (0.284 versus 0.324, $P=0.0004$). In comparison with the Hap1, Hap2 (G-C-A) and Hap3 (G-C-G) were found to significantly increase the risk of EH. The only difference between Hap2 and Hap3 was at the SNP rs2114406. The OR of Hap3 was a little but not significantly higher than that of Hap2. After we ignored the rare diplotypes (with estimated frequency <0.05), there remained three risk diplotypes, Dip2 (Hap1-Hap2), Dip3 (Hap2-Hap2), and Dip4 (Hap2-Hap3). As expected, Dip4, which contained two risk haplotypes, Hap2 and Hap3, had the highest risk for EH. However, we failed to observe that Dip5 (Hap1-Hap3), consisting of a risk haplotype Hap3, significantly increased the risk of EH. This might be attributed to the relatively low frequency of Dip5, which resulted in decreased power to find its effect.

Any reported genetic association should be interpreted with caution until it is replicated. To control for false-positive findings, several approaches were considered in this study. First, we included only Northern Han Chinese who were ethnically homogeneous, and the subsample of the main study population has shown no population stratification by genomic control method in our previous study [22]. So the positive associations were unlikely to have resulted from population admixture and stratification. Second, we used conservative Bonferroni correction to control the false–positive findings potentially because of the multiple statistical tests. Finally, after correction for a range of covariates, significant associations were still noted between renalase gene variants and EH.

There may be three potential concerns for our study. First, the renalase gene is not a typical candidate gene for EH because renalase is a novel protein and its biological mechanism for BP regulation is not very clear. However, understanding of molecular mechanisms underlying most common diseases is still poor, which itself is one of the main justifications for gene discovery efforts. This point also makes it imprecise to calculate prior odds associated with any given candidate gene [23]. In addition, with the development of large-scale and whole-genome association study, more disease genes which have not been reported to be associated with diseases and are even with unclear molecular functions will be discovered [24]. Therefore, the following issue will focus on how to appropriately interpret those associations. Second, we only tested limited variants in the renalase gene and left a relative large region of introns unexplored. It is possible that, by employing a denser marker map, we may observe some other significantly associated risk SNPs and risk haplotypes and diplotypes. Third, although rs2576178 and rs2296545 were associated with EH, we failed to observe that the BP levels were significantly different among their genotypes in the control group, respectively. It is possible that our sample has not enough power to detect the effects of their genotypes on BP variation. However, the quantitative trait association analyses using data of the controls may be unreliable because the control group was unlikely to be representative of the general population. To further investigate and confirm the associations between continuous traits (e.g. BP, BMI, and glucose) and renalase gene variation, it should be necessary to design new studies that are based on random samples of the general population.

In conclusion, the present association study suggests that genetic variations in the renalase gene may influence the susceptibility of EH in the northern Han Chinese population. These findings will potentially contribute to a better understanding of the mechanism of BP control and the pathogenesis of EH. In addition, replications in other populations and further functional studies are also required to confirm and interpret the association of renalase gene with EH.

Acknowledgment This work was supported by the National Basic Research Program of China (Grant No. 2006CB503805) and the Beijing Natural Science Foundation (Grant No. 7061006).

References

1. Mosterd A, D'Agostino RB, Silbershatz H, Sytkowski PA, Kannel WB, Grobbee DE, Levy D (1999) Trends in the prevalence of hypertension, antihypertensive therapy, and left ventricular hypertrophy from 1950 to 1989. N Engl J Med 340:1221–1227
2. Warlow CP (1998) Epidemiology of stroke. Lancet 352(Suppl 3): SIII1–SIII4
3. Lifton RP (1996) Molecular genetics of human blood pressure variation. Science 272:676–680
4. Tanira MO, Al Balushi KA (2005) Genetic variations related to hypertension: a review. J Hum Hypertens 19:7–19
5. Gimenez-Roqueplo AP, Jeunemaitre X (2003) Genetics and essential hypertension: candidate genes or screening of the whole genome? Arch Mal Coeur Vaiss 96:1089–1095
6. Naber CK, Siffert W (2004) Genetics of human arterial hypertension. Minerva Med 95:347–356
7. Xu J, Li G, Wang P, Velazquez H, Yao X, Li Y, Wu Y, Peixoto A, Crowley S, Desir GV (2005) Renalase is a novel, soluble monoamine oxidase that regulates cardiac function and blood pressure. J Clin Invest 115:1275–1280
8. Luft FC (2005) Renalase, a catecholamine-metabolizing hormone from the kidney. Cell Metab 1:358–360

9. Jose PA, Eisner GM, Felder RA (1998) Renal dopamine receptors in health and hypertension. Pharmacol Ther 80:149–182
10. Jose PA, Eisner GM, Felder RA (2002) Role of dopamine receptors in the kidney in the regulation of blood pressure. Curr Opin Nephrol Hypertens 11:87–92
11. Aperia AC (2000) Intrarenal dopamine: a key signal in the interactive regulation of sodium metabolism. Annu Rev Physiol 62:621–647
12. Doris PA (2000) Renal proximal tubule sodium transport and genetic mechanisms of essential hypertension. J Hypertens 18:509–519
13. Satagopan JM, Elston RC (2003) Optimal two-stage genotyping in population-based association studies. Genet Epidemiol 25:149–157
14. Satagopan JM, Venkatraman ES, Begg CB (2004) Two-stage designs for gene-disease association studies with sample size constraints. Biometrics 60:589–597
15. Gu D, Reynolds K, Wu X, Chen J, Duan X, Reynolds RF, Whelton PK, He J (2005) Prevalence of the metabolic syndrome and overweight among adults in China. Lancet 365:1398–1405
16. Gu D, Reynolds K, Wu X, Chen J, Duan X, Muntner P, Huang G, Reynolds RF, Su S, Whelton PK, He J (2002) Prevalence, awareness, treatment, and control of hypertension in China. Hypertension 40:920–927
17. Perloff D, Grim C, Flack J, Frohlich ED, Hill M, McDonald M, Morgenstern BZ (1993) Human blood pressure determination by sphygmomanometry. Circulation 88:2460–2470
18. Schaid DJ, Rowland CM, Tines DE, Jacobson RM, Poland GA (2002) Score tests for association between traits and haplotypes when linkage phase is ambiguous. Am J Hum Genet 70:425–434
19. Lake SL, Lyon H, Tantisira K, Silverman EK, Weiss ST, Laird NM, Schaid DJ (2003) Estimation and tests of haplotype-environment interaction when linkage phase is ambiguous. Hum Hered 55:56–65
20. Guo SW, Thompson EA (1992) Performing the exact test of Hardy–Weinberg proportion for multiple alleles. Biometrics 48:361–372
21. Barrett JC, Fry B, Maller J, Daly MJ (2005) Haploview: analysis and visualization of LD and haplotype maps. Bioinformatics 21:263–265
22. Gu D, Su S, Ge D, Chen S, Huang J, Li B, Chen R, Qiang B (2006) Association study with 33 single-nucleotide polymorphisms in 11 candidate genes for hypertension in Chinese. Hypertension 47:1147–1154
23. Hattersley AT, McCarthy MI (2005) What makes a good genetic association study? Lancet 366:1315–1323
24. Shiffman D, Ellis SG, Rowland CM, Malloy MJ, Luke MM, Iakoubova OA, Pullinger CR, Cassano J, Aouizerat BE, Fenwick RG, Reitz RE, Catanese JJ, Leong DU, Zellner C, Sninsky JJ, Topol EJ, Devlin JJ, Kane JP (2005) Identification of four gene variants associated with myocardial infarction. Am J Hum Genet 77:596–605
