# Supplementary material for: Association of Renalase SNPs rs2296545 and rs2576178 with the Risk of Hypertension: A Meta-Analysis
Source: PLoS One. 2016 Jul 19;11(7):e0158880. doi: 10.1371/journal.pone.0158880 (PMC4951046; doi:10.1371/journal.pone.0158880)
Supplement: S2 Table — (DOCX) [file pone.0158880.s005.docx]

**Supplement 5.Quality assessment of studies included in the meta-analysis using a modified Newcastle-Ottawa Scale**
