# Supplementary material for: Association of Renalase SNPs rs2296545 and rs2576178 with the Risk of Hypertension: A Meta-Analysis
Source: PLoS One. 2016 Jul 19;11(7):e0158880. doi: 10.1371/journal.pone.0158880 (PMC4951046; doi:10.1371/journal.pone.0158880)
Supplement: S3 File — (ZIP) [file pone.0158880.s003.zip › Reasons for exclusion.docx]

Dear editor,

Thank you for your reminding. Here are 11 excluded records and the reasons for exclusion.

When searching from electronic databases, the following three article records appear both in PubMed and Embase databases, therefore we exclude three and leave the other three for further analysis.

1. “Polymorphism of the renalase gene in end-stage renal disease patients affected by hypertension” by Anna Stec et al.2012

2. “Renalase Gene Polymorphisms in Patients with Type 2 Diabetes, Hypertension and Stroke” by Monika Buraczynska et al. 2011

3. “Renalase gene is a novel susceptibility gene for essential hypertension: a two-stage association study in northern Han Chinese population” by Qi Zhao et al. 2007

The following excluded studies not only appear both in PubMed and Embase databases, but also do not suit inclusion criteria: In Wang et al. article, they do not study rs2296545 and rs2576178; as for Fava et al. article, it is a cohort study, not case-control or nested case–control studies. Thus, these four article records are excluded.

4-5. “Genetic Variants in Renalase and Blood Pressure Responses to Dietary Salt and Potassium Interventions: A Family-Based Association Study” by Yang Wang et al. 2014

6-7. “The Renalase Asp37Glu polymorphism is not associated with hypertension and cardiovascular events in an urban-based prospective cohort: the Malmö Diet and cancer study” by Cristiano Fava et al. 2012

This article record appears three times in electronic databases, twice in Embase and once in PubMed databases, so we exclude two records.

8-9. “Renalase Gene Polymorphism in Patients with Hypertension and Concomitant Coronary Heart Disease” by Xiaogang Li et al. 2014

This Chinese article appears both in CNKI and WangFang Chinese databases, so we exclude one.

10. “Renalase gene polymorphisms in patients with hypertension and coronary heart disease” by LuHong Li et al. 2013 (written in chinese)

To sum up, these ten articles are excluded because of the duplicated records in these databases.

When carefully reading and extracting data from the following study, we find it is a master thesis. Its study population has overlap with a published study titled “Renalase Gene Polymorphism in Patients with Hypertension and Concomitant Coronary Heart Disease” by Xiaogang Li et al. 2014, and these authors come from a same team. The article written in Chinese recruits subjects from the Department of Cardiology of the Third Xiangya Hospital of Central South University from September 2012 to February 2013, while the published study recruits from August 2012 to November 2013, therefore we exclude this Chinese master thesis.

11. “Renalase gene polymorphisms in patients with hypertension and coronary heart disease” by LuHong Li et al. 2013 (written in chinese)

In conclusion, according to the reasons mentioned above, these eleven article records are excluded. Thanks for your reminder. We hope that we have addressed your concerns.
